# Quality, Empathy, and Readability of AI Chatbot Responses to the Survivorship Needs of Adolescents and Young Adults With Melanoma: Evaluation Study

**DOI:** 10.2196/84234

**Published:** 2026-03-26

**Authors:** Jordan Lily Jafarnia, Priscilla Lynne Haff, Reece Philip Moore, Alyssa Leigh Osheim, Katherine McKenna Riley, Sabrina Zheng, Michael Roth, Madeleine Hines Salge, Kelly Carter Nelson

**Affiliations:** 1 UTHealth Houston, McGovern Medical School, 6431 Fannin St, Houston, TX, 77030, United States; 2 Paul L. Foster School of Medicine at Texas Tech University Health Sciences Center El Paso, El Paso, TX, United States; 3 Division of Pediatrics, University of Texas MD Anderson Cancer Center, Houston, TX, United States; 4 Department of Dermatology, Division of Internal Medicine, The University of Texas MD Anderson Cancer Center, 1515 Holcombe Blvd, Houston, TX, 77030, United States, 1 713-745-1113

**Keywords:** melanoma, young adult, artificial intelligence, natural language processing, empathy, readability, adolescent

## Abstract

**Background:**

Melanoma, a highly aggressive form of skin cancer, is the second most common type of cancer for adolescent and young adult (AYA, ages 15-39 years) patients. AYA patients with melanoma may turn to internet sources, especially artificial intelligence (AI) chatbots, to manage uncertainty about prognosis and treatment.

**Objective:**

This study aims to evaluate the quality, empathy, and readability of responses generated by leading AI chatbots when addressing the top unmet needs of AYA patients with melanoma receiving treatment.

**Methods:**

Our research team recently surveyed 152 AYA patients with melanoma using the Needs Assessment Service Bridge, a validated instrument that assesses psychosocial needs for AYA patients with cancer. The survey identified the top 5 needs for advanced AYA patients with melanoma receiving treatment. Each need was reframed into a question and brief clinical history, then entered into each chatbot by 5 individuals who cleared their prequestion and postquestion history. Chatbot responses were evaluated to assess information quality (Global Quality Score [GQS] and DISCERN), accessibility and readability (GQS, Flesch Kincaid Grade Level, Flesch Reading Ease), and perceived empathy (Perceived Empathy of Technology Scale [PETS], including domains of Emotional Responsiveness [PETS-ER], Understanding and Trust [PETS-UT]).

**Results:**

Across 75 chatbot responses, ChatGPT achieved the highest average quality (mean GQS 4.42, SD 0.32; mean DISCERN 3.24, SD 0.31) and empathy (mean PETS-ER 5.35, SD 1.85; mean PETS-UT 6.36, SD 1.83), though with greater variability. Copilot produced the lowest quality and empathy scores, while Gemini responses were consistently midrange. PETS-UT exceeded PETS-ER across all models, suggesting stronger cognitive empathy than emotional responsiveness. Readability analysis showed outputs exceeded the average US reading level (mean Flesch Kincaid Grade Level 11.82, SD 1.44; mean FRE 38.60, SD 9.00), limiting accessibility. The most readable responses were found in question 2, which also scored higher in quality and empathy, whereas questions 4 and 5 produced the most complex, difficult-to-read responses corresponding with lower quality and empathy ratings.

**Conclusions:**

AI chatbots can provide moderately accurate and supportive responses to needs of AYA patients with melanoma, but outputs are inconsistent, written above the recommended reading level for health information, and limited in empathy. Question framing strongly influenced chatbot performance, with more emotional prompts drawing greater empathy, and readability aligning with both quality and empathy. Chatbot use in this population should remain adjunctive, with further research needed to standardize quality, improve readability, and enhance empathetic communication.

## Introduction

Melanoma, a malignant tumor of melanocytes, is an aggressive form of skin cancer due to its high metastatic potential and associated mortality. Despite being most frequently diagnosed in older adults, cutaneous melanoma accounts for approximately 7% of newly diagnosed cancers among adolescents and young adults (AYAs, age 15‐39 years) [1]. A melanoma diagnosis and subsequent treatment can be highly disruptive for AYA patients, with implications for physical health, emotional well-being, and social function. The severity of the disease, coupled with the uncertainty of treatment outcomes, often introduces feelings of fear and isolation [2]. In seeking clarity in this uncertainty, patients may turn to the internet and social media for answers, education, and support. When seeking answers through internet interfaces, patients are likely to leverage artificial intelligence (AI)–driven chatbots, such as Chat GPT, Microsoft Copilot, and Google Gemini [3].

AI chatbots use natural language processing to generate conversational responses to user queries and have been shown to provide health information in an accessible and interactive manner [4]. Their availability, objectivity, and capacity to adapt personalized responses to diverse communication styles make them appealing to patients navigating difficult diagnoses [5]. While some studies have evaluated the most frequently asked chatbot questions regarding specific forms of cancer (ie, breast, leukemia, prostate), no published research to our knowledge has systematically examined their ability to address AYAs’ specific concerns regarding melanoma treatment and survivorship [6].

A recent survey study of AYAs with melanoma identified several unmet needs for patients in different phases of cancer care using the validated Needs Assessment Service Bridge (NA-SB) questionnaire [7]. This survey represents a separate study and included 152 AYA respondents, 20 of whom were actively receiving systemic treatment at the time of participation; detailed methods and results are reported elsewhere [7]. For AYA patients with melanoma currently undergoing systemic treatment, the top 5 unmet needs included the following: (1) what happens after treatment, (2) fear of cancer recurrence, (3) long-term hormonal side effects, (4) long-term systemic side effects, and (5) genetic implications on diagnosis and treatment [7]. In this study, survey findings were used solely to identify patient-prioritized unmet needs for chatbot evaluation.

Building on these empirically derived patient priorities, this study anchors chatbot prompts in validated unmet needs rather than search-derived queries; evaluates perceived empathy using the Perceived Empathy of Technology Scale (PETS), a recently validated instrument not previously applied in oncology chatbot research; and examines how readability, empathy, and information quality interact. Together, this novel approach enables a patient-centered and multidimensional evaluation of chatbot suitability for AYA melanoma care, further building on previous chatbot-oncology research. With these established unmet needs and the increasing use of AI chatbots for AYA patient support, we evaluated the unique response of 3 chatbots to patient-centered questions to determine if these resources are safe and valid for use by AYA patients with melanoma.

## Methods

### Need Selection and Question Creation

The top 5 most frequently reported needs for AYA patients with melanoma receiving systemic treatment were identified through the NA-SB survey. Once identified, each NA-SB prompt was slightly modified into a question format with a standardized and appropriate brief clinical history (Textbox 1). All questions shared a common stem that identified the patient as an AYA with metastatic melanoma, with tailored follow-up questions added to reflect the specific need category.

Textbox 1. **Questions input into each chatbot, based on the 5 most frequently reported needs.**

Questions from the perspective of patients currently on treatment:

1. I am an adolescent/young adult patient with metastatic melanoma and am currently receiving treatment. I want more information about what will happen when treatment finishes.
2. I am an adolescent/young adult patient with metastatic melanoma and am currently receiving treatment. In the last month, I have become more worried about my cancer spreading. What do I need to know and what steps can I take to cope with this fear?
3. I am an adolescent/young adult patient with metastatic melanoma and am currently receiving treatment. How will my treatment affect long-term hormone changes?
4. I am an adolescent/young adult patient with metastatic melanoma and am currently receiving treatment. I want more information about how my genetics may or may not have impacted my diagnosis and treatment.
5. I am an adolescent/young adult patient with metastatic melanoma and am currently receiving treatment. I want more information about the long-term side effects of treatment.

### Study Design

**Figure 1.**
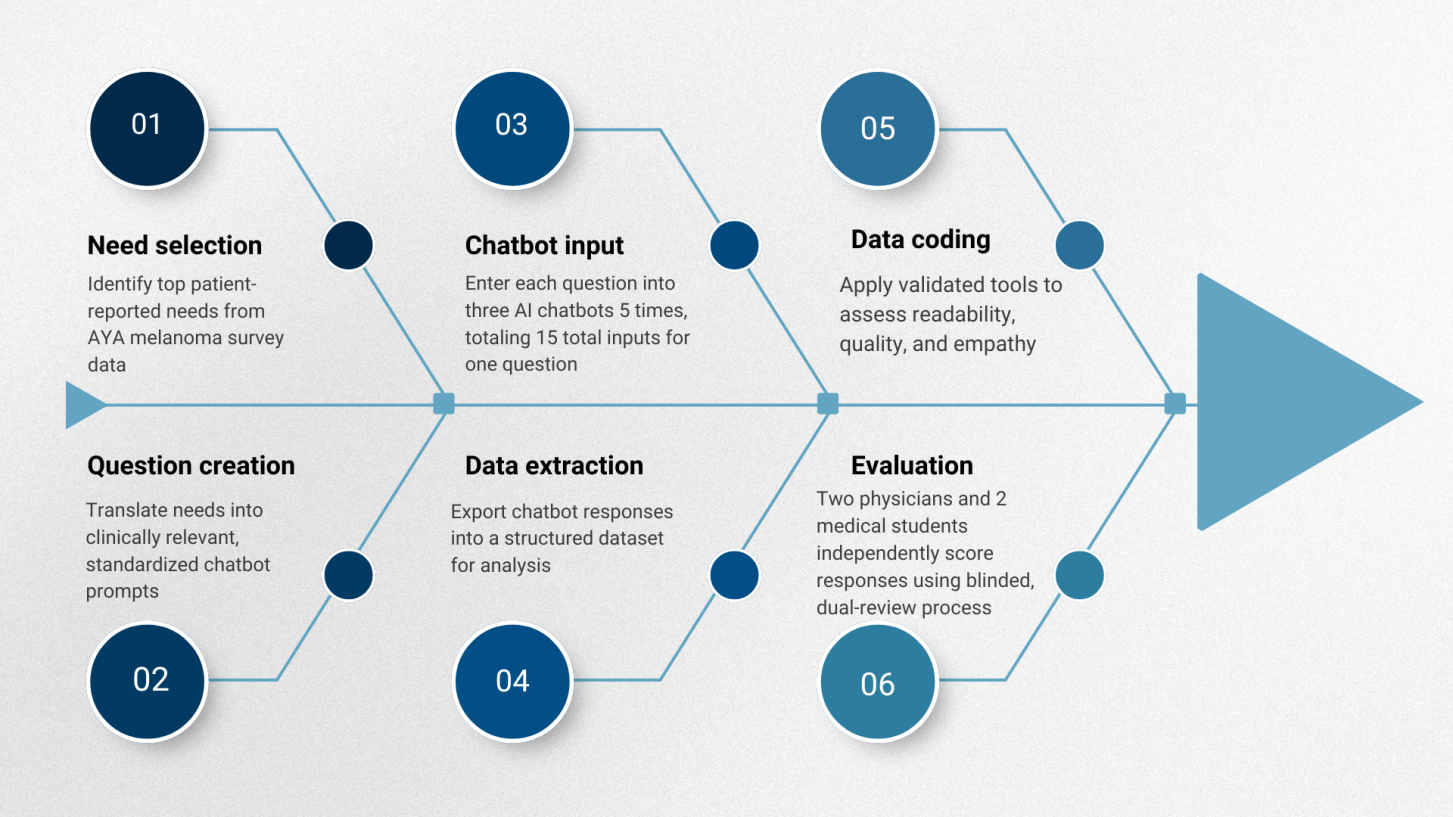
Study design workflow. AI: artificial intelligence; AYA: adolescent and young adult.

This study evaluated the quality of responses generated by AI chatbots to needs frequently reported by AYA patients with melanoma receiving treatment. Our methodologic approach included six components: (1) need selection, (2) question creation, (3) chatbot input, (4) data extraction, (5) data coding, and (6) evaluation (Figure 1).

### Ethical Considerations

This study relied exclusively on publicly available chatbot outputs and did not involve interaction with human participants, collection of identifiable private information, or intervention. As such, it did not meet the definition of human participant research and did not require institutional research board approval.

### Chatbot Input and Data Extraction

Chat GPT 4.0 (Open AI), Copilot (Microsoft), and Gemini (Google) were selected for evaluation based on their increasing usage among AYAs and advancements in their technology. The free, publicly accessible versions of all chatbots were used to reflect the most accessible chatbot platforms available to patients. The resulting need–related questions, outlined in Textbox 1, were entered into each chatbot 5 times by 4 individuals (RPM, ALO, KMR, and SZ) during a single study period (April 8, 2025, to August 8, 2025). To minimize bias, these team members did not participate in question selection, and AI personalization was avoided through memory clearing between each input, ensuring that responses did not draw on prior prompts or stored contextual data, thus replicating a true AYA patient experience. Responses were copied and exported into an Excel spreadsheet where a separate team completed data coding and evaluation.

### Data Coding and Evaluation

Responses were evaluated and coded to assess information quality (Global Quality Score [GQS] and DISCERN), perceived empathy (Perceived Empathy of Technology), and readability (Flesch Kincaid Grade Level [FKGL], Flesch Reading Ease [FRE]; Table 1). Four team members, 2 medical students (JLJ and PLH) and 2 physicians (KCN and MHS), completed evaluations independently, with results hidden until completion to minimize bias. Quality metrics (GQS and DISCERN) were evaluated by physician reviewers due to their clinical expertise, while empathy (PETS) was evaluated by medical student reviewers to approximate a patient-facing perspective. Score interpretation for all tools is provided in Table 1. Reviewers met prior to scoring to align on scale interpretation. Any discrepancy in scores between the raters is a product of the qualitative nature of the rating and is representative of different physicians’ interpretation. All analyses are exploratory and descriptive. Duplicate independent scoring with averaging was used to mitigate individual subjectivity, which was considered sufficient for this evaluation using previously validated instruments. Formal interrater reliability statistics (eg, intraclass correlation coefficient or Cohen κ) were not calculated, as estimates based on only 2 raters per domain may be unstable and potentially misleading, particularly for perceptual constructs such as empathy. The results are presented as means, standard deviations (SD), and visual comparisons. No inferential statistical testing was performed, and no claims of statistical significance or superiority were made.

**Table 1.** Evaluation metrics for chatbot responses, detailing assessed domains and score interpretation.

Assessment tool	Evaluated domains	Scale	Score interpretation
Global Quality Score [8]	Accessibility Quality Flow Usefulness	5-point Likert scale	1=Poor quality, flow, and missing information 2=Fair 3=Moderate 4=Good 5=Excellent quality, flow, and information
DISCERN [9]	Accuracy Quality	Items 1‐8 (5-point Likert scale) Item 16 (summary 5-point Likert scale)	Low quality information ≤2 Medium-quality information=2‐4 High-quality information ≥4
Perceived Empathy of Technology Scale [10]	ER^a^, UT^b^	10 items, 2 domains. Each item is assessed with a 10-point scale and then averaged across 5 items for each domain score (ER and UT)	1‐3=Low empathy 4‐6=Moderate empathy 7‐10=High empathy
Flesch Kincaid Grade Level [11] (FKGL)	Comprehension	Automated formula estimating US school grade level required to understand text	Numeric grade level (eg, 8=8th-grade reading level)
Flesch Reading Ease [11] (FRE)	Readability Flow	Automated formula producing a score from 0 to 100, with higher value indicating easier reading	0‐30=Very difficult to read 31‐50=Difficult to read 51‐60=Fairly difficult to read 61‐70=Plain English 70‐100=Easy to read

a ER: Emotional Responsiveness.

b UT: Understanding and Trust.

### Quality (GQS and DISCERN)

Chatbot answer quality was evaluated using the GQS and DISCERN scales. The GQS scale evaluates quality, accessibility, flow, and perceived usefulness of information for patients, as judged by a physician (Table S1 in Multimedia Appendix 1). The DISCERN scale assesses the quality of written health information, focusing on reliability, clarity, and explanations of risks and benefits (Table S2 in Multimedia Appendix 1). Two physicians (KCN and MHS) evaluated each response with the QGS and DISCERN scales. The results were then averaged into a final score.

### Empathy (PETS)

The PETS, a 10-item instrument designed to assess emotional responsiveness and understanding in human-technology interactions, was used to evaluate perceived empathy in chatbot responses. The PETS was applied without modification. The PETS tool is divided into 2 sections: PETS-ER (6 items) and PETS-UT (4 items; Table S3, Multimedia Appendix 1). The PETS-ER (Emotional Responsiveness) focuses on emotional engagement and support. PETS-UT (Understanding and Trust) measures “cognitive empathy” or the ability of the Chatbot to understand the user’s perspective. Each item was individually scored, then averaged to give domain-specific scores for each input. Two medical student reviewers (JLJ and PLH) performed scoring and highlighted key examples for qualitative evaluation.

### Readability (FKGL, FRE, and Word Count)

To evaluate each output’s readability, the FKGL and FRE were utilized. FKGL estimates the US grade level required to comprehend a response, while FRE scores readability from 0 to 100 (higher=easier to read). Scores for both instruments were calculated in Microsoft Word’s spelling and grammar tool.

## Results

Descriptive metrics of chatbot performance are reported in Table 2, stratified by question with overall summary statistics for each chatbot. Individual input scoring is available in Multimedia Appendix 2.

**Table 2.** Descriptive quality, empathy, and readability metrics for chatbot responses by question and overall.

Question and chatbot	GQS^a^, mean (SD)	DISCERN, mean (SD)	PETS-ER^b^, mean (SD)	PETS-UT^c^, mean (SD)	FRE^d^, mean (SD)	FKGL^e^, mean (SD)	Word count, mean (SD)
Question and chatbot							
1							
ChatGPT	4.50 (0.35)	3.34 (0.30)	6.18 (1.02)	7.80 (0.73)	39.20 (4.77)	12.62 (1.71)	538.80 (66.43)
Copilot	4.00 (0.00)	2.75 (0.09)	5.70 (0.79)	6.45 (0.47)	37.32 (5.68)	11.82 (0.82)	344.80 (54.70)
Gemini	4.00 (0.00)	3.09 (0.14)	5.87 (0.68)	6.50 (1.01)	46.42 (1.20)	11.10 (0.22)	779.80 (69.88)
2							
ChatGPT	4.60 (0.22	3.35 (0.12)	7.53 (0.53)	7.60 (0.80)	54.84 (3.43)	9.10 (0.64)	522.40 (78.60)
Copilot	4.50 (0.35)	3.15 (0.15)	6.67 (0.57)	6.65 (0.38)	46.42 (4.37)	10.72 (0.72)	431.80 (30.91)
Gemini	4.20 (0.27)	3.09 (0.21)	6.67 (0.57)	6.65 (0.38)	52.68 (4.91)	9.80 (0.99)	728.00 (58.94)
3							
ChatGPT	4.00 (0.00)	3.00 (0.00)	3.70 (1.31)	4.18 (0.85)	28.58 (4.78)	11.98 (0.47)	389.20 (48.84)
Copilot	4.10 (0.42)	2.75 (0.10)	4.13 (0.40)	4.10 (0.42)	31.82 (7.22)	12.38 (1.35)	315.20 (47.10)
Gemini	4.50 (0.00)	3.05 (0.11)	4.13 (0.40)	4.10 (0.42)	34.38 (2.51)	13.12 (0.62)	717.80 (71.51)
4							
ChatGPT	4.50 (0.00)	2.94 (0.19)	4.33 (0.41)	6.97 (0.55)	37.62 (1.24)	10.84 (0.30)	545.20 (40.76)
Copilot	3.40 (0.65)	2.55 (0.14)	3.10 (0.37)	3.15 (0.34)	31.72 (4.47)	13.40 (0.65)	360.20 (22.22)
Gemini	4.00 (0.00)	2.81 (0.14)	3.10 (0.37)	3.15 (0.34)	40.60 (4.59	12.16 (0.70)	703.00 (45.08)
5							
ChatGPT	4.40 (0.42)	3.26 (0.50)	5.03 (2.38)	5.30 (2.35)	29.36 (6.90)	12.78 (0.97)	415.60 (178.75)
Copilot	3.70 (0.27)	2.74 (0.19)	2.20 (0.45)	2.70 (0.11)	33.38 (6.52)	12.74 (0.72)	330.80 (34.47)
Gemini	4.20 (0.27)	2.76 (0.03)	2.20 (0.45)	2.70 (0.11)	34.68 (3.20)	12.74 (0.57)	713.60 (50.51)
Overall ratings across all questions							
ChatGPT	4.42 (0.32)	3.24 (0.31)	5.35 (1.85)	6.36 (1.83)	38.9 (10.5)	11.9 (1.64)	482 (111)
Copilot	3.94 (0.53)	2.79 (0.24)	4.36 (1.74)	4.61 (1.72)	36.1 (7.7)	12.3 (1.23)	356 (55)
Gemini	4.18 (0.24)	2.96 (0.19)	4.40 (1.76)	4.62 (1.76)	41.8 (7.9)	11.8 (1.37)	728 (61)

a GQS: Global Quality Score.

b PETS-ER: Perceived Empathy of Technology Scale-Emotional Responsiveness.

c PETS-UT: Perceived Empathy of Technology Scale-Understanding and Trust.

d FRE: Flesch Reading Ease.

e FKGL: Flesch-Kincaid Grade Level.

### Quality (GQS and DISCERN)

**Figure 2.**
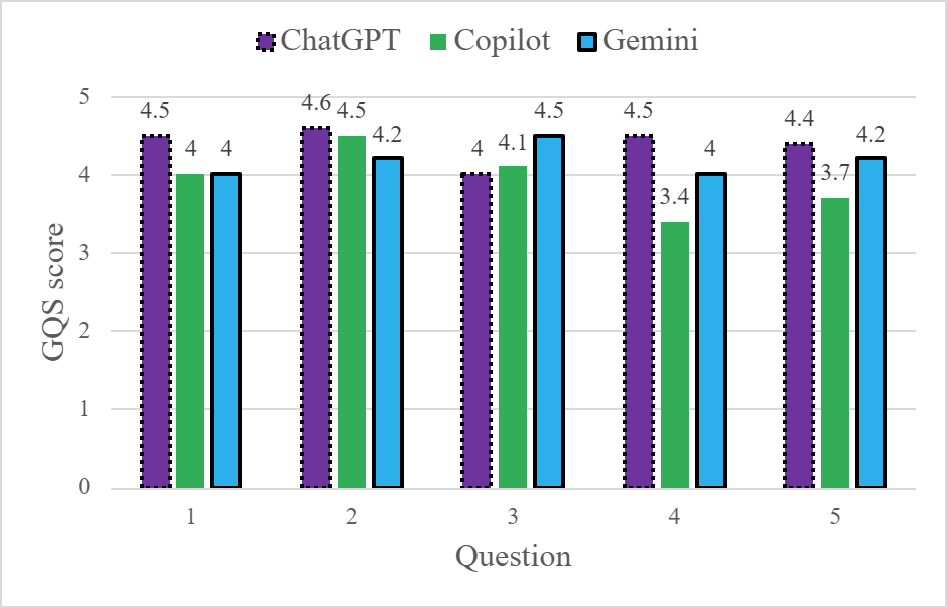
Global Quality Score (GQS) scores comparing each chatbot by question input.

ChatGPT demonstrated the highest average information quality scores, achieving the highest GQS and DISCERN ratings for 4 out of the 5 questions (Figures2  3). Copilot responses showed the lowest average quality scores, while Gemini responses generally fell between the 2 other models.

**Figure 3.**
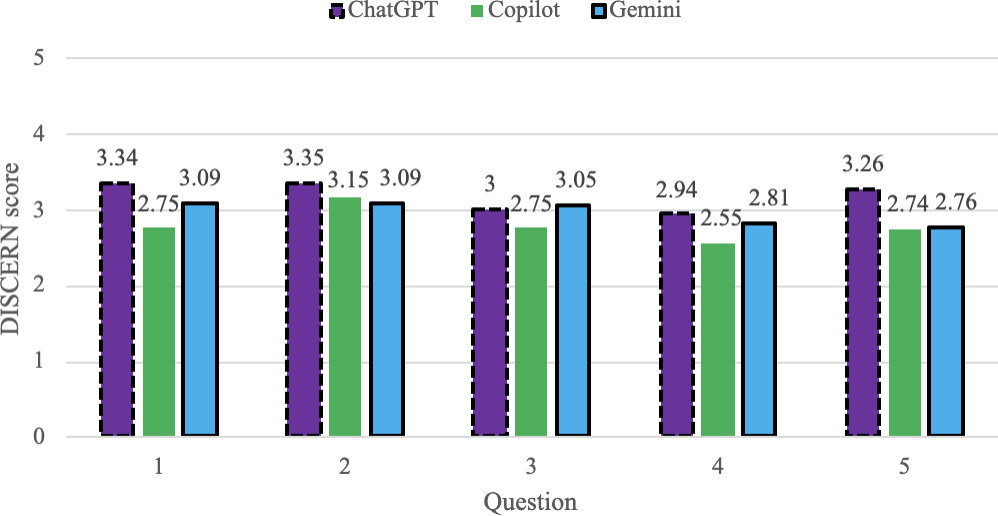
DISCERN scores comparing each chatbot by question input.

Quality variability differed across chatbots (Table 2). Copilot responses demonstrated greater variability in quality scores across question, while Gemini responses showed more consistent quality patterns. ChatGPT exhibited moderate variability for quality metrics. At the question level, Questions 4 and 5 were associated with greater variability in quality scores across chatbots, while Questions 2 and 3 yielded more similar scores across models (see Multimedia Appendix 3 for all SD). Overall, descriptive rank ordering based on average scores and variability suggests differences in consistency and distribution of information quality across chatbots.

### Empathy (PETS-ER and PETS-UT)

**Figure 4.**
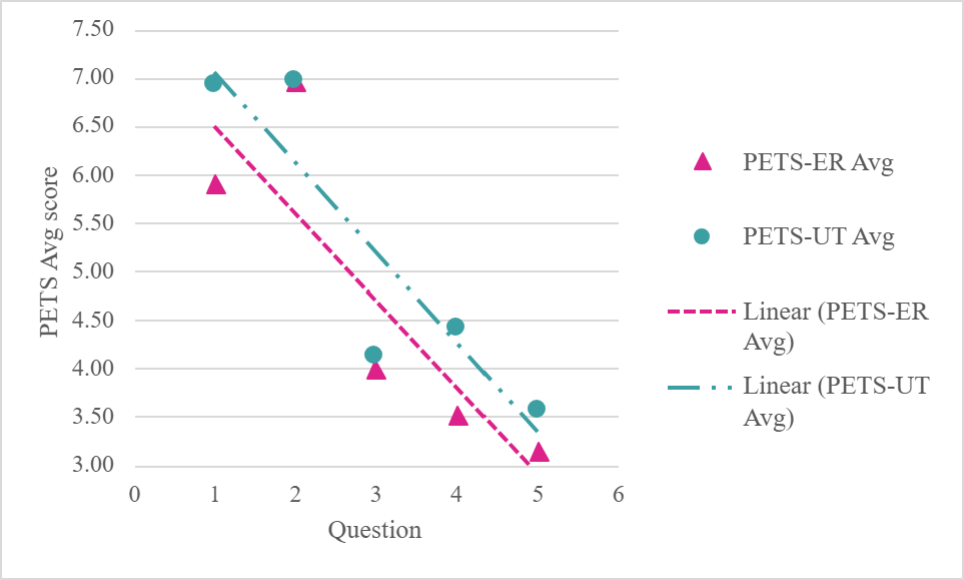
Average Perceived Empathy of Technology Scale (PETS) across chatbot by question. Avg: average; ER: Emotional Responsiveness; UT: Understanding and Trust.

PETS scores varied at the question and chatbot level (Table 2). Questions 1 and 2 are associated with the highest perceived empathy, and Question 5 demonstrates the lowest empathy scores (Figure 4).

**Figure 5.**
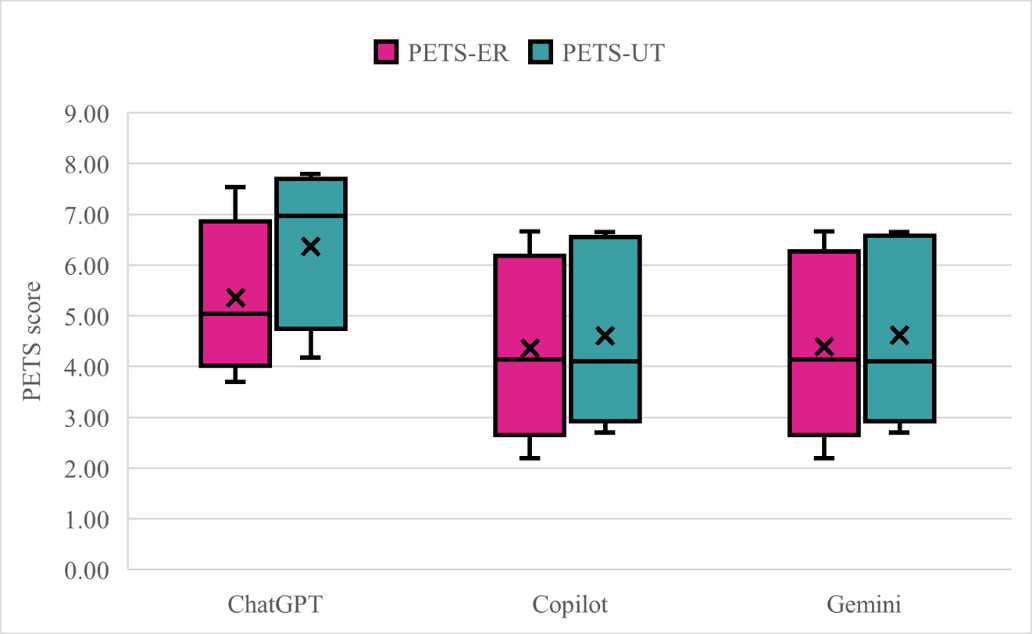
Box and whisker plots showing Perceived Empathy of Technology Scale-Emotional Responsiveness (PETS-ER) and Perceived Empathy of Technology Scale-Understanding and Trust (PETS-UT) scores across chatbots, illustrating limited overall perceived empathy compared to understandability.

When empathy scores were summarized by chatbot, descriptive differences in average perceived empathy were observed across models (Figure 5). ChatGPT responses demonstrated higher average empathy scores compared to Copilot and Gemini, which demonstrated lower averages. Across all models, PETS-UT scores exceeded PETS-ER scores, suggesting stronger cognitive empathy and perspective taking rather than emotional expression.

ChatGPT showed the largest UT-ER gap (mean 6.36, SD 1.83 vs 5.35, SD 1.85), indicating moderate-to-high levels of understanding, but only moderate emotional connection. Copilot and Gemini had similar low-moderate ER and UT averages, with limited perceived empathy overall. Variability patterns further distinguish AI models (Figure 5). ChatGPT produced the widest variation in empathy scores. In contrast, Gemini and Copilot showed slightly more predictable patterns of empathy.

### Readability (FRE, FKGL, and Word Count)

All outputs were written at an eighth-grade reading level or higher (range 8.2‐14.6, mean 11.82, SD 1.44, equivalent to high school senior level). The average FRE score across all AI chatbot responses was 38.60, SD 9.00, indicating difficult readability overall. Gemini tended to produce the longest but most readable responses. Copilot consistently produced shorter responses associated with lower average readability. ChatGPT produced responses with the most variability in readability and word count. Question 5 responses were the least readable (mean grade level 12.76, SD 0.71), while question 2 responses were the most readable (mean grade level 9.9, SD 1.01).

## Discussion

### Quality

ChatGPT responses were associated with higher average quality scores and moderate variability across multiple questions. Gemini responses demonstrated moderate quality scores with greater consistency, while Copilot responses exhibited greater variability and the lowest quality scores, even producing a factually incorrect answer regarding genetic mutations and the associated risk of developing melanoma (see Copilot 4_1 in Multimedia Appendix 4). In health communication, information quality standardization is critical because it allows for less confusion and increases patient-provider trust [12].

Quality is not only model dependent but also question reliant. Questions 1 and 2 received the highest quality evaluations, whereas Questions 4 and 5 produced lower quality scores. This pattern suggests that chatbots tend to provide more consistent responses to straightforward or well-established topics. In contrast, questions regarding more complex science with sparse and/or unclear literature, such as recurrence risk, long-term treatment effects, and genetic implications, produced less reliable responses, often without communicating their limitations. These complex topics often represent areas of greatest concern for AYA patients with melanoma, which underscores the importance of cautious interpretation of chatbot-generated information.

The review of lower-quality outputs revealed common pitfalls. Responses with low-quality scores were often oversimplified, incomplete, or presented in bullet-point style responses, with insufficient detail to address the question. The lack of actionable information was most apparent in discussions of treatment, side effects, and hormone changes. For example, when a patient asked how treatment would affect hormones, responses accurately identified potential consequences, such as adrenal insufficiency, but failed to describe symptoms. From a patient perspective, clinically beneficial communication translates these answers into recognizable manifestations at home (ie, fatigue, dizziness, or other changes). Accuracy was further undermined by the lack of transparent source citation and inconsistent use of safeguards, such as disclaimers. Instead, statements were presented as definitive, risking patient confusion when conflicting with other resources or provider guidance.

Conversely, higher-quality responses demonstrated more complete and organized content. They cited credible resources, gave disclaimers, and emphasized the importance of deferring to the health care team for individualized guidance. These responses were detailed and well-tailored to the questions, without an overwhelming word count, which improved readability and digestion of information. They had clear directions to access specific resources for further support and actionable suggestions for the patient. These qualities serve as a reference standard for future versions of AI-generated health information.

Although the overall quality scores across all chatbot responses were moderate to high, persistent gaps in sourcing and uncertainty disclosures raise serious patient safety concerns. For AYA patients with melanoma undergoing treatment, inconsistent quality can undermine trust and lead to confusion at a vulnerable stage of care. To be reliable in clinical contexts, AI systems must deliver outputs that are not only accurate and detailed but also verifiable, transparent, and responsibly framed.

### Empathy

Across the 3 chatbot platforms, a central pattern emerged: systems struggled to balance clinical accuracy with emotional support. ChatGPT comparatively showed the highest empathetic capacity, while Gemini and Copilot tended to be neutral and lacking emotion. The qualitative review of responses identified a set of best practices that distinguished qualities of higher-empathy outputs from weak ones (Textbox 2). Detailed examples supporting these best practices are provided in Multimedia Appendix 5.

Textbox 2. **Key best practices for artificial intelligence (AI) chatbot responses to express empathy.**

**Best practices (Perceived Empathy of Technology Scale [PETS] scores)**

- Open with empathy and understanding
- Asks follow-up and clarification questions
- Symptoms and side effects in plain language
- Medical disclaimers and boundaries
- Cites credible resources
- Specific actionable coping strategies
- Adolescents and young adults–specific
- Demonstrates interest in the patient
- Provides empathy in all contexts

**Common pitfalls (↓ PETS scores)**

- Robotic or purely clinical tone
- One-way information delivery
- Lists of side effects or clinical jargon
- Overstep scope
- Lack citations or specific resources
- Generic or overwhelming advice
- Adult-centric or generic framing
- Lack of interest or personalization
- Overlooks unspoken emotional needs

AYA patients with melanoma have unique needs compared to adults; however, chatbots often default to general or even pediatric framing [13]. When AYA patient-specific needs were addressed, it was often with a surface-level list rather than a meaningful explanation. Systems were better equipped to name emotions rather than express them, as demonstrated by higher UT than ER scores, and empathy was also prompt-dependent. Chatbot responses generally failed to infer patient distress if not explicitly stated (as seen in questions 3-5), unlike a human clinician who can use nonverbal cues and proactively address unspoken emotional needs even in the face of an analytical question. The chatbot lacks the emotional intelligence to deliver difficult news and interpret patient reactions. Responses receiving low PETS scores risk coming across as dismissive or cold, which may invalidate patient concerns and discourage them from seeking support or adhering to care recommendations in the future when they most need it.

Human providers naturally contextualize information based on patient age, life stage, current treatments, and psychosocial needs, a skill beyond a chatbot’s reach. Since our 5 questions represent domains of greatest emotional and informational needs of AYAs with melanoma, the irregularity of empathy suggests that current AI systems may not adequately support this unique patient population.

### Readability

**Figure 6.**
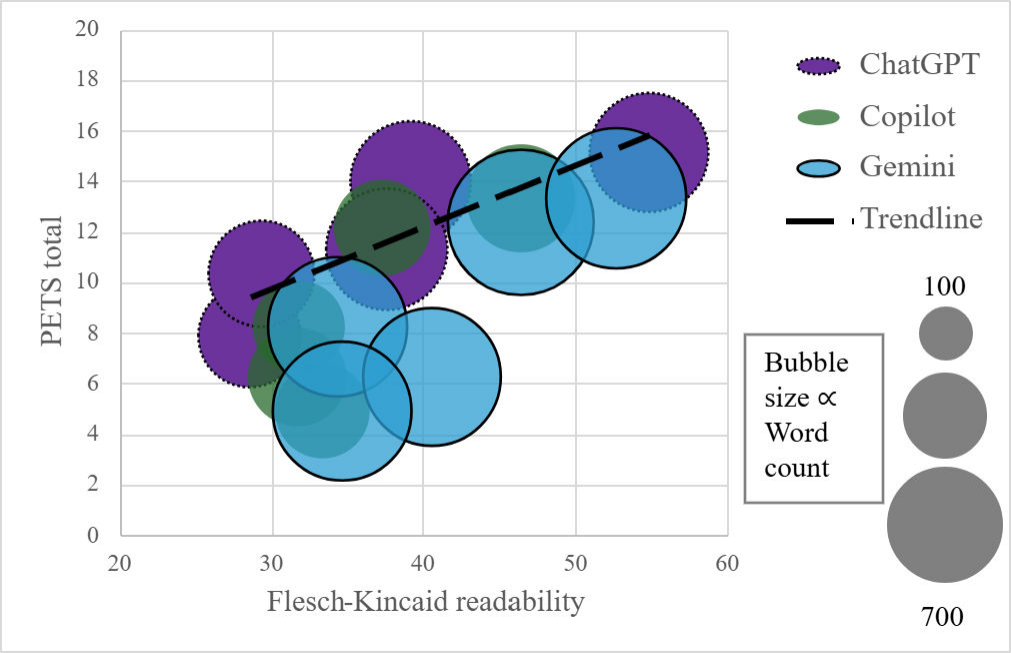
The relationship between Flesch-Kincaid readability and total Perceived Empathy of Technology (PETS) (empathy) scores across all chatbot responses. Each bubble represents a chatbot, with size proportional to overall word count. While word count shows little association with either readability or empathy, responses with higher readability tend to receive higher empathy scores.

Response length and readability varied across chatbots. Gemini responses were longer on average with higher readability scores, whereas ChatGPT responses demonstrated wider variability, and Copilot responses were shorter with lower average readability scores. Responses that were easier to read tended to be perceived as higher quality (GQS/DISCERN) and more empathetic (PETS) as seen in Figure 6.

Readability analysis revealed that all chatbot responses exceeded the US average reading level (7th-8th grade), with outputs averaging at the 11th-12th grade level. This creates a barrier for accessibility. These findings echo prior research that health care documents are often written at a level too high for general populations, reducing inclusivity and comprehension [14]. Unlike clinicians, who are trained to use strategies such as follow-up questions and the “teach-back” method, chatbots do not check understanding unless prompted. This may highlight a key limitation of AI chatbots for medical communication. They draw primarily from online sources, which are often already written at inflated reading levels, and thus lead to reproduced complexity by AI outputs [15]. Clinicians, by contrast, are trained to adapt explanations to patient literacy, developmental stage, and clinical context.

Readability is particularly important for AYAs, whose health literacy may be affected by education disruption, treatment-related cognitive impairment, and emotional distress. Cancer-related cognitive impairment affects approximately 25% to 28% of AYA survivors and may affect attention, memory, and processing speed, increasing the cognitive burden of complex medical information [16]. The National Comprehensive Cancer Network Guidelines for AYA Oncology emphasize that information should be delivered in an age-appropriate and developmentally appropriate manner, particularly in the context of disrupted education and psychosocial stressors [17]. While no evidence-based guidelines specify an optimal reading level for AYA oncology populations, the commonly cited 8th-grade target is extrapolated from general health literacy recommendations and represents a pragmatic benchmark rather than an AYA-specific threshold.

AI may therefore generate content that is technically accurate but inaccessible, potentially leading to confusion or disengagement. For proper implementation, chatbots should aim to target their output responses of health care information to an 8th-grade level and incorporate interactive strategies that mimic provider feedback loops. Without these adjustments, the use of AI to answer the needs of AYA patients with melanoma remains limited.

### Patient Safety and Clinical Implications

Although AI chatbots may provide AYA patients with adequate education, support, and resources regarding their diagnosis, they cannot replace clinician input and should be used cautiously. The potential dangers associated with patients using AI in health care are multifaceted, with key risks including misinformation, unsafe recommendations, lack of understanding of the questions being asked, and insensitivity. Given that AYAs are the primary users of AI compared to other age groups, they may be particularly vulnerable to these risks due to their vulnerability to algorithmic bias and variable access to clinical guidance [18]. These risks are inferred from content characteristics of chatbot responses and were not measured as patient outcomes in this study.

AI use in health care has been shown to spread misinformation [19]. In our study, we found that chatbot explanations regarding the causes of metastatic melanoma and recommendations for next steps may include inaccurate or misleading information. Responses across all 3 platforms often focused on sun avoidance and using sun protection factor to prevent melanoma recurrence or secondary malignancy. Although this advice may be relevant for certain patient populations, it is not applicable to all patients, particularly for individuals with skin of color. As a result, responses lack inclusivity for diverse patient communities. Additionally, some chatbot responses encouraged AYA patients to explore clinical trials available for their conditions. While well-intended, this response is particularly unsafe to AYA patients, as it may increase the risk of false hope or confusion if patients are encouraged to consider trials that are not medically appropriate. Some responses even offered to interpret lab and genetic testing results. While AI chatbots have the potential to explain pathology reports and simplify medical jargon to patients, their interpretations are not flawless and should be used carefully with clinician oversight [20]. Encouraging vulnerable patients to input their private health information to AI systems may expose them to breaches of data privacy and security, potentially leading to unauthorized access or misuse of sensitive medical information [21].

### Limitations

This study evaluated a limited number of single-turn responses (5 question and 75 single-turn responses), which may not fully capture chatbot performance. AI chatbots can refine responses through follow-up prompts, so the single-turn design does not reflect extended, multiturn conversations. This limitation may influence the perceived quality, empathy, and readability of chatbot responses. Studies testing multiturn chats may be beneficial in the future. In addition, chatbot responses are sensitive to prompt phrasing and contextual detail, and the use of subjective scoring instruments introduces potential variability in interpretation. Each response was independently evaluated by 2 reviewers per domain, with scores averaged to reduce individual rater bias. For transparency, we have included all outputs in MultimediaAppendices 4, 6  7 as well as an example of the grading process with score justifications in Multimedia Appendix 8. Formal interrater reliability statistics were not calculated, which limits quantitative assessment of scoring consistency. Although duplicate independent scoring with averaging was used to reduce subjectivity, future studies with larger and cross-disciplinary reviewer pools should incorporate formal reliability testing to further strengthen methodological rigor. Additionally, the separation of reviewer roles by domain may introduce systematic differences in scoring interpretation.

### Future Directions

Moving forward, we aim to use responses from this dataset to develop resources within melanoma oncology clinics that answer these questions and provide higher-quality and safer responses for AYA patients with melanoma. By providing age-appropriate education and support tools, we hope to improve the treatment experience and the overall well-being for AYAs navigating melanoma care. This will help expand the currently limited resources available for AYAs currently receiving treatment and hopefully bring more attention to the unaddressed survivorship needs of patients. Future clinic-integrated resources would be developed with clinician oversight and human evaluation to ensure accuracy, safety, and appropriateness for patient use, rather than relying on autonomous AI fine-tuning. If this study were to be replicated or expanded upon, it would be helpful to incorporate intraclass correlation coefficient or Cohen κ into the data analysis to correct for discrepancies in the grading system. Additionally, this study establishes a patient-centered framework that can be applied to future research evaluating AI chatbot communication around emerging and experimental melanoma therapies, including novel immunotherapies and mRNA-based cancer vaccines. Our findings suggest that chatbot performance declines in areas with limited or evolving scientific literature, indicating that AI responses on new treatments or clinical trials may be particularly vulnerable to misinformation or oversimplification. Future studies should specifically assess this phenomenon to generate realistic expectations for AYAs navigating the rapidly evolving melanoma therapeutic landscape.

### Conclusion

This study provides an evaluation of the utility of AI to answer the most pressing needs of AYA patients with melanoma currently receiving treatment. Overall, chatbot responses were often informative but demonstrated persistent gaps in quality, empathy, and readability. Patient safety may be at risk due to the inability of AI systems to integrate patient context, emotional nuance, and conversational reciprocity into answers. Question wording, emotional phrasing, and clarity were predictive of the responses received. When questions addressed topics covered by multiple publications, AI responses were more consistent, with higher quality. However, when questions addressed controversial or rapidly changing topics, AI responses were often vague. Quality, empathy, and readability were all interconnected, as higher-rated responses incorporated clear and concise wording, showed interest in the patient’s medical status, provided tangible resources and references, and recognized the limits of AI, encouraging further conversation with medical providers. This research should be replicated and can provide a reference standard for future AI chatbot responses for AYA patients with melanoma.

## Supplementary material

10.2196/84234 Multimedia Appendix 1 Global Quality Score (GQS), DISCERN, and Perceived Empathy of Technology Scale (PETS) scoring scales utilized for this study.

10.2196/84234 Multimedia Appendix 2 Chatbot response scores for each input.

10.2196/84234 Multimedia Appendix 3 Calculated standard deviations of response scores.

10.2196/84234 Multimedia Appendix 4 All Microsoft Copilot outputs.

10.2196/84234 Multimedia Appendix 5 Expanded best practices table.

10.2196/84234 Multimedia Appendix 6 All Google Gemini outputs.

10.2196/84234 Multimedia Appendix 7 All ChatGPT outputs.

10.2196/84234 Multimedia Appendix 8 Example of response score breakdown.

## Abbreviations

AI: artificial intelligence
AYA: adolescents and young adults
ER: Emotional Responsiveness
FKGL: Flesch-Kincaid Grade Level
FRE: Flesch Reading Ease
GQS: Global Quality Score
NA-SB: Needs Assessment and Service Bridge
PETS: Perceived Empathy of Technology Scale
UT: Understanding and Trust
