# Supplementary material for: Quality, Empathy, and Readability of AI Chatbot Responses to the Survivorship Needs of Adolescents and Young Adults With Melanoma: Evaluation Study
Source: JMIR Cancer. 2026 Mar 26;12:e84234. doi: 10.2196/84234 (PMC13020680; doi:10.2196/84234)
Supplement: Multimedia Appendix 1 [file cancer-v12-e84234-s001.docx]

**MA 1**

Table S1. GQS Scoring Scale

| Score | Description |
| --- | --- |
| 1 | Poor quality; poor flow, most information missing, not useful for patient education |
| 2 | Generally poor; some information present but of limited use |
| 3 | Moderate quality; some important information is adequately discussed |
| 4 | Good quality; most relevant information covered, useful for patient education |
| 5 | Excellent quality; comprehensive, clear, and highly useful for patient education |

Table S2. DISCERN Scoring Scale. The highlighted rows are the questions used in our analysis.

| Question | Assessment Focus |
| --- | --- |
| 1 | Are the aims clear? |
| 2 | Does it achieve its aims? |
| 3 | Is it relevant? |
| 4 | Is it clear what sources of information were used to compile the publication (other than the author or producer)? |
| 5 | Is it clear when the information used or reported in the publication was produced? |
| 6 | Is it balanced and unbiased? |
| 7 | Does it provide details of additional sources of support and information? |
| 8 | Does it refer to areas of uncertainty? |
| 9 | Does it describe how each treatment works? |
| 10 | Does it describe the benefits of each treatment? |
| 11 | Does it describe the risks of each treatment? |
| 12 | Does it describe what would happen if no treatment is used? |
| 13 | Does it describe how the treatment choices affect overall quality of life? |
| 14 | Is it clear that there may be more than one possible treatment choice? |
| 15 | Does it provide support for shared decision-making? |
| 16 | Based on the answers to all of the above, rate the overall quality of the publication as a source of information about treatment choices. |

Table S3. PETS scoring scale

| Subscale | Item | Label |
| --- | --- | --- |
| ER | E1 | The system considered my mental state |
| ER | E2 | The system seemed emotionally intelligent |
| ER | E3 | The system expressed emotions |
| ER | E4 | The system sympathized with me |
| ER | E5 | The system showed interest in me |
| ER | E6 | The system supported me in coping with an emotional situation |
| UT | U1 | The system understood my goals |
| UT | U2 | The system understood my needs |
| UT | U3 | I trusted the system |
| UT | U4 | The system understood my intentions |
