# Supplementary material for: Quality, Empathy, and Readability of AI Chatbot Responses to the Survivorship Needs of Adolescents and Young Adults With Melanoma: Evaluation Study
Source: JMIR Cancer. 2026 Mar 26;12:e84234. doi: 10.2196/84234 (PMC13020680; doi:10.2196/84234)
Supplement: Multimedia Appendix 2 [file cancer-v12-e84234-s002.docx]

MA 2. Chatbot response scores for each input

| **Chatbot** | **Question #** | **GQS (1-5)** | **DISC-16** | **PETS ER** | **PETS UT** | **Flesch Kincaid Reading Ease** | **Flesh-Kincaid grade level** | **Word count** |
| --- | --- | --- | --- | --- | --- | --- | --- | --- |
| ChatGPT | 1_1 | 4.5 | 3.3125 | 5.75 | 7.5 | 39.4 | 12.6 | 457 |
| ChatGPT | 1_2 | 5 | 3.5 | 6.73 | 8.375 | 46.5 | 9.7 | 549 |
| ChatGPT | 1_3 | 4.5 | 3.125 | 5.916 | 7.375 | 40 | 13.2 | 552 |
| ChatGPT | 1_4 | 4.5 | 3.75 | 7.583 | 8.75 | 36.2 | 14 | 635 |
| ChatGPT | 1_5 | 4 | 3 | 4.916 | 7 | 33.9 | 13.6 | 501 |
| ChatGPT | 2_1 | 4.5 | 3.1875 | 7.833333333 | 8.75 | 55 | 9.1 | 410 |
| ChatGPT | 2_2 | 4.5 | 3.3125 | 6.833333333 | 6.5 | 50.5 | 9.6 | 532 |
| ChatGPT | 2_3 | 4.5 | 3.3125 | 8.166666667 | 7.5 | 54.4 | 8.8 | 493 |
| ChatGPT | 2_4 | 5 | 3.5 | 7.666666667 | 7.75 | 60.1 | 8.2 | 554 |
| ChatGPT | 2_5 | 4.5 | 3.4375 | 7.166666667 | 7.5 | 54.2 | 9.8 | 623 |
| ChatGPT | 3_1 | 4 | 3 | 3.5 | 3.75 | 23.4 | 12.4 | 381 |
| ChatGPT | 3_2 | 4 | 3 | 3.666666667 | 4.5 | 29.2 | 11.7 | 408 |
| ChatGPT | 3_3 | 4 | 3 | 4 | 4.375 | 32.3 | 11.3 | 404 |
| ChatGPT | 3_4 | 4 | 3 | 5.5 | 5.25 | 24 | 12.3 | 442 |
| ChatGPT | 3_5 | 4 | 3 | 1.833333333 | 3 | 34 | 12.2 | 311 |
| ChatGPT | 4_1 | 4.5 | 2.875 | 5 | 7.333333333 | 36.2 | 10.9 | 541 |
| ChatGPT | 4_2 | 4.5 | 2.9375 | 4.333333333 | 7.25 | 38.7 | 10.7 | 500 |
| ChatGPT | 4_3 | 4.5 | 2.875 | 4.333333333 | 7.25 | 37.6 | 11.1 | 599 |
| ChatGPT | 4_4 | 4.5 | 3.25 | 4 | 6 | 39 | 10.4 | 514 |
| ChatGPT | 4_5 | 4.5 | 2.75 | 4 | 7 | 36.6 | 11.1 | 572 |
| ChatGPT | 5_1 | 4 | 2.9375 | 6.333333 | 5.75 | 25.2 | 13 | 414 |
| ChatGPT | 5_2 | 4.5 | 2.875 | 6.333333333 | 6.5 | 39 | 11.3 | 428 |
| ChatGPT | 5_3 | 5 | 3.75 | 4 | 5 | 33.9 | 12.5 | 516 |
| ChatGPT | 5_4 | 4.5 | 3.875 | 7.166666667 | 7.75 | 26.5 | 13.2 | 596 |
| ChatGPT | 5_5 | 4 | 2.875 | 1.333333333 | 1.5 | 22.2 | 13.9 | 124 |
| Copilot | 1_1 | 4 | 2.625 | 5.583 | 6.375 | 41.8 | 11.9 | 412 |
| Copilot | 1_2 | 4 | 2.8125 | 5.25 | 6.375 | 37.6 | 12.1 | 339 |
| Copilot | 1_3 | 4 | 2.8125 | 7.083 | 7.25 | 38.8 | 11.1 | 324 |
| Copilot | 1_4 | 4 | 2.6875 | 5.33 | 6 | 27.6 | 13 | 269 |
| Copilot | 1_5 | 4 | 2.8125 | 5.25 | 6.25 | 40.8 | 11 | 380 |
| Copilot | 2_1 | 5 | 3.3125 | 6.5 | 6.75 | 50.1 | 10 | 424 |
| Copilot | 2_2 | 4.5 | 3.3125 | 6.333333333 | 6.5 | 45.3 | 11 | 401 |
| Copilot | 2_3 | 4 | 3 | 7.666666667 | 7.25 | 51 | 10.2 | 406 |
| Copilot | 2_4 | 4.5 | 3.0625 | 6.5 | 6.5 | 45.6 | 10.6 | 470 |
| Copilot | 2_5 | 4.5 | 3.0625 | 6.333333333 | 6.25 | 40.1 | 11.8 | 458 |
| Copilot | 3_1 | 4.5 | 2.8125 | 3.833333333 | 4.75 | 39.5 | 11 | 274 |
| Copilot | 3_2 | 4 | 2.75 | 4 | 3.75 | 34 | 12.3 | 263 |
| Copilot | 3_3 | 4 | 2.625 | 4.833333333 | 4.25 | 20.9 | 14.6 | 326 |
| Copilot | 3_4 | 3.5 | 2.6875 | 4 | 3.75 | 28.8 | 12.3 | 378 |
| Copilot | 3_5 | 4.5 | 2.875 | 4 | 4 | 35.9 | 11.7 | 335 |
| Copilot | 4_1 | 2.5 | 2.375 | 2.5 | 3 | 36.3 | 12.5 | 327 |
| Copilot | 4_2 | 3 | 2.4375 | 3.166666667 | 3 | 34.8 | 13.2 | 357 |
| Copilot | 4_3 | 3.5 | 2.625 | 3.166666667 | 3.75 | 24.9 | 14.3 | 372 |
| Copilot | 4_4 | 4 | 2.625 | 3.166666667 | 3 | 32.4 | 13.5 | 358 |
| Copilot | 4_5 | 4 | 2.6875 | 3.5 | 3 | 30.2 | 13.5 | 387 |
| Copilot | 5_1 | 3.5 | 2.5 | 1.5 | 2.5 | 22.5 | 13.9 | 275 |
| Copilot | 5_2 | 4 | 2.8125 | 2 | 2.75 | 37.7 | 12 | 350 |
| Copilot | 5_3 | 3.5 | 2.625 | 2.5 | 2.75 | 38.9 | 12.7 | 339 |
| Copilot | 5_4 | 4 | 3 | 2.5 | 2.75 | 34.9 | 12.3 | 365 |
| Copilot | 5_5 | 3.5 | 2.75 | 2.5 | 2.75 | 32.9 | 12.8 | 325 |
| Gemini | 1_1 | 4 | 3.125 | 5.75 | 5.25 | 47.6 | 10.8 | 787 |
| Gemini | 1_2 | 4 | 3 | 5 | 5.875 | 44.6 | 11.4 | 665 |
| Gemini | 1_3 | 4 | 3.3125 | 5.833 | 7.25 | 46.3 | 11.1 | 856 |
| Gemini | 1_4 | 4 | 3.0625 | 5.833 | 6.375 | 46.2 | 11.2 | 796 |
| Gemini | 1_5 | 4 | 2.9375 | 6.916 | 7.75 | 47.4 | 11 | 795 |
| Gemini | 2_1 | 4 | 2.9375 | 6.5 | 6.75 | 56.2 | 9.4 | 818 |
| Gemini | 2_2 | 4.5 | 3.25 | 6.333333333 | 6.5 | 44.1 | 11.5 | 721 |
| Gemini | 2_3 | 4 | 3 | 7.666666667 | 7.25 | 53.5 | 9.7 | 660 |
| Gemini | 2_4 | 4.5 | 3.375 | 6.5 | 6.5 | 55.4 | 9.5 | 743 |
| Gemini | 2_5 | 4 | 2.875 | 6.333333333 | 6.25 | 54.2 | 8.9 | 698 |
| Gemini | 3_1 | 4.5 | 3 | 3.833333333 | 4.75 | 31.4 | 14.2 | 643 |
| Gemini | 3_2 | 4.5 | 3 | 4 | 3.75 | 33.1 | 12.9 | 734 |
| Gemini | 3_3 | 4.5 | 3 | 4.833333333 | 4.25 | 35.5 | 12.9 | 688 |
| Gemini | 3_4 | 4.5 | 3 | 4 | 3.75 | 33.9 | 13 | 692 |
| Gemini | 3_5 | 4.5 | 3.25 | 4 | 4 | 38 | 12.6 | 832 |
| Gemini | 4_1 | 4 | 2.625 | 2.5 | 3 | 47.7 | 11.3 | 632 |
| Gemini | 4_2 | 4 | 2.875 | 3.166666667 | 3 | 40.4 | 12.3 | 698 |
| Gemini | 4_3 | 4 | 2.75 | 3.166666667 | 3.75 | 38.8 | 12.6 | 721 |
| Gemini | 4_4 | 4 | 2.8125 | 3.166666667 | 3 | 41 | 11.6 | 755 |
| Gemini | 4_5 | 4 | 3 | 3.5 | 3 | 35.1 | 13 | 709 |
| Gemini | 5_1 | 4 | 2.75 | 1.5 | 2.5 | 30.5 | 13.5 | 716 |
| Gemini | 5_2 | 4 | 2.75 | 2 | 2.75 | 34.3 | 12.7 | 676 |
| Gemini | 5_3 | 4.5 | 2.8125 | 2.5 | 2.75 | 32.9 | 13.1 | 686 |
| Gemini | 5_4 | 4 | 2.75 | 2.5 | 2.75 | 38 | 12.2 | 690 |
| Gemini | 5_5 | 4.5 | 2.75 | 2.5 | 2.75 | 37.7 | 12.2 | 800 |
