# Supplementary material for: Quality, Empathy, and Readability of AI Chatbot Responses to the Survivorship Needs of Adolescents and Young Adults With Melanoma: Evaluation Study
Source: JMIR Cancer. 2026 Mar 26;12:e84234. doi: 10.2196/84234 (PMC13020680; doi:10.2196/84234)
Supplement: Multimedia Appendix 3 [file cancer-v12-e84234-s003.docx]

MA 3. Standard deviations of response scores

| **Level** | **Item** | **GQS SD** | **DISC SD** | **PET-ER** | **PET-UT** | **FLRE SD** | **FKGL SD** | **Word Count SD** |
| --- | --- | --- | --- | --- | --- | --- | --- | --- |
| **By Chatbot (all questions)** | ChatGPT | 0.32 | 0.31 | 1.85 | 1.83 | 10.54 | 1.64 | 111.00 |
|  | Copilot | 0.53 | 0.24 | 1.74 | 1.72 | 7.71 | 1.23 | 54.90 |
|  | Gemini | 0.24 | 0.19 | 1.76 | 1.76 | 7.87 | 1.37 | 61.34 |
| **By Question per Chatbot** | ChatGPT 1 | 0.35 | 0.30 | 1.02 | 0.73 | 4.77 | 1.71 | 66.43 |
|  | ChatGPT 2 | 0.22 | 0.12 | 0.53 | 0.80 | 3.43 | 0.64 | 78.60 |
|  | ChatGPT 3 | 0.00 | 0.00 | 1.31 | 0.85 | 4.78 | 0.47 | 48.84 |
|  | ChatGPT4 | 0.00 | 0.19 | 0.41 | 0.55 | 1.24 | 0.30 | 40.76 |
|  | ChatGPT 5 | 0.42 | 0.50 | 2.38 | 2.35 | 6.90 | 0.97 | 178.75 |
|  | Copilot 1 | 0.00 | 0.09 | 0.79 | 0.47 | 5.68 | 0.82 | 54.70 |
|  | Copilot 2 | 0.35 | 0.15 | 0.57 | 0.38 | 4.37 | 0.72 | 30.91 |
|  | Copilot 3 | 0.42 | 0.10 | 0.40 | 0.42 | 7.22 | 1.35 | 47.10 |
|  | Copilot 4 | 0.65 | 0.14 | 0.37 | 0.34 | 4.47 | 0.65 | 22.22 |
|  | Copilot 5 | 0.27 | 0.19 | 0.45 | 0.11 | 6.52 | 0.72 | 34.47 |
|  | Gemini 1 | 0.00 | 0.14 | 0.68 | 1.01 | 1.20 | 0.22 | 69.88 |
|  | Gemini 2 | 0.27 | 0.21 | 0.57 | 0.38 | 4.91 | 0.99 | 58.94 |
|  | Gemini 3 | 0.00 | 0.11 | 0.40 | 0.42 | 2.51 | 0.62 | 71.51 |
|  | Gemini 4 | 0.00 | 0.14 | 0.37 | 0.34 | 4.59 | 0.70 | 45.08 |
|  | Gemini 5 | 0.27 | 0.03 | 0.45 | 0.11 | 3.20 | 0.57 | 50.51 |
| **By questions across all chatbots** | Q1 | 0.31 | 0.31 | 0.80 | 0.96 | 5.71 | 1.21 | 193.47 |
|  | Q2 | 0.32 | 0.19 | 0.67 | 0.69 | 5.42 | 1.01 | 139.59 |
|  | Q3 | 0.32 | 0.16 | 0.79 | 0.55 | 5.41 | 0.97 | 188.62 |
|  | Q4 | 0.58 | 0.22 | 0.70 | 1.90 | 5.17 | 1.21 | 149.08 |
|  | Q5 | 0.43 | 0.38 | 1.91 | 1.79 | 5.84 | 0.71 | 197.66 |
