# Supplementary material for: Quality, Empathy, and Readability of AI Chatbot Responses to the Survivorship Needs of Adolescents and Young Adults With Melanoma: Evaluation Study
Source: JMIR Cancer. 2026 Mar 26;12:e84234. doi: 10.2196/84234 (PMC13020680; doi:10.2196/84234)
Supplement: Multimedia Appendix 4 [file cancer-v12-e84234-s004.docx]

Microsoft Copilot

On Treatment Questions

**Question 1: I am an adolescent/young adult patient with metastatic melanoma and am currently receiving treatment. I want more information about what will happen when treatment finishes.**

Input 1:

Thanks for sharing that—it's incredibly brave of you to seek clarity while navigating something so intense. When treatment for metastatic melanoma finishes, especially for adolescent and young adult (AYA) patients, the journey doesn’t just stop. It shifts into a new phase focused on recovery, monitoring, and long-term well-being. Here's what you can expect:

🩺 Follow-Up Care & Monitoring

Regular Checkups: You'll likely have scheduled visits every few months initially, then less frequently over time. These include physical exams, skin checks, and imaging tests to monitor for recurrence or new melanomas.

Blood Work & Scans: Depending on your specific case, your care team may order blood tests or imaging (like CT or PET scans) to catch any signs of recurrence early.

Late Effects Surveillance: Doctors will watch for long-term side effects from treatment—these can include fatigue, hormonal changes, or immune-related issues depending on the therapies used.

🧠 Emotional & Psychosocial Support

Mental Health: Many AYA patients benefit from counseling or support groups. The emotional toll of cancer doesn’t end with treatment, and connecting with others who “get it” can be powerful.

Fertility & Life Planning: If you received therapies that might affect fertility, you may be referred to specialists to explore options like fertility preservation or family planning.

School, Work, and Identity: Reintegrating into daily life—whether that’s school, work, or social circles—can be challenging. AYA programs often offer tailored support to help you navigate this transition.

🧬 Clinical Trials & Ongoing Research

Access to Trials: Some AYA patients may be eligible for clinical trials focused on long-term outcomes or new therapies. These can offer additional options if recurrence occurs or if you're interested in contributing to future breakthroughs.

Genetic Counseling: If your melanoma had specific mutations (like BRAF), you might be offered genetic counseling to understand risks and implications for you and your family.

🌞 Lifestyle & Prevention

Sun Safety: You’ll be coached on how to protect your skin—sunscreen, clothing, and avoiding peak sun hours are key.

Healthy Habits: Nutrition, exercise, and sleep all play a role in recovery and reducing recurrence risk. Some centers offer survivorship programs to help build these habits.

You can explore more detailed follow-up guidelines from the AIM at Melanoma Foundation or dive into AYA-specific care strategies in this clinical review.

If you’d like, I can help you build a personalized checklist or prep questions for your next appointment. You're not alone in this—let’s make sure you feel equipped for what’s next.

Input 2:

Thanks for sharing that—it's incredibly brave of you to face this head-on and seek clarity. When treatment for metastatic melanoma finishes, especially for adolescents and young adults (AYA), the journey doesn’t just stop. It shifts into a new phase focused on recovery, surveillance, and long-term health. Here's what you can expect:

🩺 Follow-Up Care & Monitoring

After treatment, your care team will transition you into a follow-up plan tailored to your specific situation. This typically includes:

Regular physical exams every 3–6 months for the first few years, then annually

Skin checks to monitor for recurrence or new melanomas

Imaging tests (like CT or PET scans) if symptoms suggest recurrence

Blood work to track overall health and detect any abnormalities

This phase is crucial because the risk of recurrence is highest in the first five years.

🧠 Emotional & Mental Health Support

The end of treatment can bring mixed emotions—relief, anxiety, even uncertainty. Many AYA patients benefit from:

Counseling or therapy to process the experience

Support groups with others who’ve faced melanoma

AYA-specific programs that understand the unique challenges of young adulthood, like fertility concerns, career disruption, or identity shifts

🧬 Long-Term Side Effects & Health Risks

Your team will also watch for late effects of treatment, which can include:

Fatigue, neuropathy, or hormonal changes

Increased risk of developing a second melanoma

Immune-related side effects if you received immunotherapy

🧪 Clinical Trials & Research Opportunities

Some AYA patients choose to participate in clinical trials post-treatment, especially if they’re at high risk of recurrence or want access to emerging therapies. These trials may offer:

New immunotherapies or targeted treatments

Vaccines designed to prevent recurrence

Long-term monitoring and data collection to improve care for future patients

🧭 Life After Treatment: Rebuilding & Thriving

This phase is also about reclaiming your life:

Returning to school, work, or hobbies

Navigating relationships and social life

Planning for the future with your health in mind

Hospitals with AYA oncology programs often offer life planning resources, including fertility preservation, career counseling, and survivorship coaching.

Input 3:

Thank you for sharing that—it's incredibly brave of you to face this head-on and seek clarity. When treatment for metastatic melanoma finishes, especially for adolescent and young adult (AYA) patients, the journey doesn’t just stop. It shifts into a new phase: follow-up care, monitoring, and survivorship planning. Here's what you can expect:

🩺 What Happens After Treatment Ends

1. Regular Follow-Up Appointments

These are tailored to your stage and risk of recurrence.

Typically include physical exams, skin checks, and sometimes imaging tests.

Most frequent in the first 5 years post-treatment, when recurrence risk is highest.

2. Monitoring for Recurrence or New Melanomas

Your care team will watch for signs of the original melanoma returning or a new one developing.

You’ll be taught how to perform skin self-exams and recognize warning signs.

3. Managing Long-Term Side Effects

Treatments like immunotherapy or targeted therapy can have lingering effects.

Fatigue, hormonal changes, or immune-related issues may need ongoing management.

4. Emotional and Mental Health Support

Many AYA patients experience anxiety, depression, or PTSD after treatment.

Counseling, peer support groups, and survivorship programs can help you process and heal.

5. Fertility and Life Planning

If you received systemic therapies, fertility preservation or reproductive health may be part of your follow-up.

Discussions about education, career, and relationships are often integrated into survivorship care.

6. Lifestyle and Prevention Guidance

You’ll get advice on sun protection, nutrition, exercise, and reducing risk factors.

Staying healthy overall helps your body recover and lowers recurrence risk.

💡 Special Considerations for AYA Patients

Adolescents and young adults face unique challenges:

You might feel caught between pediatric and adult care systems.

Hospitals are increasingly developing AYA-specific programs to support your needs—from fertility to mental health to social reintegration.

If you’d like, I can help you build a personalized survivorship plan or explore local AYA melanoma support programs. You're not alone in this next chapter—there’s a whole team ready to walk it with you.

Input 4:

Thanks for sharing that—it's a big journey you're on, and wanting to understand what comes next is incredibly important. Here's a clear breakdown of what typically happens after treatment for metastatic melanoma, especially for adolescent and young adult (AYA) patients:

🩺 What Happens After Treatment Ends

1. Follow-Up Care

Regular checkups are essential to monitor for recurrence or new melanomas.

These may include:

Physical exams every 3–6 months for the first few years, then annually

Imaging tests (like CT or PET scans) if symptoms suggest recurrence

Skin exams at least once a year for life

2. Monitoring for Late Effects

Your care team will watch for long-term side effects from treatments like immunotherapy or targeted therapy.

These might include fatigue, hormonal changes, or immune-related issues.

3. Emotional and Mental Health Support

Many AYA patients face unique challenges like anxiety, depression, or identity shifts post-treatment.

Support groups, counseling, and survivorship programs tailored for young adults can be incredibly helpful.

4. Fertility and Life Planning

If you received treatments that could affect fertility, follow-up may include reproductive health consultations.

You might also explore options like fertility preservation or family planning support.

5. Risk of Recurrence or Second Melanoma

AYA patients have a higher risk of developing another melanoma, so lifelong vigilance is key.

Sun protection and regular dermatologic care are crucial.

🧠 Special Considerations for AYA Patients

Clinical Trials: Some trials are now expanding to include younger patients, though access can still be limited.

AYA Oncology Programs: Many hospitals now offer specialized programs that address the unique needs of 15–39-year-olds, including peer support, career counseling, and survivorship planning.

Input 5:

Thanks for sharing that—it's incredibly brave of you to face this head-on and seek clarity. When treatment for metastatic melanoma finishes, especially for adolescent and young adult (AYA) patients, the journey doesn’t just stop. It shifts into a new phase focused on recovery, monitoring, and long-term well-being. Here's what you can expect:

🩺 Follow-Up Care & Monitoring

After treatment, your care team will continue to monitor you closely. This phase is critical, especially in the first five years, when the risk of recurrence is highest.

Regular Checkups: These include physical exams and skin checks every 3–12 months depending on your risk level.

Imaging Tests: Scans may be done periodically or if symptoms suggest recurrence.

Blood Work: Sometimes used to monitor for signs of cancer or side effects from treatment.

🧠 Emotional & Psychological Support

AYA patients often face unique emotional challenges—balancing treatment with school, work, relationships, and identity.

Counseling or Therapy: Many find it helpful to talk with professionals who specialize in cancer survivorship.

Support Groups: Connecting with others your age who’ve been through similar experiences can be empowering.

🧬 Long-Term Side Effects & Health Risks

Some treatments, especially immunotherapies and targeted therapies, can have lingering effects.

Late Effects: These might include fatigue, hormonal changes, or organ-specific issues.

Second Cancers: There's a slightly increased risk of developing another melanoma, so lifelong skin monitoring is essential.

🧒 Age-Specific Needs for AYA Patients

Your age group has distinct needs that are often underrepresented in clinical trials. Hospitals are increasingly developing AYA oncology programs to address:

Fertility Preservation: If this wasn’t addressed before treatment, it’s worth discussing now.

Career & Education Planning: Survivorship programs can help you navigate life goals post-treatment.

Transition to Adult Care: If you started in pediatric oncology, you may shift to adult care providers.

🌱 Life After Treatment

This phase is about reclaiming your life and redefining your goals. Many survivors find new meaning, passions, or advocacy roles.

Survivorship Plans: Your care team may help you create a personalized roadmap for health maintenance.

Lifestyle Adjustments: Nutrition, exercise, and sun protection become key parts of your routine.

If you’d like, I can help you explore survivorship programs near you or walk through what a personalized follow-up plan might look like. You're not alone in this next chapter.

**Question 2: I am an adolescent/young adult patient with metastatic melanoma and am currently receiving treatment. In the last month I have become more worried about my cancer spreading. What do I need to know and what steps can I take to cope with this fear?**

Input 1:

I'm really glad you reached out—facing metastatic melanoma as a young adult is incredibly tough, and the fear of it spreading is a very real and valid concern. You're not alone in feeling this way, and there are ways to navigate the emotional weight of it while staying grounded in your treatment journey.

🧠 Understanding Your Fear

Fear of progression is common among cancer patients, especially when you're young and still building your life. It’s not just about physical health—it’s about hopes, relationships, plans, and identity. Here’s what’s important to know:

Fear doesn’t mean weakness: It’s a natural response to uncertainty and loss of control.

Your brain is trying to protect you: But sometimes it overdoes it, leading to anxiety, hypervigilance, or even avoidance.

You’re allowed to feel conflicted: You might feel hopeful one day and terrified the next. That’s okay.

🛠️ Coping Strategies That Actually Help

Here are some steps that have helped other young adults in similar situations:

1. Find Your People

Support groups for young adults with cancer can be life-changing. Organizations like CancerCare offer counseling and peer support tailored to your age group.

First Descents is a group that takes young adult cancer survivors on wilderness adventures—kayaking, climbing, surfing—to help reclaim joy and confidence.

2. Talk to a Therapist Who Gets It

Look for someone trained in oncology-related mental health. They can help you process fear, grief, and uncertainty without judgment.

Cognitive Behavioral Therapy (CBT) and Acceptance Commitment Therapy (ACT) are especially effective for cancer-related anxiety.

3. Create a “Control Zone”

Focus on what you can control: nutrition, sleep, movement, staying informed (but not overwhelmed).

Build routines that give you a sense of stability and predictability.

4. Ask Your Care Team About Monitoring

If your fear is tied to not knowing what’s going on inside your body, talk to your oncologist about how often scans or bloodwork are done.

Understanding your treatment plan and what signs to watch for can reduce the “what if” spiral.

5. Express Yourself

Journaling, art, music, or even just talking to someone can help externalize your fear so it doesn’t live entirely in your head.

Some patients find meaning in sharing their story—like Linda Curtis, who was diagnosed at 20 and has faced multiple recurrences with resilience and openness.

6. Stay Curious About Joy

It’s okay to laugh, dream, and plan—even with cancer. Humor and joy aren’t denial; they’re survival tools.

People like Lauren Race, diagnosed at 24, found strength in humor and community while educating others about melanoma.

Input 2:

I'm really glad you reached out—facing metastatic melanoma as a young adult is incredibly tough, and the fear of it spreading is a very real and valid concern. You're not alone in feeling this way, and there are ways to manage the emotional weight while staying informed and empowered.

🧠 Understanding the Fear of Progression

Fear of cancer spreading—often called fear of recurrence or progression—is common among patients with metastatic disease. It can be triggered by:

Changes in symptoms or side effects

Upcoming scans or test results

Stories of others’ experiences

Uncertainty about the future

This fear doesn’t mean you’re not coping well—it means you care deeply about your life and your future.

🛠️ Steps to Cope with the Fear

Here are some strategies that have helped other young adults in similar situations:

1. Build a Support System

Connect with others who understand your experience. Organizations like CancerCare offer support groups specifically for young adults with advanced cancer.

Consider joining programs like First Descents, which provide outdoor adventures for young adult cancer survivors—these can be transformative.

2. Talk to a Mental Health Professional

A counselor or psychologist trained in oncology can help you process your fears and develop coping strategies.

Cognitive Behavioral Therapy (CBT) and mindfulness-based approaches have shown strong results in reducing anxiety related to illness.

3. Stay Informed—but Set Boundaries

Understanding your treatment plan and prognosis can help reduce uncertainty.

However, avoid doom-scrolling or obsessively researching—set limits on how much time you spend reading about cancer online.

4. Practice Mindfulness and Grounding

Techniques like meditation, breathing exercises, or journaling can help you stay present and reduce spiraling thoughts.

Apps like Headspace or Insight Timer offer free guided meditations tailored to anxiety and illness.

5. Create Meaning and Joy

Pursue activities that bring you joy, even in small doses—music, art, nature, or time with loved ones.

Some young adults find purpose in advocacy, storytelling, or helping others navigate their own cancer journeys3.

💬 Real Stories, Real Strength

People like Jonah and Lauren, both diagnosed with metastatic melanoma in their 20s, found strength through community, counseling, and a refusal to let cancer define their joy3. Their stories show that while fear is part of the journey, so is resilience.

If you ever want to talk through specific worries, explore coping tools, or just vent—I’m here. You don’t have to carry this alone.

Input 3:

I'm really glad you reached out—facing metastatic melanoma as a young adult is incredibly tough, and the fear of it spreading is a very real and valid concern. You're not alone in feeling this way, and there are steps you can take to help manage that fear while staying grounded in your treatment journey.

🧠 Understanding Your Fear

Fear of progression is common among cancer patients, especially when you're young and navigating so many life transitions at once. Here’s what helps to know:

Uncertainty is part of the process: Even with the best treatments, outcomes can be unpredictable. That doesn’t mean you’re powerless.

Fear doesn’t mean weakness: It’s a sign that you care deeply about your life, your future, and the people around you.

Your medical team is your ally: They monitor your condition closely and can explain what signs to watch for, what’s normal, and what’s not.

🛠️ Coping Strategies That Actually Help

Here are some practical and emotional tools that other young adults with metastatic melanoma have found helpful:

1. Connect with Others Who Get It

Support groups for young adults with advanced cancer can be life-changing. Organizations like CancerCare offer free counseling and peer support.

First Descents offers outdoor adventures for young adult cancer survivors—many find it empowering and healing.

2. Build a Mental Health Toolkit

Therapy or counseling: Talking to a professional who understands cancer-related anxiety can help you process fear and build resilience.

Mindfulness and meditation: Apps like Headspace or Insight Timer offer guided practices that can calm racing thoughts.

Journaling: Writing down your fears can help you externalize them and gain perspective.

3. Stay Informed—but Not Overwhelmed

Ask your oncologist about your current status and what signs of progression to look for. Knowledge can reduce uncertainty.

Avoid doomscrolling or reading worst-case scenarios online. Stick to trusted sources and ask your care team for guidance.

4. Create Meaning and Joy

Many young adults find strength in focusing on what they can control—relationships, creativity, advocacy, or simply finding joy in small moments.

People like Linda Curtis, diagnosed at 20, have shared how they coped through multiple recurrences by staying connected to purpose and hope.

💬 What You’re Feeling Is Valid

Lauren Race, diagnosed at 24, found that humor, community, and a second opinion helped her cope with the emotional toll of metastatic melanoma. Her story is a reminder that even in the darkest moments, light can break through.

Input 4:

I'm really glad you reached out—facing metastatic melanoma as a young adult is incredibly tough, and it's completely valid to feel anxious about the possibility of it spreading. You're not alone in this, and there are ways to manage both the medical uncertainty and the emotional weight that comes with it.

🧠 Understanding the Fear of Progression

Fear of cancer spreading—often called fear of recurrence or progression—is common among patients with metastatic disease, especially younger adults. It can be triggered by:

Changes in symptoms or side effects

Upcoming scans or test results

Stories from others or online information

Feeling physically vulnerable or emotionally isolated

This fear doesn’t mean you’re weak—it means you’re human, and your mind is trying to protect you from uncertainty.

🩺 What You Should Know Medically

Monitoring is key: Regular imaging and blood tests help your care team track any changes early. Staying informed about your treatment plan can give you a sense of control.

Treatment advances: Immunotherapy, targeted therapy, and clinical trials have significantly improved outcomes for metastatic melanoma—even in younger patients.

Second opinions matter: If you're unsure about your current plan or feel dismissed, seeking a second opinion from a melanoma specialist can be empowering.

🛠️ Coping Strategies That Actually Help

Here are some practical and emotional tools that have helped other young adults in your shoes:

1. Connect with Others Who Get It

Support groups for young adults with advanced cancer (like those offered by CancerCare) can be life-changing.

Programs like First Descents offer adventure-based healing for young adult cancer survivors—kayaking, climbing, surfing, and more.

2. Therapy and Counseling

A counselor who specializes in oncology can help you process fear, grief, and uncertainty.

Cognitive Behavioral Therapy (CBT) is especially effective for managing anxiety related to illness.

3. Mindfulness and Grounding Techniques

Practices like meditation, breathwork, or yoga can help calm your nervous system.

Apps like Headspace or Insight Timer offer free guided sessions tailored to stress and illness.

4. Creative Expression

Journaling, music, art, or storytelling can help you externalize your fears and reclaim your narrative.

Some patients find meaning in sharing their journey publicly or privately—like Linda Curtis did.

5. Build a “Safety Net” Routine

Create a daily rhythm that includes something joyful, something grounding, and something social—even if it’s just texting a friend.

Limit doom-scrolling or excessive Googling about cancer. Curate your information sources.

💬 You’re More Than Your Diagnosis

You’re navigating something that most people your age can’t imagine. That takes courage. Whether it’s fear, anger, hope, or exhaustion—you’re allowed to feel it all. And you don’t have to do it alone.

If you’d like, I can help you find a local support group, suggest journaling prompts, or even help you write a letter to your future self. Just say the word.

Input 5:

I'm really glad you reached out—facing metastatic melanoma as a young adult is incredibly tough, and it's completely valid to feel anxious about the possibility of your cancer spreading. You're not alone in this, and there are ways to manage both the medical and emotional weight of what you're going through.

🧠 Understanding Your Fear

Fear of progression is common among cancer patients, especially when you're young and juggling treatment with hopes, relationships, and plans for the future. This fear can stem from:

Uncertainty about treatment outcomes

Physical symptoms that feel ambiguous or alarming

Stories of recurrence or progression in others

The emotional toll of living with a serious diagnosis

🩺 What You Should Know Medically

Monitoring and Communication: Stay in close contact with your oncology team. Ask about what signs to watch for and how often scans or check-ins are scheduled.

Treatment Advances: Metastatic melanoma treatment has evolved significantly. Immunotherapy, targeted therapy, and clinical trials offer more options than ever before.

Second Opinions: If you're feeling unsure, seeking a second opinion can offer reassurance or new perspectives.

🛠️ Coping Strategies That Help

Here are some steps that have helped other young adults in similar situations:

1. Connect with Support Networks

Organizations like CancerCare offer support groups specifically for young adults with advanced cancer.

You might also explore programs like First Descents, which provide outdoor adventures for young cancer survivors—these can be deeply healing.

2. Therapeutic Support

A counselor or therapist trained in oncology-related issues can help you process fear, grief, and uncertainty.

Cognitive Behavioral Therapy (CBT) and mindfulness-based approaches are especially effective for anxiety.

3. Creative Expression

Journaling, art, or music can help externalize your fears and give them shape—making them easier to confront.

Some patients find storytelling or blogging therapeutic, and it can also help others going through similar experiences.

4. Build a “Resilience Toolkit”

Include things like breathing exercises, grounding techniques, and affirmations.

Apps like Headspace or Calm offer guided meditations tailored to stress and anxiety.

5. Stay Informed—but Set Boundaries

Learn about your condition from trusted sources, but avoid doomscrolling or obsessively researching worst-case scenarios.

Ask your care team to help you interpret medical info so it doesn’t become overwhelming.

💬 Real Stories, Real Strength

People like Jonah and Linda have shared their journeys with metastatic melanoma, including the emotional rollercoaster and the ways they found strength in community, therapy, and even humor2. Their stories show that while fear is real, it doesn’t have to define your experience.

If you'd like, I can help you find a support group near you, or even draft a message to your care team about your concerns. You're doing something powerful just by naming your fear—and that’s the first step toward reclaiming control.

**Question 3: I am an adolescent/young adult patient with metastatic melanoma and am currently receiving treatment. How will my treatment affect long-term hormone changes?**

Input 1:

Thanks for sharing something so personal—I'm here to help you navigate this with clarity and care. The impact of metastatic melanoma treatment on long-term hormone changes in adolescents and young adults depends heavily on the type of therapy you're receiving. Here's a breakdown of what’s known:

🧬 Common Treatments & Hormonal Impacts

1. Immunotherapy (e.g., anti–PD-1, anti–CTLA-4)

Examples: Nivolumab, Pembrolizumab, Ipilimumab

Hormonal Effects:

Can trigger immune-related endocrine disorders, such as:

Thyroid dysfunction (hypothyroidism or hyperthyroidism)

Adrenal insufficiency

Pituitary inflammation (hypophysitis), which may affect multiple hormones

These effects may be permanent and require lifelong hormone replacement therapy

2. Targeted Therapy (e.g., BRAF/MEK inhibitors)

Examples: Dabrafenib + Trametinib

Hormonal Effects:

Less commonly associated with direct hormone disruption

May still affect fertility and pubertal development, especially in younger patients

3. Chemotherapy (less common now for melanoma)

Hormonal Effects:

Can impair gonadal function, leading to:

Reduced fertility

Delayed puberty or menstrual irregularities

These effects may be temporary or permanent depending on dosage and duration

🧠 Age-Specific Considerations

Since you're in adolescence or young adulthood, your endocrine system is still actively developing. That means:

Hormonal disruptions may affect growth, sexual development, and bone health

Long-term monitoring by an endocrinologist is often recommended

Fertility preservation options (like sperm or egg banking) may be discussed before treatment begins

🩺 What You Can Do

Ask your oncologist if you’re being monitored for endocrine side effects

Request baseline hormone testing if it hasn’t been done

Track symptoms like fatigue, weight changes, mood shifts, or menstrual irregularities—they can signal hormonal changes

Would you like help preparing questions for your next doctor visit or exploring fertility options? I’ve got your back.

Input 2:

Thanks for sharing something so personal—it's incredibly important to understand how treatment might affect your body long-term, especially during adolescence or young adulthood when hormones are already in flux.

🎯 Here’s what we know about potential hormone-related effects from metastatic melanoma treatments:

🧬 Immunotherapy (e.g., anti–PD-1 or anti–CTLA-4 agents)

Endocrine side effects are among the most common long-term issues.

You might experience:

Thyroid dysfunction (hypothyroidism or hyperthyroidism)

Adrenal insufficiency

Pituitary inflammation (hypophysitis), which can disrupt multiple hormone pathways

These changes can be permanent, requiring lifelong hormone replacement therapy.

💊 Targeted Therapy (e.g., BRAF/MEK inhibitors)

Less likely to cause direct hormone disruption compared to immunotherapy.

However, they can still affect fertility and pubertal development, especially in younger patients.

Long-term data is still evolving, but some survivors report fatigue, weight changes, and menstrual irregularities.

🧠 Age Matters

Adolescents and young adults are in a critical window for:

Growth and bone development

Sexual maturation

Mental health and cognitive development

Hormonal shifts caused by treatment can influence these areas, so endocrine monitoring is crucial during and after therapy.

🩺 What You Can Do

Ask your oncologist for regular hormone panels (thyroid, cortisol, sex hormones).

Consider seeing an endocrinologist who specializes in cancer survivorship.

Keep track of symptoms like fatigue, mood changes, irregular periods, or changes in libido—they can be subtle signs of hormone imbalance.

If you'd like, I can help you draft questions to ask your care team or explore survivorship plans tailored to young adults. You're not alone in navigating this—many others have walked this path and found ways to thrive.

Input 3:

Thanks for sharing something so personal—it's incredibly important to understand how treatment might affect your body long-term, especially when you're still developing.

🎯 Key Considerations for Hormonal Impact in Young Patients with Metastatic Melanoma:

Type of Treatment Matters

Immunotherapy (like anti–PD-1 or anti–CTLA-4 agents) is commonly used and generally doesn’t directly target hormone-producing glands, but rare autoimmune side effects can affect the thyroid, adrenal glands, or pituitary gland, leading to hormone imbalances.

Targeted therapies (like BRAF/MEK inhibitors) may have less direct hormonal impact but can still influence metabolism and fatigue levels, which are indirectly tied to hormonal health.

Chemotherapy (less commonly used for melanoma now) can affect reproductive hormones and fertility, especially in younger patients.

Endocrine Side Effects

Some patients develop hypothyroidism or adrenal insufficiency due to immune-related adverse events. These conditions may require lifelong hormone replacement therapy.

Pituitary inflammation (hypophysitis) can disrupt multiple hormone axes, including growth, reproductive, and stress hormones.

Puberty and Development

If you're still going through puberty or early adulthood, treatment-related hormone changes could potentially affect growth, sexual development, and bone health.

Monitoring by an endocrinologist is often recommended to track these changes and intervene early if needed.

Fertility and Reproductive Health

Some treatments may impact fertility, either temporarily or permanently. Fertility preservation options (like sperm or egg banking) are sometimes discussed before starting treatment.

Long-Term Survivorship

Encouragingly, many young patients who respond well to treatment can return to a normal life, but ongoing hormone monitoring is key to managing any lingering effects.

💡 What You Can Do

Ask your oncologist about regular endocrine evaluations.

Consider seeing a reproductive endocrinologist if fertility is a concern.

Keep a journal of symptoms like fatigue, mood changes, or weight fluctuations—they can be clues to hormone shifts.

If you'd like, I can help you draft questions to ask your care team or explore specific hormone-related conditions further. You're not alone in this—there’s a whole network of support and science behind you.

Input 4:

Thank you for sharing that—it's a deeply personal journey you're on, and it's important to understand how treatment might affect your body long-term, especially when it comes to hormones and development during adolescence or young adulthood.

🧬 Potential Hormonal Effects of Metastatic Melanoma Treatment

The impact on hormones depends heavily on the type of treatment you're receiving. Here's a breakdown of how common therapies might affect hormonal health:

1. Immunotherapy (e.g., anti–PD-1, anti–CTLA-4)

These are widely used in metastatic melanoma and have revolutionized outcomes.

Possible Hormonal Side Effects:

Endocrinopathies: These include inflammation or dysfunction of hormone-producing glands like the thyroid, adrenal glands, and pituitary.

Hypothyroidism or Hyperthyroidism: Can affect energy levels, metabolism, and mood.

Hypophysitis: Inflammation of the pituitary gland, which can disrupt multiple hormones (growth, sex, adrenal).

Diabetes (Type 1-like): Rare, but possible due to autoimmune attack on insulin-producing cells.

Long-Term Outlook:

Some hormonal changes may be permanent, requiring lifelong hormone replacement.

Others may be transient and resolve after treatment ends.

2. Targeted Therapy (e.g., BRAF/MEK inhibitors)

Used if your melanoma has specific genetic mutations.

Hormonal Impact:

Less commonly associated with direct hormonal disruption.

May indirectly affect metabolism or fertility, especially with long-term use.

3. Chemotherapy or Radiation (less common in melanoma)

If used, these can have broader systemic effects.

Hormonal Risks:

Gonadal toxicity: Potential impact on fertility and sex hormone production.

Growth and puberty: In adolescents, may delay or disrupt normal development.

🧠 Why This Matters for Adolescents and Young Adults

During adolescence and early adulthood, your body is still developing hormonally. Disruption to the endocrine system can affect:

Growth and bone density

Sexual development and fertility

Mood and cognitive function

Energy and metabolism

That’s why regular endocrine monitoring is crucial during and after treatment. Many oncology teams include endocrinologists to help manage these effects proactively.

💡 What You Can Do

Ask your care team about baseline and follow-up hormone testing.

Track symptoms like fatigue, weight changes, mood shifts, or irregular periods.

Discuss fertility preservation if that’s a concern—there are options even during treatment.

If you’d like, I can help you draft questions for your oncologist or endocrinologist to make sure you’re covering all the bases. You're not alone in navigating this—I'm here to help you make sense of it all.

Input 5:

Thanks for sharing something so personal—it's incredibly brave of you to ask this. When it comes to long-term hormone changes in adolescent and young adult patients with metastatic melanoma, the impact depends heavily on the type of treatment you're receiving. Here's a breakdown of what’s known and what to watch for:

🧬 Potential Hormonal Effects by Treatment Type

1. Immune Checkpoint Inhibitors (e.g., nivolumab, pembrolizumab, ipilimumab)

These therapies activate your immune system to fight melanoma.

Possible hormonal side effects:

Hypophysitis (inflammation of the pituitary gland) — can lead to deficiencies in hormones like cortisol, thyroid hormones, and sex hormones.

Thyroid dysfunction — hypothyroidism or hyperthyroidism may develop and persist.

Adrenal insufficiency — if the adrenal glands are affected, it can impact stress hormone production.

These changes can be long-term or permanent, requiring ongoing hormone replacement therapy.

2. Targeted Therapies (e.g., BRAF/MEK inhibitors like dabrafenib/trametinib)

These target specific mutations in melanoma cells.

Hormonal effects are less common, but:

Some patients report fatigue, which may be linked to subtle hormonal shifts.

Rarely, these drugs can affect gonadal function, especially in younger patients.

3. Chemotherapy or Radiation (less commonly used now)

These can affect gonadal function, potentially leading to:

Infertility

Delayed puberty or early menopause

Changes in testosterone or estrogen levels

🧠 Why This Matters for Adolescents and Young Adults

Your body is still developing hormonally, so disruptions can affect:

Growth and bone health

Mood and cognitive function

Sexual development and fertility

Endocrine side effects may not show up immediately, so long-term follow-up with an endocrinologist is crucial.

🩺 What You Can Do

Ask your oncologist for baseline hormone testing and regular monitoring.

If you notice symptoms like fatigue, weight changes, mood shifts, or irregular periods, speak up—they could be hormonal.

Consider fertility preservation options if that’s relevant to your treatment plan.

Would you like help preparing questions for your next appointment or exploring support resources for young adults with melanoma? You're not alone in this, and there are ways to protect your long-term health while fighting the disease.

**Question 4: I am an adolescent/young adult patient with metastatic melanoma and am currently receiving treatment. I want more information about how my genetics may or may not have impacted my diagnosis and treatment.**

Input 1:

Thanks for sharing that—it's incredibly important to understand how your genetics may play a role in both your diagnosis and treatment of metastatic melanoma. Here's a breakdown of what current research and clinical guidelines suggest:

🧬 Genetic Factors in Melanoma Diagnosis

Inherited Mutations: About 10% of melanoma cases are linked to inherited gene mutations. These mutations, such as in the CDKN2A, CDK4, or BAP1 genes, can significantly increase your lifetime risk of developing melanoma—sometimes up to 90%.

Family History: If you have close relatives with melanoma, especially if diagnosed at a young age, genetic testing might be recommended to assess your risk.

Physical Traits: Traits like fair skin, red or blonde hair, and having many atypical moles can also be genetically influenced and raise your risk.

💊 Genetic Influence on Treatment

BRAF Mutations: Around 40–60% of cutaneous melanomas carry a mutation in the BRAF gene. If your tumor has this mutation, targeted therapies like BRAF inhibitors (e.g., vemurafenib, dabrafenib) may be part of your treatment plan.

NRAS and KIT Mutations: These are less common but can also guide treatment decisions. For example, KIT mutations may respond to drugs like imatinib.

Tumor Microenvironment: Advances in genomic sequencing have revealed how your tumor’s genetic makeup interacts with its surrounding environment, influencing how well it responds to immunotherapy.

🧪 Should You Consider Genetic Testing?

Genetic testing is typically recommended if:

You have a strong family history of melanoma.

You were diagnosed at a young age.

You have multiple primary melanomas.

Even if you don’t have an inherited mutation, tumor genetic profiling (not the same as inherited genetic testing) is often done to guide treatment decisions, especially in metastatic cases.

If you're open to it, I can help you explore what kind of genetic testing might be appropriate or walk you through how to interpret your results. Would you like to dive deeper into any of these areas—like BRAF mutations or how immunotherapy is influenced by genetics?

Input 2:

You're asking a really important and insightful question—genetics can play a significant role in both the development and treatment of metastatic melanoma, especially for younger patients. Here's a breakdown of how your genetic makeup might be influencing your journey:

🧬 Genetic Factors in Melanoma Diagnosis

Inherited Mutations: About 10% of melanomas are linked to inherited gene mutations passed down through families. These mutations—such as in the CDKN2A, CDK4, or BAP1 genes—can significantly increase your lifetime risk of developing melanoma.

Physical Traits: Certain traits like fair skin, red or blonde hair, and a high number of atypical moles are genetically influenced and associated with higher melanoma risk.

Age and Type: Adolescents and young adults may be more likely to develop specific subtypes like acral or mucosal melanoma, which are less tied to UV exposure and more to genetic factors.

💊 Genetics and Treatment Decisions

Somatic Mutations: These are mutations that occur in the melanoma cells themselves (not inherited). The most common is a mutation in the BRAF gene, found in about 40–60% of melanomas. If your tumor has this mutation, targeted therapies like BRAF and MEK inhibitors may be part of your treatment.

Tumor Microenvironment: Your tumor’s genetic profile can affect how it interacts with your immune system. This influences how well treatments like immunotherapy work.

Genomic Testing: Next-generation sequencing and other advanced tests can help identify mutations in your tumor and guide personalized treatment strategies.

🧪 Should You Consider Genetic Testing?

For Inherited Risk: If you have a strong family history of melanoma or multiple primary melanomas, genetic testing might help assess your inherited risk.

For Treatment Planning: Tumor genetic profiling is often done automatically as part of your care to determine the best treatment options. If it hasn’t been discussed, you can ask your oncologist whether your tumor has been tested for mutations like BRAF, NRAS, or KIT.

If you're comfortable sharing more—like whether you've had any genetic testing already or what treatments you're receiving—I can help you interpret what it might mean for your care. You're navigating something incredibly complex, and it's brave of you to seek deeper understanding.

Input 3:

Thanks for sharing that—it's incredibly important to understand how your genetics might play a role in your melanoma journey. Here's a breakdown of what we know about genetic influences in adolescent and young adult (AYA) metastatic melanoma:

🧬 Genetic Factors in Melanoma Risk

Certain inherited traits and mutations can increase susceptibility to melanoma, especially in younger patients:

Familial Melanoma Genes: Mutations in genes like CDKN2A, CDK4, and MITF have been linked to familial melanoma. These mutations can impair cell cycle regulation, making it easier for abnormal cells to grow unchecked.

Congenital Nevi and Nevus Density: Having many moles (nevi), especially large ones, is associated with a significantly higher risk. Adolescents with more than 100 nevi have a 34-fold increased risk of melanoma.

Xeroderma Pigmentosum (XP): A rare genetic disorder that affects DNA repair. About 5% of individuals with XP develop melanoma by a median age of 19.

Fair Skin and Sun Sensitivity: Genetic traits like light skin, freckling, and a tendency to burn easily are nonmodifiable risk factors that increase vulnerability to UV-induced DNA damage.

🧪 Genetics and Treatment Response

Your genetic profile can also influence how your melanoma responds to treatment:

BRAF Mutations: Around 40–60% of melanomas (including in younger patients) carry mutations in the BRAF gene. If present, targeted therapies like BRAF inhibitors (e.g., vemurafenib, dabrafenib) may be used.

NRAS and KIT Mutations: These are less common but may guide alternative targeted therapies or clinical trial options.

Tumor Mutational Burden (TMB): A higher TMB may predict better response to immunotherapy, such as checkpoint inhibitors (e.g., nivolumab, pembrolizumab).

🧒 Unique Considerations for AYA Patients

Diagnostic Challenges: Melanoma in younger patients often presents atypically—sometimes as amelanotic (non-pigmented) lesions—which can delay diagnosis.

Treatment Protocols: While treatment often mirrors adult protocols, younger patients may be more likely to participate in clinical trials or receive tailored care based on genetic findings.

If you haven’t already, you might consider asking your care team about genetic testing or tumor sequencing. These tests can help identify mutations that may guide treatment decisions or reveal inherited risk factors that could be relevant for you or your family.

Would you like help preparing questions for your oncologist or exploring clinical trials that match your genetic profile?

Input 4:

Thanks for sharing that—your curiosity about the role of genetics in metastatic melanoma is incredibly important, especially as a young adult navigating treatment. Here's a breakdown of how genetics can influence both diagnosis and therapy decisions:

🧬 Genetic Factors in Melanoma Diagnosis

Inherited Mutations: About 10% of melanomas are linked to inherited gene mutations, such as those in the CDKN2A, CDK4, or BAP1 genes. These mutations can significantly increase your lifetime risk of developing melanoma—sometimes up to 90%.

Family History: If close relatives have had melanoma, especially at a young age, genetic testing might be recommended to assess your risk.

Physical Traits: Certain traits like fair skin, red or blonde hair, and a high number of atypical moles can also be genetically influenced and raise your risk.

💊 Genetics and Treatment Decisions

Somatic Mutations: These are mutations that occur in melanoma cells themselves (not inherited). The most common is a mutation in the BRAF gene, found in about 50% of cutaneous melanomas. If your tumor has this mutation, you may be eligible for targeted therapies like BRAF and MEK inhibitors.

Other Mutations: Mutations in NRAS, KIT, and NF1 can also guide treatment choices, especially in non-cutaneous melanoma subtypes like acral or mucosal melanoma.

Tumor Microenvironment: Genetic profiling can reveal how your immune system interacts with the tumor, which helps determine whether immunotherapy (like checkpoint inhibitors) might be effective.

🧪 Should You Consider Genetic Testing?

Tumor Genetic Testing: This is often done automatically as part of your diagnosis to guide treatment.

Germline Genetic Testing: If you have a strong family history or are diagnosed at a young age, your care team might suggest testing for inherited mutations.

Clinical Trials: Some trials focus on patients with specific genetic profiles, offering access to cutting-edge therapies.

If you're comfortable sharing, I can help you explore what kind of melanoma you have (cutaneous, acral, mucosal, etc.) and whether any genetic testing has already been done. That could help us dig deeper into what your results might mean for your treatment path.

Would you like to explore how to interpret genetic test results or look into clinical trials tailored to your profile?

Input 5:

You're asking a powerful and important question—one that many adolescents and young adults (AYA) with melanoma are starting to explore more deeply. Genetics can play a role in both the development of melanoma and how it's treated, especially in younger patients like yourself. Here's a breakdown of what we know so far:

🧬 Genetic Factors in Melanoma Risk

Inherited Mutations: A small percentage of melanomas are linked to inherited gene mutations. The most well-known is CDKN2A (p16), which increases melanoma risk and may be passed down in families.

Other Genes: Mutations in BRCA2, PTEN, TP53, BAP1, and MITF have also been associated with increased melanoma risk, often alongside other cancers.

Family History: If you or close relatives have had multiple melanomas or other cancers (like pancreatic, breast, or brain tumors), genetic testing might be recommended to look for inherited syndromes.

🧪 Genetic Testing and Counseling

Who Should Consider It: Genetic testing is typically offered to those with:

Multiple melanomas

Early-onset melanoma (before age 45)

A strong family history of melanoma or related cancers

What It Can Reveal: Testing can identify mutations that may influence your risk and help guide screening for other cancers. However, not all mutations directly affect treatment decisions.

💊 Genetics and Treatment Decisions

Targeted Therapies: Some melanomas—especially metastatic ones—are driven by somatic mutations (not inherited) like BRAF, NRAS, or KIT. These mutations can be identified through tumor profiling and may guide treatment choices.

For example, BRAF-positive melanoma can be treated with BRAF inhibitors like vemurafenib or dabrafenib.

AYA-Specific Considerations: Melanoma in younger patients may have different molecular characteristics and histologic features compared to older adults. This can affect how it's diagnosed and managed.

🧠 What This Means for You

If you haven’t already had tumor genetic profiling, it’s worth discussing with your oncologist—it can directly impact your treatment plan.

If you have a family history of melanoma or other cancers, a referral to a genetic counselor might help clarify your inherited risk and inform long-term care.

Even if no inherited mutation is found, understanding your tumor’s genetic makeup can open doors to personalized therapies and clinical trials.

If you'd like, I can help you prepare questions for your care team or explore what genetic counseling might look like. You're navigating something complex and courageous—and you're not alone in it.

**Question 5: I am an adolescent/young adult patient with metastatic melanoma and am currently receiving treatment. I want more information about the long-term side effects of treatment.**

Input 1:

Thank you for sharing that—it's incredibly important to understand what lies ahead, especially when you're navigating something as intense as metastatic melanoma treatment. Adolescents and young adults (AYAs) like yourself often face unique challenges, and the long-term effects of treatment can vary depending on the type and intensity of therapies used.

Here’s a breakdown of what you might want to keep an eye on:

🧠 Common Long-Term Side Effects in AYAs

These effects may appear months or even years after treatment ends:

Fertility issues: Chemotherapy and radiation can affect reproductive health. Fertility preservation options should be discussed before treatment begins2.

Heart and lung problems: Certain chemotherapy drugs and chest radiation may increase the risk of cardiopulmonary complications later in life.

Secondary cancers: There's a slightly increased risk of developing another cancer due to the effects of treatment2.

Hormonal imbalances: Treatments can affect the endocrine system, leading to thyroid issues or other hormone-related conditions3.

Bone health: Some therapies may impact bone density, increasing the risk of osteoporosis or fractures.

Cognitive changes: “Chemo brain” or other neurological effects can impact memory, attention, or processing speed.

Psychosocial impacts: Anxiety, depression, and challenges with identity or relationships are common and deserve just as much attention as physical side effects.

🩺 Why Follow-Up Care Matters

Regular follow-up is essential to catch and manage these effects early. The Children’s Oncology Group guidelines offer tailored recommendations for long-term monitoring in young cancer survivors.

💬 What You Can Do

Talk to your care team about survivorship planning.

Ask about fertility preservation, mental health support, and screening for late effects.

Keep a record of your treatments—this helps future providers understand your risks.

Input 2:

Thank you for sharing that—it's incredibly important to understand what lies ahead, especially when you're navigating something as intense as metastatic melanoma treatment. Adolescents and young adults (AYAs) face unique challenges because your body is still developing, and treatments can have long-term ripple effects that differ from those in older adults.

Here’s a breakdown of potential long-term side effects you might want to discuss with your care team:

🧠 Cognitive and Emotional Effects

Memory and concentration issues (sometimes called “chemo brain”)

Anxiety, depression, or PTSD related to the cancer experience

Social and identity challenges, especially around independence and relationships

❤️ Cardiopulmonary Complications

Heart damage from certain chemotherapy drugs or chest radiation

Lung issues, including reduced capacity or scarring

🧬 Secondary Cancers

Increased risk of developing another cancer later in life, especially if radiation or certain chemo agents were used2

🧒 Growth and Hormonal Disruption

Hormone deficiencies due to damage to endocrine glands

Delayed growth or puberty, depending on age and treatment type

👶 Fertility Concerns

Impaired fertility is a major concern for AYAs. Some treatments can affect sperm or egg production permanently2

Fertility preservation options (like egg or sperm banking) should ideally be discussed before treatment begins

🦴 Bone and Organ Health

Bone density loss, increasing risk of fractures later

Kidney or liver damage, depending on drug exposure

Chronic pain or swelling in certain areas

🧍‍♀️ Psychosocial and Lifestyle Impacts

Challenges with returning to school or work

Navigating insurance, financial stress, and long-term follow-up care

Feeling “different” from peers due to scars, fatigue, or emotional shifts

📋 What You Can Do

Ask your team about the Children’s Oncology Group (COG) long-term follow-up guidelines, which are tailored for young survivors and available in patient-friendly formats

Keep a treatment summary so future providers understand your history

Schedule regular screenings for heart, lung, fertility, and mental health

You can explore more in-depth guidance from the American Cancer Society and BMJ’s review on long-term chemotherapy effects in young adults.

If you’d like, I can help you build a personalized checklist of questions to ask your oncologist or survivorship team. Would that be helpful?

Input 3: Thank you for sharing that—it's incredibly important to understand what lies ahead, especially when you're navigating something as intense as metastatic melanoma treatment. The long-term side effects can vary depending on the type of treatment you're receiving (like chemotherapy, immunotherapy, targeted therapy, or radiation), your age, and your overall health. For adolescents and young adults, there are some unique considerations because your body is still developing, and that can influence how treatments affect you over time.

Here’s a breakdown of potential long-term effects you might want to discuss with your care team:

🧠 Cognitive and Neurological Effects

Memory and concentration issues (sometimes called “chemo brain”)

Peripheral neuropathy (numbness or tingling in hands and feet)

❤️ Cardiopulmonary Effects

Heart damage from certain chemotherapy drugs or chest radiation

Lung problems, especially if radiation was involved

🧬 Endocrine and Hormonal Changes

Hormone imbalances affecting growth, metabolism, or sexual development

Thyroid dysfunction, especially after radiation to the neck

🧒 Fertility and Reproductive Health

Reduced fertility or infertility due to chemotherapy or radiation

Menstrual irregularities or early menopause in females

🦴 Musculoskeletal and Growth Issues

Bone density loss or increased risk of fractures

Growth delays, particularly if treatment occurred during puberty

🩺 Secondary Cancers and Organ Damage

Increased risk of second cancers later in life

Kidney or liver damage depending on the drugs used

🧘 Psychosocial and Emotional Health

Anxiety, depression, or PTSD related to the cancer experience

Social reintegration challenges, especially with peers or school/work

📋 Monitoring and Follow-Up

The American Cancer Society and the Children’s Oncology Group offer survivor guidelines tailored to young adults that outline what to monitor and how often. These include screening for late effects and strategies to manage them.

It’s a lot to take in, but you’re not alone in this. Your oncology team should help you build a survivorship care plan that includes regular follow-ups, screenings, and support for both physical and emotional health. If you’d like, I can help you draft questions to ask your doctor or explore ways to track your symptoms and appointments.

Input 4:

Thank you for sharing that—it's incredibly important to understand what lies ahead, especially when you're navigating something as intense as metastatic melanoma treatment at a young age. The long-term side effects can vary depending on the specific therapies you're receiving (like immunotherapy, targeted therapy, chemotherapy, or radiation), but here’s a breakdown of what’s commonly seen in adolescents and young adults:

🧠 General Long-Term Effects in Young Adults

Because your body is still developing, treatments can have unique impacts compared to older adults. According to the American Cancer Society and other expert sources2, here are some key areas to watch:

🫀 Physical Health

Heart and lung issues: Some chemotherapy drugs and chest radiation can affect heart and lung function over time.

Fertility concerns: Treatments may impair reproductive health, so fertility preservation discussions are crucial before starting therapy.

Bone health: Certain drugs may weaken bones or affect growth.

Kidney and liver function: Long-term toxicity can impact these organs depending on the drugs used.

🧬 Cancer-Related Risks

Second cancers: There's a slightly increased risk of developing another cancer later in life due to treatment exposure.

Hormonal imbalances: Radiation or surgery near hormone-producing glands can lead to deficiencies.

👁️ Sensory and Cognitive Effects

Hearing or vision changes: Some treatments may affect sensory nerves or tissues.

Cognitive changes: “Chemo brain” or memory/concentration issues can persist for months or years.

🧠 Mental and Emotional Health

Psychosocial challenges: Anxiety, depression, and PTSD-like symptoms are not uncommon and deserve just as much attention as physical effects.

Social reintegration: Returning to school, work, or relationships can be complicated and emotionally taxing.

🩺 What You Can Do

Regular follow-ups: Long-term survivorship care plans are essential. Ask your care team about the Children’s Oncology Group (COG) guidelines, which offer tailored screening and monitoring strategies.

Lifestyle support: Nutrition, exercise, and mental health care can help mitigate some long-term effects.

Documentation: Keep a detailed record of your treatments—this helps future providers understand your risks and needs.

If you’d like, I can help you explore survivorship programs, connect you with resources for fertility preservation, or even help draft questions to ask your oncologist. You're not alone in this—let’s make sure you're equipped for the road ahead.

Input 5:

Thank you for sharing that—it's incredibly important to understand what the road ahead might look like. As an adolescent or young adult (AYA) with metastatic melanoma, your body is still developing in ways that can make you more sensitive to cancer treatments. Long-term side effects can vary depending on the type and intensity of treatment, but here’s a breakdown of what’s commonly seen in AYA patients:

🧠 Common Long-Term Side Effects of Treatment

Fertility issues: Chemotherapy and radiation can affect reproductive health. Fertility preservation options are often discussed before treatment begins.

Heart and lung problems: Certain chemotherapy drugs and radiation to the chest may increase the risk of cardiovascular or pulmonary complications later in life.

Secondary cancers: There's a slightly increased risk of developing another cancer years after treatment, especially if radiation or certain chemo agents were used1.

Hormonal imbalances: Treatments can affect glands like the thyroid or pituitary, leading to hormone deficiencies.

Bone health: Some therapies may impact bone density, increasing the risk of osteoporosis or fractures.

Cognitive and emotional effects: Memory, attention, and emotional regulation can be affected, especially if treatment involved the central nervous system.

Kidney or liver function: Long-term organ monitoring may be necessary depending on the drugs used.

🩺 What You Can Do

Regular follow-ups: These are essential to catch late effects early. Your care team should tailor a survivorship plan based on your treatment history.

Ask about COG guidelines: The Children’s Oncology Group has long-term follow-up recommendations specifically for young cancer survivors. You can explore them on their survivorship site.

Mental health support: Psychosocial challenges are common, and talking to a counselor or joining a support group can be incredibly helpful.

If you’d like, I can help you build a personalized list of questions to ask your oncologist or even walk through what a survivorship care plan might include. You're not alone in this—there’s a whole network of support and knowledge to help you thrive beyond treatment.
