# Supplementary material for: Quality, Empathy, and Readability of AI Chatbot Responses to the Survivorship Needs of Adolescents and Young Adults With Melanoma: Evaluation Study
Source: JMIR Cancer. 2026 Mar 26;12:e84234. doi: 10.2196/84234 (PMC13020680; doi:10.2196/84234)
Supplement: Multimedia Appendix 5 [file cancer-v12-e84234-s005.docx]

Multimedia Appendix 5: Expanded Best Practices Table

| **Best Practices for AI Chatbot Responses to express empathy** | | |
| --- | --- | --- |
| **Best Practices (↑ PETS scores)** | **Downfalls (↓ PETS scores)** | **Examples** |
| Open with empathy and understanding | Robotic or clinical tone | Acknowledge diagnosis, saying “I’m sorry you’re going through this,” or validating fear/stress.” vs. “I understand you are an adolescent/young adult patient with metastatic melanoma…”, starting immediately with medical jargon, sounding cold or impersonal. |
| Follow-up & clarification questions | One-way “information dump" | “Would you like me to help you prepare questions for your oncologist, → asking “Can you tell me more?” or “Did I cover what you were hoping for?” vs. No follow up or clarification questions |
| Symptom and Side effect explanations in understandable terms | List of side effects or clinical jargon | In discussing possible pancreatic insufficiency 2/2 treatment: "You may notice you feel more tired or thirsty which could be a sign of these hormonal changes" vs. No explanation of how to recognize these changes at home, only providing name of treatment and name of hormone affected, or listing severe side effects that are not patient-specific without a full clinical picture |
| Medical disclaimers & boundaries | Overstep scope | “The information provided here is for educational purposes and should not replace a consultation with your doctor” vs. “I can help interpret your genetic testing results.”, “Have you considered clinical trials?” |
| Cites credible resources | Lack citations or specific resources | When discussing support providing links to AYA resources- Stupid Cancer, NCCN, ACS, fertility & survivorship resources vs. suggesting "therapy" or "support" but no guide on where to find resources |
| Specific actionable coping strategies | Generic or overwhelming advice | “Make a symptom diary, here is how...”; “try meditation strategies such as...”; “try box breathing here is how you do it” vs. “Do weekly self-exams” which is vague and puts pressure on the patient to discover new illness, instigating anxiety and fear or “fertility preservation discussions are crucial before starting therapy” which overlooks the reality that many patients are not given the opportunity to discuss fertility preservation before beginning treatment |
| Validates and justifies patient's emotional response | Naming emotions without explanation or context | AYA tailored and validating response: "For AYAs this fear can be... more intense because... life stage focused on building a future... feel isolated (from peers).., faced with uncertainty and loss of control over your body and future." vs something general that restates emotion expressed: "I'm sorry you're scared" |
| Inclusive, empathetic language that normalizes and reassures | Emotionally shallow or "empathy sandwich" automated response | "You're not alone in this, and it's important to know that there are ways to cope with that fear and take steps toward feeling more supported and in control." vs brief and generic emotional statements ("it can be difficult") |
| AYA-specific | Adult-centric or non-specific oncology framing | Providing an AYA-specific section that covers fertility, dating, school, work, independence, peer support vs. Presenting information as if for general oncology patients, missing developmental relevance and differences in needs, one response even mentioned “Children’s Oncology guidelines” |
| Demonstrates interest in the patient | Lack of interest or personalization | “I’d like to help you think about how this affects your daily life", "Do you have any other questions" vs. No personalization, failure to engage with the patient |
| Structured and concise guides | Clinical jargon overload | Step-by-step plans, checklists, "Actionable steps you can take this week", or “what to expect” pathways vs long, dense paragraphs filled with clinical jargon without explanations |
| Provides empathy in all contexts | Overlooks unspoken emotional needs | In inputs with less emotion (3-5): "I'm really sorry to hear that you're dealing with metastatic melanoma- what you're going through is incredibly challenging" vs no empathetic statements, simply opening with "Thank you for your question" or diving directly into biology |
