# Supplementary material for: Quality, Empathy, and Readability of AI Chatbot Responses to the Survivorship Needs of Adolescents and Young Adults With Melanoma: Evaluation Study
Source: JMIR Cancer. 2026 Mar 26;12:e84234. doi: 10.2196/84234 (PMC13020680; doi:10.2196/84234)
Supplement: Multimedia Appendix 6 [file cancer-v12-e84234-s006.docx]

**Google Gemini**

**Question 1: I am an adolescent/young adult patient with metastatic melanoma and am currently receiving treatment. I want more information about what will happen when treatment finishes.**

Input 1:

"Navigating life after completing treatment for metastatic melanoma is a complex and highly personal journey. While I can provide you with general information, it is crucial to remember that your specific experience will be unique. You should always discuss your questions and concerns with your healthcare team, including your oncologist, nurses, and other specialists, as they are the best source of information about your individual case.

Here's a breakdown of what you can generally expect after finishing treatment for metastatic melanoma:

1. Follow-Up Care and Monitoring

This is a critical part of your post-treatment plan. Your healthcare team will monitor you closely to manage any lingering side effects and check for any signs that the cancer might have returned (recurrence).

Frequency: The schedule for follow-up appointments varies depending on your specific diagnosis, the stage of your melanoma, and the treatments you received. It's often more frequent in the first few years after treatment, as this is when the risk of recurrence is highest.

What to Expect: Follow-up visits typically involve a physical exam, a skin exam, and a discussion of any new symptoms or side effects you may be experiencing. Your doctor may also order imaging tests, such as CT scans, MRIs, or ultrasounds, to check for cancer spread.

Self-Exams: Your doctor will likely teach you how to perform regular self-exams of your skin and lymph nodes. It's important to report any new lumps, growths, or other unusual changes to your healthcare team right away.

2. Potential Long-Term and Late Side Effects

Many of the side effects you experienced during treatment may improve or disappear over time, but some can persist or appear months or even years later. These are known as long-term or late effects. The specific side effects you might face depend on the type of treatment you received (e.g., immunotherapy, targeted therapy, radiation therapy).

Immunotherapy: Immunotherapy works by activating your immune system, which can sometimes lead to inflammation in different organs. Long-term effects can include fatigue, joint pain, skin rashes, or hormonal imbalances, particularly affecting the thyroid gland.

Targeted Therapy: Common side effects can include fever, fatigue, rash, and diarrhea. Some of these may continue after treatment ends.

Other Treatments: Depending on your specific treatment history, you may also experience other long-term side effects. Your healthcare team can provide you with a list of potential issues to watch for.

It's essential to talk to your healthcare team about any new or ongoing symptoms, as early treatment of these side effects can help prevent them from becoming more severe.

3. Creating a Survivorship Care Plan

A survivorship care plan is a personalized document that can help you and your future healthcare providers manage your health after cancer treatment. It's a great tool for transitioning from active treatment to long-term follow-up.

This plan typically includes:

A summary of your diagnosis and treatments.

A schedule for your follow-up exams and tests.

A list of possible late- or long-term side effects and what to do if they occur.

Suggestions for healthy living, such as diet, exercise, and sun safety.

Information about emotional and psychological support resources.

4. Emotional and Mental Health

Finishing treatment can bring a mix of emotions. You might feel relieved, but you may also experience anxiety, fear of recurrence, or ""scanxiety"" (anxiety before and during follow-up scans). As an adolescent or young adult, you also face unique challenges related to your age, such as navigating school, work, relationships, and fertility.

Emotional Support: It's important to acknowledge your feelings and seek support. Talking to a therapist, a social worker, or joining a support group with other cancer survivors can be very helpful.

AYA-Specific Resources: There are organizations and programs specifically designed for adolescents and young adults with cancer that can address the unique social, emotional, and practical challenges you may be facing.

Fear of Recurrence: This is a very common concern. Your healthcare team can help you develop strategies for managing this fear and will provide you with clear guidance on what to look for and when to contact them.

5. Fertility and Reproductive Health

Cancer treatments can sometimes affect fertility. As an adolescent or young adult, this is a particularly important topic to address. If you didn't have a chance to discuss this before treatment, or if you have questions now, it is not too late to talk to your healthcare team. They can provide resources and referrals to fertility specialists who can discuss your options for family planning in the future.

Remember, you are not alone in this. Your healthcare team is there to support you every step of the way, and there are many resources available to help you navigate life after treatment. Don't hesitate to ask questions and advocate for your needs."

Input 2:

"Navigating life after cancer treatment can be a complex journey, and it's completely normal to have questions about what comes next. Here is a breakdown of what you can expect as you transition from active treatment to survivorship.

Survivorship Care Plan

As your treatment for metastatic melanoma ends, your healthcare team will likely work with you to develop a survivorship care plan. This is a personalized document that summarizes your treatment and outlines your ongoing care. A survivorship care plan typically includes:

Treatment Summary: A record of your diagnosis, the treatments you received (e.g., immunotherapy, targeted therapy, surgery, radiation), and the dates of your treatments.

Follow-Up Schedule: A detailed plan for your future appointments and tests. This schedule is tailored to your specific situation, including the stage of your melanoma and the treatments you had. Follow-up is often more frequent in the first few years after treatment, when the risk of recurrence is highest.

List of Potential Side Effects: Information about possible long-term side effects (also known as late effects) of your treatment. This helps you and your doctors recognize and manage any health issues that may arise months or even years down the road.

Symptoms to Watch For: A list of symptoms that could indicate a recurrence of melanoma, and clear instructions on who to contact if you experience them. This is crucial for early detection.

Health and Wellness Recommendations: Guidance on healthy lifestyle habits, such as sun safety, diet, exercise, and mental health support.

Follow-Up Care and Monitoring

Follow-up care is a vital part of staying healthy after treatment. Your follow-up plan will likely involve a combination of different tests and appointments:

Physical and Dermatologic Exams: Your doctor will perform regular physical exams, including a thorough skin check, to look for any new growths or changes.

Imaging Tests: Depending on your situation, you may have regular imaging tests, such as CT scans, MRI scans, or PET scans, to monitor for any signs of the cancer returning. The frequency of these scans will be outlined in your survivorship plan.

Blood Tests: You may have regular blood tests to check your general health and monitor for any signs of long-term side effects, particularly if you received immunotherapy.

Potential Long-Term Side Effects

The specific side effects you might experience after treatment depend on the type of therapy you received.

Immunotherapy: Immunotherapy works by activating your immune system to fight cancer. While often very effective, this can sometimes lead to an overactive immune response that causes inflammation in various organs. This can result in long-term side effects affecting the skin, liver, intestines, or endocrine glands (like the thyroid). Your healthcare team will monitor you closely for these issues.

Targeted Therapy: Targeted therapies are designed to attack specific genetic mutations in cancer cells. While side effects are often temporary, long-term issues can include fever, fatigue, joint pain, and skin problems.

Surgery: If you had surgery, long-term effects could include changes in sensation, limited range of motion in a limb, or chronic swelling (lymphedema) if lymph nodes were removed.

Emotional and Psychological Well-being

Completing treatment is a huge milestone, but it can also be a time of mixed emotions. Many survivors experience anxiety about recurrence, depression, or difficulty readjusting to ""normal"" life. It's important to remember that these feelings are valid and that you don't have to go through them alone. Your survivorship care plan should include resources for psychological support, such as:

Support Groups: Connecting with other young adults who have been through similar experiences can be incredibly helpful.

Counseling or Therapy: A professional can help you process your emotions and develop coping strategies.

Oncology Social Workers: These professionals can connect you with resources for managing the social and financial aspects of life after cancer.

This information is a general overview. It is essential to have an open and honest conversation with your healthcare team to create a survivorship plan that is right for you. They are your best resource for understanding the specifics of your individual care."

Input 3:

"Finishing your treatment for metastatic melanoma is a significant milestone, and it's completely normal to have questions and concerns about what comes next. The period after active treatment is often referred to as ""survivorship,"" and it involves a new phase of care focused on monitoring your health, managing any long-term side effects, and supporting your overall well-being.

Here is a breakdown of what you can generally expect after your treatment finishes:

1. Follow-Up Care and Monitoring

Follow-up care is crucial for the first several years after treatment. The goal is to monitor for any signs of the melanoma returning (recurrence) and to address any new or ongoing health issues. Your follow-up schedule will be personalized based on your specific case, including the stage of your melanoma and the treatments you received.

Frequency of Appointments: You will have regular appointments with your healthcare team, which may include your oncologist, a surgeon, and a dermatologist. These appointments are usually more frequent in the first 2-5 years and may become less frequent over time. For example, appointments might be scheduled every 3-6 months initially, and then annually after a certain period.

Physical Exams and Skin Checks: Your doctor will perform physical exams at each appointment, including a thorough skin check to look for any new growths, moles, or abnormal areas. They will also check for enlarged lymph nodes. Your healthcare team will likely teach you how to perform monthly self-exams and what to look for.

Imaging and Blood Tests: Your doctor may recommend regular imaging tests, such as CT scans, MRI scans, or PET scans, to check if the cancer has spread to other parts of your body. These tests are often done for a few years after treatment, especially for more advanced stages. You may also have regular blood tests to check your general health and monitor for any potential long-term side effects of treatment.

2. Potential Long-Term Side Effects

While many side effects from treatment go away after it's finished, some can be long-lasting or appear months or even years later. It's important to be aware of these ""late effects"" and to talk to your healthcare team about any new symptoms you experience.

Immunotherapy Side Effects: If you received immunotherapy, side effects can sometimes appear months after treatment. This is because immunotherapy works by activating your immune system, and in some cases, this can lead to inflammation in different organs. Some potential long-term effects include:

Endocrine problems: This can affect glands like your thyroid or pituitary, leading to hormonal imbalances that may require long-term medication.

Skin reactions: Itchy rashes or changes in skin pigmentation can occur.

Fatigue: This is a common and often long-lasting side effect.

Surgery Side Effects: If you had surgery, especially to remove lymph nodes, you might experience issues such as:

Lymphedema: Chronic swelling in the arm or leg where the surgery was performed.

Decreased range of motion: Stiffness or limited movement in the affected area.

3. Creating a Survivorship Care Plan

A survivorship care plan is a written summary of your cancer treatment and a roadmap for your future care. It is a valuable tool that can help you and all your healthcare providers manage your health going forward. A good survivorship care plan should include:

A summary of your diagnosis and the treatments you received.

A schedule for your follow-up appointments and recommended tests.

Information about potential long-term and late side effects of your treatment.

Recommendations for healthy living, such as diet, exercise, and sun safety.

Resources for emotional support, financial concerns, and other needs.

4. Emotional and Psychosocial Support

The end of treatment can bring a mix of emotions, including relief, anxiety about recurrence, and a feeling of being ""left alone"" after being so closely monitored. As an adolescent/young adult (AYA), you may also be dealing with unique challenges related to school, career, relationships, and fertility.

Mental Health: It is very common to experience anxiety or depression after cancer treatment. Talking with a mental health professional who specializes in cancer survivorship can be incredibly helpful.

Support Groups: Connecting with other AYA cancer survivors can help you feel less isolated and provide a space to share your experiences and coping strategies.

Specialized Resources: Organizations like the Cactus Cancer Society, Stupid Cancer, and the Ulman Foundation focus specifically on the unique needs of adolescents and young adults affected by cancer. They offer everything from support groups and creative workshops to financial and educational resources.

5. Taking an Active Role in Your Health

You are a key member of your healthcare team. It is essential to be proactive in your post-treatment care:

Report Symptoms: Don't wait until your next scheduled appointment to report any new or persistent symptoms that concern you.

Maintain a Healthy Lifestyle: Focus on a balanced diet, regular exercise, and protecting your skin from the sun.

Ask Questions: Don't hesitate to ask your doctors questions about your care plan, potential side effects, and available resources.

Remember, the journey doesn't end with the last treatment. It transitions into a new phase of living well and staying healthy. Your healthcare team is there to support you every step of the way.

Sources"

Input 4:

"Completing your cancer treatment is a significant milestone, and it's natural to have questions about what comes next. As an adolescent or young adult (AYA) patient, you may face specific challenges and considerations that are different from older adults. Here is an overview of what you can expect when treatment finishes.

Follow-Up Care and Monitoring

Even after you complete treatment, your doctors will want to monitor you closely. This is a crucial part of your long-term care, often called ""survivorship."" Your follow-up care plan will be personalized based on your specific type of melanoma, the stage it was at, and the treatments you received.

Regular Appointments: You can expect to have regular follow-up visits with your healthcare team, including your oncologist and dermatologist. These visits may be scheduled every few months at first, and then become less frequent over time.

Physical Exams: During these appointments, your doctors will perform a physical exam, paying close attention to your skin and lymph nodes to check for any new growths or changes.

Imaging and Tests: Depending on your situation and risk of recurrence, you may have imaging tests like CT scans, MRIs, or ultrasounds to check if the cancer has returned. Blood tests may also be used to monitor your general health and check for potential long-term side effects from your treatment.

Self-Exams: Your doctor will likely teach you how to do regular self-exams of your skin and lymph nodes, and it's important to report any new or unusual symptoms to your healthcare team right away.

Potential Long-Term Side Effects

Cancer treatments can cause side effects that may not appear until months or even years after treatment has finished. It is important to be aware of these potential late effects, so you can address them with your healthcare team. The specific side effects you might experience will depend on the type of treatment you received (e.g., immunotherapy, targeted therapy, radiation, surgery).

Some potential long-term side effects may include:

Physical Effects:

Fatigue: Many cancer survivors experience overwhelming tiredness that can last for a long time.

Skin Changes: Immunotherapy can cause skin issues like rashes or changes in pigmentation.

Hormonal Issues: Treatments like immunotherapy can cause inflammation in the endocrine system (e.g., thyroid, pituitary gland), which may require permanent hormone supplements.

Nerve Damage: Some treatments can cause nerve-related problems.

Other Organ-Specific Issues: Treatments can sometimes lead to inflammation in organs like the bowels or lungs.

Emotional and Psychological Effects:

Fear of Recurrence: It is very common to feel anxious between appointments and worry that the cancer will come back.

Depression and Anxiety: Many melanoma survivors report higher rates of anxiety and depression compared to the general population.

Changes in Body Image: Treatment may cause visible scars or skin changes that can affect how you feel about your body.

Coping with Life Changes: As a young adult, you may have unique challenges related to your cancer experience, such as feeling isolated from your peers, or feeling that your life has been put on hold.

Survivorship Care Plans

A survivorship care plan is a document that can help you and your healthcare team manage your health after treatment. It may include:

A summary of your cancer diagnosis and all the treatments you've received.

A schedule for your follow-up appointments and recommended tests.

A list of potential long-term side effects to watch for and who to contact if you have concerns.

Recommendations for a healthy lifestyle, including diet and exercise.

Resources and referrals for support, such as a social worker, nutritionist, or psychologist.

You can ask your healthcare team to help you create a personalized survivorship care plan.

Finding Support

The transition from active treatment to survivorship can be challenging. It's important to build a support system and address your physical and emotional needs.

Mental Health Professionals: Consider talking to a therapist or counselor who specializes in working with cancer patients, particularly adolescents and young adults. They can help you with coping strategies, managing anxiety, and navigating a new sense of ""normal.""

Support Groups: Connecting with other young adults who have been through a similar experience can be incredibly helpful. You can find both in-person and online support groups that are specifically for AYA cancer survivors.

Family and Friends: Be open with the people you trust about what you are going through. A counselor can also help you and your family communicate and work through the challenges together.

Resources: Organizations like the National Cancer Institute (NCI) and others often have resources and programs dedicated to helping young adults with cancer.

Living with the uncertainty of a metastatic melanoma diagnosis is not easy. It’s important to acknowledge your feelings and find support. Focusing on the things you can control, like your follow-up care and your overall well-being, can help you regain a sense of empowerment."

Input 5:

"Navigating life after cancer treatment can be a complex journey, and it's understandable to want information about what to expect. As an adolescent or young adult (AYA) patient, you have a unique set of needs that your healthcare team will need to address as you transition to survivorship. Here's a breakdown of what typically happens when treatment for metastatic melanoma finishes.

The Shift to Survivorship and Follow-up Care

The period after active treatment is often referred to as survivorship. This phase is an essential part of your cancer journey, and it focuses on your long-term health and well-being. A critical component of survivorship is follow-up care, which is designed to:

Monitor for recurrence: Your medical team will want to watch for any signs that the melanoma may have returned (recurred). The risk of recurrence is generally highest in the first five years after treatment, but long-term follow-up is important since melanoma can come back at any time.

Manage long-term and late side effects: Your doctors will help you manage any side effects from your treatment that persist or emerge months or even years later.

Promote overall health: Your care team will provide guidance on healthy lifestyle choices to support your recovery and reduce your risk of other health issues.

Follow-up visits are typically scheduled on a regular basis, often every 3 to 12 months, depending on your specific case and risk of recurrence. These appointments will usually include a physical exam, a skin exam, and a discussion about any new symptoms or concerns you might have. Your doctor may also order imaging tests (like a CT scan or MRI) or blood tests to check on your health.

The Survivorship Care Plan

A key resource for you after treatment is a survivorship care plan. This is a personalized document that summarizes your cancer journey and outlines your ongoing care. It's a valuable tool that can help you and your other doctors (like your primary care physician) understand your specific needs. A good survivorship care plan will include:

A summary of your cancer diagnosis and treatments.

A schedule for future follow-up appointments and screenings.

A list of potential long-term and late side effects to watch for.

Contact information for your healthcare team.

Guidance on healthy living and managing any physical or emotional challenges.

If your healthcare team has not yet discussed a survivorship care plan with you, it's a good idea to ask about it.

Potential Long-Term and Late Side Effects

The side effects you might experience after treatment depend on the specific therapies you received. Here are some of the most common long-term and late effects associated with various melanoma treatments:

Immunotherapy: These treatments can sometimes cause the immune system to remain overactive, leading to inflammation in various organs. This can result in side effects like fatigue, skin rashes, joint pain, and hormonal imbalances (such as thyroid problems). These issues can sometimes emerge months after treatment has ended.

Targeted Therapy: Side effects can vary depending on the specific drugs, but common long-term issues can include fever, fatigue, joint pain, and skin problems.

Surgery: Depending on the extent and location of your surgery, you may experience chronic pain, decreased range of motion, or swelling (lymphedema) in the affected area.

Radiation Therapy: Side effects from radiation, such as skin irritation, can sometimes persist after treatment.

It's crucial to report any new or worsening symptoms to your healthcare team, even if you are no longer in active treatment. Early intervention can often help manage these side effects more effectively.

Unique Considerations for Adolescents and Young Adults (AYAs)

As an AYA patient, you may face unique challenges as you transition to survivorship. Your cancer diagnosis may have interrupted key developmental milestones, and you may be balancing school, work, and social life with your health needs. Your care team should be mindful of this and provide support tailored to your age group. This can include:

Psychosocial Support: Cancer can have a significant emotional and social impact. Resources like support groups, social workers, and counselors can help you cope with your feelings, body image changes, and the challenges of reintegrating into your life after treatment.

Fertility Preservation: If this was not a part of your initial treatment planning, it's an important topic to discuss with your healthcare team.

Age-appropriate communication: You have the right to be a central part of your healthcare decisions. Your care team should communicate with you in an age-appropriate way, respecting your autonomy while also involving your family or other support systems as you desire.

Remember, you are not alone in this. Your healthcare team, including your oncologist, nurses, and other specialists, is there to help you navigate this next phase of your life. Don't hesitate to ask questions and advocate for the care and support you need."

**Question 2: I am an adolescent/young adult patient with metastatic melanoma and am currently receiving treatment. In the last month I have become more worried about my cancer spreading. What do I need to know and what steps can I take to cope with this fear?**

Input 1:

"Thank you for reaching out with your concerns. It is completely understandable to feel worried about your cancer spreading, especially as a young person. This fear, often called ""fear of recurrence,"" is a very common and normal part of the cancer experience, even for those currently in treatment. You are not alone in this feeling.

Here is some information and a list of steps you can take to help you cope with your fears and feel more in control.

What You Need to Know

Your Feelings Are Valid: It is important to acknowledge that fear and anxiety are normal reactions to a serious diagnosis like metastatic melanoma. Don't feel like you have to ""stay strong"" or hide your emotions.

The Unique Challenges of a Young Adult: As an adolescent or young adult (AYA), you face specific challenges that can amplify these fears. Your peers may not understand what you're going through, and you may feel like your life is on hold. Issues like fertility, education, and career goals can also be sources of worry.

Knowledge is Power: Uncertainty is a major driver of fear. By understanding your specific diagnosis and treatment plan, you can feel more empowered. Ask your medical team to explain everything in a way you can understand.

Coping is a Process: There will be good days and bad days. The skills you learn to cope with your fear will change over time and at different points in your cancer journey.

Steps You Can Take to Cope with Your Fear

1. Talk to Your Healthcare Team

Be Honest: Tell your doctor, nurse, or social worker about your fears. They are there to support you and can provide valuable information and resources.

Ask Specific Questions: Don't be afraid to ask for details. You could ask questions like:

""What is the goal of my current treatment?""

""What are the signs and symptoms of cancer spreading that I should watch for?""

""What tests will I need to monitor my cancer?""

""Can you help me understand my latest test results?""

""What can be done for any symptoms I'm having, like pain or fatigue?""

Bring Someone with You: It can be hard to remember everything your doctor says. Ask a family member or friend to join you at appointments to take notes and help you remember important information.

Request a Referral: Your healthcare team can connect you with mental health professionals who specialize in working with cancer patients, such as an oncology social worker or a psychologist.

2. Seek Psychosocial and Emotional Support

Find a Support Group: Connecting with other AYA patients with cancer can be incredibly helpful. Talking to people who have similar experiences can reduce feelings of isolation and provide practical advice. Many hospitals and cancer organizations offer online or in-person support groups specifically for young adults.

Consider Individual Counseling: A mental health professional, such as a psychologist or counselor, can teach you coping skills and relaxation techniques tailored to your needs. Therapies like Cognitive Behavioral Therapy (CBT) and Acceptance and Commitment Therapy (ACT) have been shown to be effective in managing anxiety related to cancer.

Explore Peer Mentoring: Some organizations can match you with a cancer survivor or a caregiver who has had a similar diagnosis. This one-on-one connection can provide a unique sense of understanding and support.

3. Focus on Self-Care and Lifestyle

Mind-Body Therapies: Practices like meditation, yoga, mindfulness, and deep breathing can help you manage stress and feel more relaxed.

Maintain a Routine: Whenever possible, try to maintain a consistent daily routine. This can include exercise, getting enough sleep, and eating nutritious meals.

Journaling: Writing down your thoughts and feelings can be a powerful way to process your emotions and reduce stress.

Find Joyful Activities: Make time for hobbies and activities that you love and that give your life meaning. This can help you feel more grounded and less focused on your fears.

4. Be Open with Loved Ones

Communicate Honestly: It can be difficult to talk about your fears with family and friends, but being open can help them understand what you're going through and allow them to support you more effectively.

Set Boundaries: It is also okay to let people know when you need space or when you're not up for discussing your cancer. You can choose who you want to see and when.

5. Manage Information Intake

Be Mindful of Online Searches: While it's natural to want to learn more, be cautious about where you get your information. Stick to reputable sources like the National Cancer Institute (NCI) and major cancer organizations.

Ask Your Doctor: If you find information that worries you, write it down and ask your doctor about it at your next appointment.

The fear you are experiencing is a heavy burden, but there are many resources available to help you carry it. Please do not hesitate to reach out to your medical team to begin exploring these options.

Sources"

Input 2:

"Navigating a diagnosis of metastatic melanoma as a young person can be incredibly challenging, and it's completely understandable to feel more worried about your cancer spreading. This fear is a normal part of the experience, and there are many resources and steps you can take to help you cope.

What You Need to Know

1. Acknowledge Your Feelings: It's important to recognize that your fears are valid. Fear, anxiety, and distress are common emotional responses to a cancer diagnosis, especially when dealing with a metastatic disease. These feelings are not a sign of weakness and are often tied to the uncertainty of your situation.

2. Seek Reliable Information: Gaining a better understanding of your specific diagnosis and treatment plan can help alleviate some of the fear. Ask your doctors and nurses to explain things in a way that makes sense to you. It's okay to ask the same questions more than once or to ask for a referral to a professional who can provide more details.

3. Understand Your Treatment Goals: Be sure you are clear on the goals of your current treatment. Is it to control the cancer, to manage symptoms, or both? Understanding this can help you set realistic expectations and feel more in control. Ask your healthcare team about the effectiveness of your current treatment and what to expect in the future.

4. The Importance of Communication: Open and honest communication with your healthcare team is crucial. They are your partners in this journey. Tell them directly that you are feeling more anxious and worried about your cancer spreading. This will allow them to screen you for distress and connect you with the right support services, which can include counseling, support groups, or other resources.

Steps You Can Take to Cope

1. Talk to Your Healthcare Team:

Prepare your questions: Write down your questions and concerns before your appointment. This ensures you don't forget anything important.

Bring a trusted person: Ask a family member or friend to come with you to appointments. They can help listen, take notes, and ask questions.

Express your concerns directly: Use clear language to let your doctor know about your increased anxiety. For example, you could say, ""I've been feeling more worried about my cancer spreading lately, and it's affecting my daily life.""

2. Find Emotional Support:

Oncology Social Workers: These professionals are specially trained to provide emotional and practical support for people with cancer. They can offer counseling and help you navigate the challenges of your diagnosis, including the fear of progression.

Support Groups: Connecting with other young adults who have metastatic melanoma can be incredibly helpful. Sharing your experiences with people who truly understand what you're going through can reduce feelings of isolation and provide a sense of community. Organizations like CancerCare, the Melanoma Research Foundation, and the AIM at Melanoma Foundation offer online and in-person support groups.

Counseling/Therapy: A therapist or counselor specializing in oncology can provide you with tools and strategies to manage your fear and anxiety. They can help you process your emotions and develop coping mechanisms.

3. Focus on What You Can Control:

Healthy Lifestyle: Maintaining healthy habits, such as eating nutritious foods, getting enough rest, and incorporating gentle exercise (if approved by your doctor), can improve your energy levels and overall well-being.

Mind-Body Therapies: Practices like meditation, yoga, or deep breathing can help you relax and focus on the present moment instead of dwelling on future fears. Many cancer centers and hospitals offer classes or workshops for patients.

Skin Checks and Follow-up Appointments: Staying on top of your self-exams and follow-up appointments with your dermatologist can be a proactive way to feel more in control. Knowing that you are actively monitoring your health and getting regular care can provide a sense of relief.

4. Look for Organizations and Resources Specifically for Young Adults:

The Samfund: This organization provides support for young adult cancer survivors as they recover from the financial and emotional impact of cancer.

Patient Advocacy Organizations: Groups like the Melanoma Research Foundation and AIM at Melanoma Foundation have specific resources for patients and caregivers, including online forums and educational materials.

Remember, you don't have to go through this alone. Reaching out for help is a sign of strength, and there are many people and organizations dedicated to supporting you through this journey.

Sources"

Input 3:

"Thank you for reaching out. It's completely understandable to feel worried about your cancer spreading, and you are not alone in having this fear. Many people with a cancer diagnosis experience anxiety and fear, often referred to as ""scanxiety"" or ""fear of recurrence."" This is a very common part of the cancer journey, especially for young adults who are facing unique challenges.

Here is what you need to know and some steps you can take to cope with these feelings:

What You Need to Know

1. Your Feelings Are Valid: It is normal and expected to feel scared, angry, and sad. A cancer diagnosis, particularly a metastatic one, is life-altering, and it's important to acknowledge and validate your own emotions. Your feelings are not a sign of weakness; they are a normal reaction to a difficult situation.

2. You Have a Support Team: Remember that you are not on this journey by yourself. You have a medical team—doctors, nurses, and social workers—who are there to help you medically and emotionally. They can provide resources and support to help you manage your mental health, which is just as important as your physical health.

3. Knowledge is Power: Gaining more knowledge about your specific diagnosis and treatment plan can sometimes help to reduce anxiety. Understanding the ""why"" and ""how"" of your treatment can give you a sense of control. However, be mindful of where you get your information. Sticking to trusted sources like your doctor and reputable cancer organizations is crucial to avoid misinformation that can increase fear.

Steps You Can Take to Cope

1. Talk to Your Medical Team:

Be Honest: Tell your doctors and nurses how you are feeling. It's important for them to know about your anxiety and fears so they can provide the right support.

Ask Questions: Write down your questions before your appointments. Ask about your treatment goals, what to expect from your treatment, and what symptoms you should watch for. Clarifying these things can help alleviate uncertainty.

Discuss Your Concerns: Your healthcare team can explain the tests they are performing and why, which may help you understand your situation better and reduce the fear of the unknown.

Ask for a Referral: Your doctor can connect you with an oncology social worker, counselor, or psychologist who specializes in helping people with cancer.

2. Connect with Others:

Support Groups: Joining a support group, either online or in person, can be incredibly helpful. Talking to other young adults who have metastatic melanoma can help you feel less alone and provide a space to share your experiences and learn from others who truly understand. Organizations like CancerCare and the Melanoma Research Foundation offer melanoma-specific support groups.

Peer-to-Peer Programs: Some organizations, such as IMPACT Melanoma, have ""buddy"" programs that pair you with a volunteer who has gone through similar experiences. This one-on-one connection can be a great source of support.

Family and Friends: It can be hard to talk to loved ones, but open communication can help you feel more supported. Let them know what you need and what they can do to help.

3. Focus on Your Mental and Physical Well-being:

Mind-Body Therapies: Practices like meditation, yoga, or deep breathing can help you relax and focus on the present moment instead of worrying about the future.

Physical Activity: Gentle exercise, if approved by your doctor, can improve your energy levels and reduce stress.

Healthy Habits: Maintain a consistent routine with healthy eating and getting enough sleep. These simple steps can make a big difference in how you feel.

Set Boundaries with Information: While it's good to be informed, it's also okay to take breaks from thinking about cancer. Try to engage in activities you enjoy that don't involve your diagnosis.

Remember that coping with the fear of cancer spreading is an ongoing process. It's okay to have good days and bad days. The key is to be kind to yourself and utilize the support systems available to you."

Input 4:

"I'm so sorry to hear you're going through this. It's completely understandable to feel more worried about your cancer spreading, especially as an adolescent or young adult. This fear is a very real and common experience for many patients. The good news is that there are many ways to cope with this anxiety and regain a sense of control.

Here is some information and a list of steps you can take to address your fears.

What to Know About Your Fear

It has a name: The fear of cancer coming back or getting worse is known as ""fear of recurrence or progression"" (FoP). It's one of the most common unmet needs for people with cancer. You're not alone in feeling this way.

It's often triggered: This fear can be triggered by specific events, such as upcoming scans, follow-up appointments, or even anniversaries of your diagnosis or treatment. It can also be sparked by new symptoms or seeing others with cancer experience progression.

It's more common in young adults: Studies show that a higher proportion of young adult patients (31-85%) report this fear compared to older adults. This highlights the importance of finding support that is tailored to your age group.

A certain amount of fear can be helpful: While high levels of fear can be distressing, a mild level of fear can be beneficial. It can help you stay motivated to take care of yourself, attend appointments, and be vigilant about your health.

Steps You Can Take to Cope

1. Talk to Your Healthcare Team

Be direct and honest: Tell your doctor, nurse, or social worker about your worries. They are there to help you.

Ask questions: One of the most effective ways to reduce fear is to address the unknown. Prepare a list of questions to ask at your next appointment. Here are some examples:

""Based on my current status, what are the goals of my treatment right now?""

""What tests will I need to monitor for changes in my cancer, and how often will they be done?""

""What symptoms should I watch for and report to you right away?""

""What can be done to manage my current symptoms, like pain or fatigue?""

""What resources or support options are available for me to deal with my anxiety?""

2. Seek Professional Emotional and Psychological Support

Oncology social workers: These professionals can provide free emotional and practical support. They can help you with counseling, finding support groups, and navigating financial concerns.

Counseling and therapy: Cognitive Behavioral Therapy (CBT) is a proven method for managing anxiety. A therapist can help you identify and reframe negative thought patterns and teach you relaxation techniques.

Young adult support groups: Connecting with other adolescents and young adults who have metastatic melanoma can be incredibly powerful. You can share experiences, ask questions, and build a community with people who truly understand what you're going through. Many organizations, like the Melanoma Research Foundation and CancerCare, offer online and in-person groups specifically for young adults.

3. Focus on Self-Care and Managing Your Body's Response to Anxiety

Mind-body practices: Techniques like deep breathing, mindfulness meditation, and guided imagery can help calm your nervous system and reduce the physical symptoms of anxiety.

Maintain a routine: Try to keep a consistent daily routine that includes exercise, healthy eating, and adequate sleep. These can help improve your energy levels and overall mood.

Physical activity: Regular exercise, even a short walk, can release endorphins that improve your mood and reduce tension.

Journaling: Writing down your thoughts and feelings can help you process them and gain perspective.

4. Empower Yourself with Knowledge (but set boundaries)

Learn from reliable sources: Understanding your diagnosis and treatment plan can give you a greater sense of control. Talk to your healthcare team and use reputable organizations like the American Cancer Society or the National Cancer Institute for information.

Avoid ""Dr. Google"": While it's tempting to search online, this can often lead to more fear and misinformation. Stick to trusted sources and discuss any new information with your doctor.

Know when to take a break: It's okay to limit your exposure to cancer-related news or discussions if they are causing you distress.

Remember, your feelings are valid, and it's a sign of strength to be seeking ways to cope. The fact that you are proactively looking for solutions is a huge step. You have a whole team of people—your doctors, nurses, social workers, and other patients—who are ready to support you."

Input 5:

"It's understandable and completely normal to feel worried about your cancer spreading, especially as a young person. The fear of recurrence or progression is a common and significant challenge for adolescents and young adults (AYAs) with cancer. Here's a breakdown of what you need to know and some steps you can take to cope with this fear.

What to Know About Metastatic Melanoma and Your Fears

Your fears are valid. Many AYA cancer patients experience heightened anxiety about their cancer coming back or progressing. This is often linked to the fact that you have a longer potential lifespan for the disease to recur and more life milestones that a recurrence could disrupt (e.g., school, career, relationships).

Knowledge is a tool. Uncertainty can fuel fear. While it's important to avoid becoming overly fixated, learning about your diagnosis and treatment can give you a sense of control. Ask your medical team specific questions about your prognosis, treatment plan, and what to expect. Knowing the symptoms to watch for and how often you'll have follow-up appointments can provide some reassurance.

Progress is being made. The field of metastatic melanoma treatment has seen significant advances, particularly with immunotherapy. Newer treatments have shown impressive results, with long-term survival rates improving. It's helpful to remember that your doctors are using the latest research and evidence to guide your care.

Your body can be a source of anxiety. It's common to worry that every ache, pain, or unusual sensation is a sign that the cancer has spread. This is a very real and often irrational fear. It's important to work on trusting your body and learning to differentiate between normal bodily sensations and potential cancer symptoms.

Steps You Can Take to Cope with Your Fear

Talk to Your Healthcare Team:

Be open about your fears. Your medical team, including your oncologist and nurses, are there to support you. Tell them you're feeling more anxious about the cancer spreading.

Ask for a referral. Many cancer centers have oncology social workers, psychologists, or counselors who specialize in helping young adults with cancer. They can provide tailored coping strategies and support.

Discuss your follow-up care plan. Understanding the schedule for your scans and check-ups can help you manage anxiety around these appointments.

Find a Support System:

Connect with other young adults with cancer. Feeling isolated is a common experience for AYA patients because your friends and family may not fully understand what you're going through. Connecting with peers who have similar experiences can be incredibly validating and comforting. Many organizations offer in-person and online support groups specifically for young adults.

Lean on your loved ones. Communicate your fears to your family and close friends. Let them know how they can best support you, whether it's through listening, providing distractions, or simply being there.

Consider a mental health professional. Therapies like Cognitive Behavioral Therapy (CBT) and Acceptance and Commitment Therapy (ACT) are effective for helping people reframe their thoughts and live in line with their values, even with the presence of fear.

Develop Coping Mechanisms:

Identify your triggers. Pay attention to what situations or events make your fear worse. Is it the anniversary of your diagnosis, an upcoming scan, or a news story about cancer? Once you recognize your triggers, you can plan how to manage them. For example, you might schedule a distraction (like a movie or a friend's visit) for the day of your scan.

Focus on what you can control. There are many things outside of your control, and dwelling on them can be overwhelming. Instead, focus on the aspects of your health that you can influence, such as maintaining a good sleep routine, eating nutritious food, and engaging in light exercise (with your doctor's approval). This can give you a sense of agency and empowerment.

Practice mindfulness and relaxation. Techniques like deep breathing, meditation, and yoga can help calm your nervous system when you feel anxious. Journaling is another great tool to process your emotions and reduce stress.

Remember, your feelings are a normal part of this journey. You are not alone, and there are resources available to help you navigate this fear. The key is to be proactive and seek the support you need."

**Question 3: I am an adolescent/young adult patient with metastatic melanoma and am currently receiving treatment. How will my treatment affect long-term hormone changes?**

Input 1:

"Receiving a cancer diagnosis and undergoing treatment as an adolescent or young adult can be a challenging experience, and it's natural to have questions about the long-term effects of treatment, including how it might affect your hormones. The impact of treatment on long-term hormone changes can be complex and depends on a variety of factors, including the type of treatment you receive, the specific drugs used, the dose, and your age and sex at the time of treatment.

Here's a breakdown of how different treatments for metastatic melanoma can affect your endocrine system:

1. Immunotherapy

Immunotherapy, particularly immune checkpoint inhibitors, has revolutionized the treatment of metastatic melanoma. While these drugs can be highly effective, they can also cause the immune system to attack healthy tissues and organs, including those that produce hormones. This is known as an immune-related adverse event (irAE).

Thyroid Dysfunction: Thyroid issues, such as hypothyroidism (underactive thyroid) or hyperthyroidism (overactive thyroid), are some of the most common endocrine side effects of immunotherapy. These conditions may develop months after treatment begins and can be permanent, requiring lifelong hormone replacement medication.

Hypophysitis: This is an inflammation of the pituitary gland, a small gland at the base of your brain that controls the function of many other hormone-producing glands. Hypophysitis can lead to deficiencies in several hormones, including those that regulate thyroid function, adrenal function, and sex hormones (testosterone and estrogen).

Adrenal Insufficiency: This occurs when the adrenal glands, which produce hormones like cortisol, are damaged. It can lead to fatigue, weight loss, and low blood pressure.

Diabetes: In rare cases, immunotherapy can cause type 1 diabetes, requiring lifelong insulin therapy.

2. Targeted Therapy

Targeted therapies, such as BRAF and MEK inhibitors, are another treatment option for melanoma. While these drugs are more targeted to cancer cells, they can also have side effects, and some research suggests a potential link between these treatments and changes in hormone signaling, particularly in males. Studies have shown that these therapies may cause an increase in androgen receptor levels in tumors, which could contribute to treatment resistance.

3. Traditional Treatments (Chemotherapy and Radiation Therapy)

While newer therapies are now standard for metastatic melanoma, some patients may still receive older treatments.

Chemotherapy: Certain chemotherapy drugs can affect the reproductive system, potentially leading to fertility problems and changes in sex hormone levels. The risk is often higher when treatment is given during or after puberty.

Radiation Therapy: Radiation to areas of the body that contain endocrine glands, such as the head, neck, or pelvis, can cause permanent damage. For example, radiation to the head and neck can harm the thyroid gland, leading to hypothyroidism. Radiation to the pelvic area can affect the ovaries in females or the testes in males, potentially impacting fertility and sex hormone production.

Long-Term Monitoring and Management

Because of these potential long-term effects, it is crucial to have regular, long-term follow-up care with your medical team. This may include:

Regular Blood Tests: Your doctor will likely monitor your hormone levels, including thyroid hormones, cortisol, and sex hormones, both during and after treatment.

Specialized Care: You may be referred to an endocrinologist, a doctor who specializes in hormone disorders, to help manage any long-term endocrine complications.

Fertility Preservation: If you are a young adult, it is important to discuss fertility preservation options with your doctor before starting treatment, as some treatments can cause permanent infertility.

Ongoing Communication: It's essential to communicate openly with your healthcare team about any new or persistent symptoms you experience, such as fatigue, weight changes, or changes in mood, as these could be signs of a hormonal imbalance.

While the prospect of long-term side effects can be concerning, the good news is that many of these conditions can be managed effectively with medication. With proper monitoring and care, you can maintain your quality of life and health for years to come.

Sources"

Input 2:

"This is an important question, and it's good that you're thinking about the potential long-term effects of your treatment. The impact of cancer treatment on hormones is a complex topic, and it's essential to have a frank and open conversation with your healthcare team. They are the best resource to provide you with personalized information based on your specific treatment plan, your age, and your overall health.

Here is a general overview of how different types of metastatic melanoma treatments can affect your hormones in the long term.

1. Immunotherapy

Immunotherapy, a common treatment for metastatic melanoma, works by ""unleashing"" your immune system to fight cancer cells. However, this heightened immune response can sometimes mistakenly attack healthy organs, including those in your endocrine system that produce hormones. This is known as an immune-related adverse event (irAE).

Thyroid problems: This is one of the most common hormonal side effects of immunotherapy. You may experience either an overactive thyroid (hyperthyroidism) or an underactive thyroid (hypothyroidism). While hyperthyroidism can sometimes be temporary, hypothyroidism is often permanent and requires lifelong hormone replacement medication.

Pituitary gland issues (hypophysitis): The pituitary gland is a master gland that controls other hormone-producing glands. Inflammation of the pituitary gland can lead to a deficiency in various hormones, including those that regulate thyroid function, adrenal function, and sex hormones. This can lead to fatigue, headaches, and other symptoms. Pituitary issues often require lifelong hormone replacement.

Adrenal insufficiency: The adrenal glands produce cortisol, a hormone essential for managing stress, regulating blood pressure, and other vital functions. Immunotherapy can cause these glands to become inflamed and stop working correctly, leading to a condition that can be life-threatening if not managed with steroid medication.

Diabetes: In some cases, immunotherapy can lead to a form of diabetes that resembles Type 1 diabetes, where the immune system attacks the pancreas's insulin-producing cells. This typically requires lifelong insulin replacement.

2. Targeted Therapy

Targeted therapy drugs work by targeting specific genes or proteins that help cancer cells grow and survive. While they can be very effective, they can also have side effects. Research has shown that some targeted therapies used for melanoma can influence hormone pathways.

Sex hormone signaling: One study found that targeted therapies that inhibit BRAF and MEK proteins may affect the androgen receptor, a protein that plays a role in sex hormone signaling, particularly in males. This can potentially influence treatment response and may have other long-term effects.

3. Chemotherapy

While less common for metastatic melanoma than immunotherapy and targeted therapy, chemotherapy may still be used in certain situations. The specific long-term hormonal effects of chemotherapy depend on the type of drugs, dose, and duration of treatment.

Fertility: Certain chemotherapy drugs can affect the ovaries in females and the testes in males, potentially impacting fertility and causing changes in sex hormone levels. In young women, this can lead to irregular or absent periods and symptoms of menopause. In young men, it can affect testosterone production and sperm count. The effects can be temporary or permanent.

Other hormonal changes: Some chemo drugs, along with radiation therapy, can damage organs like the thyroid, leading to long-term issues.

What to Do

The key to managing these potential long-term effects is proactive care and open communication with your medical team. Here's what you can do:

Discuss your concerns: Talk to your oncologist, endocrinologist, or other members of your care team about your specific worries. They can explain the potential risks associated with your treatment plan and what to watch out for.

Regular monitoring: Your doctors will likely perform regular blood tests to monitor your hormone levels (e.g., thyroid function, blood sugar). This helps them catch any hormonal changes early, which is crucial for effective management.

Symptom awareness: Be aware of the signs and symptoms of hormonal changes, such as fatigue, weight changes, mood swings, changes in body temperature sensitivity, and changes in sexual function. Report any unusual or persistent symptoms to your healthcare team promptly.

Lifelong follow-up care: As an adolescent/young adult patient, you will transition into survivorship care. It's vital to have a long-term follow-up plan that includes regular check-ups with a health professional who understands your risk for late effects from cancer treatment.

Remember, your health is a partnership between you and your medical team. By staying informed and communicating openly, you can work together to manage these potential long-term effects and maintain the best possible quality of life."

Input 3:

"For an adolescent or young adult patient with metastatic melanoma, the long-term effects of treatment on your hormone system are a very important consideration. The specific effects you may experience will depend on the types of treatment you receive, your sex, your age at the time of treatment, and other individual factors.

It's crucial to have an open conversation with your healthcare team about these potential long-term effects, as they can help you understand your specific risks and create a plan for monitoring and managing them. Here is a general overview of how different treatments can affect your hormones:

Immunotherapy and Hormone Changes

Immunotherapy, a common treatment for metastatic melanoma, works by activating your immune system to fight cancer cells. However, this can sometimes lead to a side effect called ""immune-related adverse events"" (irAEs), where your immune system also attacks healthy organs, including those in your endocrine system.

Thyroid problems: The most common endocrine issue from immunotherapy is thyroid dysfunction. This can be either an overactive thyroid (hyperthyroidism) or an underactive thyroid (hypothyroidism). While hyperthyroidism can cause weight loss, a fast heart rate, and anxiety, hypothyroidism can lead to fatigue, weight gain, and feeling cold. Thyroid issues often require lifelong medication to manage.

Hypophysitis: This is an inflammation of the pituitary gland, a small gland at the base of your brain that controls other endocrine glands. Hypophysitis can affect the production of several hormones, including those that regulate thyroid function, adrenal function, and sex hormones (estrogen and testosterone).

Adrenal insufficiency: The adrenal glands produce hormones like cortisol, which helps your body respond to stress. Immunotherapy can sometimes cause these glands to stop working properly, leading to weakness, fatigue, dizziness, and low blood pressure. This may also require lifelong hormone replacement therapy.

Diabetes: In rare cases, immunotherapy can cause the pancreas to stop producing insulin, leading to type 1 diabetes. This would require ongoing management with insulin.

Targeted Therapy, Chemotherapy, and Radiation

While immunotherapy is a primary concern for long-term endocrine issues, other treatments can also have an impact.

Targeted therapy: This treatment uses drugs that target specific genetic mutations in cancer cells. Side effects can include fatigue and muscle pain, but they are not as commonly associated with endocrine problems as immunotherapy.

Chemotherapy: The specific effects of chemotherapy depend on the drugs used, but high-dose chemotherapy can sometimes affect hormone-producing organs.

Radiation therapy: If radiation is used on areas of the body that contain endocrine glands (such as the head and neck, which have the thyroid, or the pelvis, which has the ovaries or testes), it can cause long-term damage to these glands. This can lead to hypothyroidism or issues with sex hormone production and fertility.

Impact on Sex Hormones and Fertility

For adolescents and young adults, the effect on sex hormones is a particularly important concern.

Female patients: Treatments that affect the ovaries can lead to early menopause-like symptoms, such as hot flashes and bone loss. It can also cause irregular periods or a complete stop to menstruation. These effects may be temporary or permanent.

Male patients: Treatments affecting the testes can reduce testosterone levels, which can lead to hot flashes, changes in libido, bone loss, and weight gain.

Fertility: Any treatment that affects the reproductive system can cause fertility problems. These can be short-term or permanent, and the risk depends on the specific treatment and your age. It is vital to discuss fertility preservation options with your care team before beginning treatment if you are interested in having children in the future.

Monitoring and Management

Because these hormonal changes can be permanent, lifelong monitoring is often necessary. Your doctors will likely perform regular blood tests to check your hormone levels. If an imbalance is detected, hormone replacement therapy can often be used to manage the symptoms and ensure a good quality of life.

It's important to remember that while the potential for long-term side effects is a serious concern, these treatments have also dramatically improved survival rates for metastatic melanoma. Your healthcare team's role is to help you navigate these issues and provide the best possible care, both for your cancer and for your long-term health."

Input 4:

"Treatments for metastatic melanoma can have a significant impact on an adolescent or young adult's endocrine system, which is responsible for producing and regulating hormones. These hormonal changes, known as ""late effects,"" can appear months or even years after treatment has ended and may be a source of ongoing concern.

Here’s a breakdown of how different treatments for melanoma can affect your long-term hormone levels and what to expect:

Immunotherapy

Immunotherapy, a common treatment for metastatic melanoma, works by activating your immune system to attack cancer cells. However, in some cases, this can lead to the immune system attacking healthy, hormone-producing glands.

Thyroid Gland: This is one of the most common endocrine side effects of immunotherapy. The treatment can cause either an overactive thyroid (hyperthyroidism) or an underactive thyroid (hypothyroidism). Symptoms of an underactive thyroid include fatigue, weight gain, and feeling cold. An overactive thyroid may cause weight loss, nervousness, and a fast heart rate. Thyroid problems often require lifelong medication to manage.

Pituitary Gland: The pituitary is often called the ""master gland"" because it controls many other glands, including the thyroid, adrenal glands, and reproductive organs. Immunotherapy can cause inflammation of the pituitary gland, a condition called hypophysitis. This can disrupt the production of several critical hormones and may lead to fatigue, low blood pressure, and other issues.

Adrenal Glands: Immunotherapy can also affect the adrenal glands, which produce hormones like cortisol that are essential for stress response and metabolism. Adrenal insufficiency can cause weakness, dizziness, and extreme tiredness.

Pancreas: In rare cases, immunotherapy can lead to inflammation of the pancreas, which affects the production of insulin. This can result in the development of diabetes.

Chemotherapy and Radiation Therapy

While targeted therapies and immunotherapy are now more common for melanoma, chemotherapy and radiation are still used in some cases. Both can cause long-term hormonal side effects, particularly if the treatment area includes hormone-producing glands.

Gonads (Ovaries and Testes): Chemotherapy, especially with certain types of drugs, and radiation to the pelvic area can damage the ovaries or testes. In adolescent and young adult patients, this can affect pubertal development, fertility, and the long-term production of sex hormones (estrogen and testosterone).

In females: This can lead to irregular or absent menstrual periods, early menopause, and symptoms like hot flashes and bone loss.

In males: This can cause low testosterone levels, which may result in decreased sex drive, bone loss, and weight gain.

Thyroid and Pituitary Glands: Radiation therapy to the head or neck area can damage the thyroid and pituitary glands, leading to long-term hormonal problems.

Targeted Therapy

Targeted therapies, which are designed to attack specific vulnerabilities in cancer cells, generally have different side effect profiles than chemotherapy or immunotherapy. While they are less likely to cause widespread endocrine problems, they can still have an impact. Some targeted drugs can cause changes in blood sugar levels, and certain inhibitors can affect the expression of hormone receptors, which may be a factor in treatment resistance and outcomes.

What This Means for You

As a young person, the long-term hormonal effects of cancer treatment are particularly important to discuss with your healthcare team. The body's endocrine system is still developing, and any disruption can have a lasting impact on growth, puberty, fertility, and overall health.

Here are some key takeaways:

Know Your Risks: Your specific risk of long-term hormonal changes depends on the type of treatment you receive, the dosage, and the areas of your body that are treated.

Fertility Preservation: Before starting treatment, it's crucial to discuss fertility preservation options with your oncologist and a reproductive endocrinologist. This can include options like egg or sperm banking.

Follow-Up Care: You will need long-term, specialized follow-up care. Endocrine disorders are among the most common late effects for young cancer survivors, and regular screenings are essential to detect and manage them early. Your care team may monitor your hormone levels and refer you to an endocrinologist if needed.

Talk to Your Doctor: Openly discuss any new or persistent symptoms, such as fatigue, weight changes, or mood shifts, with your healthcare team. These could be signs of a hormonal imbalance that can often be effectively managed with medication."

Input 5:

"Navigating cancer treatment as a young adult is challenging, and it's completely understandable to be concerned about the long-term effects, including hormonal changes. The information below is intended to provide a general overview of how different types of melanoma treatments can affect the endocrine system. It is not a substitute for medical advice from your oncology team, who can provide personalized information based on your specific treatment plan and medical history.

Your endocrine system is a network of glands that produce hormones, which regulate a wide range of bodily functions, including growth, metabolism, and sexual development. Cancer treatments can sometimes impact these glands, leading to hormonal imbalances. The risk and type of hormonal changes you may experience depend on various factors, including the specific treatments you receive, the dosage, the areas of your body being treated, and your age at the time of treatment.

Endocrine Effects of Different Treatments

1. Immunotherapy (Immune Checkpoint Inhibitors)

Immunotherapy works by stimulating your immune system to attack cancer cells. However, this can sometimes lead to an overactive immune response that mistakenly targets healthy organs, including endocrine glands. These side effects are known as immune-related adverse events (irAEs).

Thyroid problems: The thyroid gland is a common target of irAEs. This can lead to either an overactive thyroid (hyperthyroidism) or an underactive thyroid (hypothyroidism). Symptoms of thyroid problems can include fatigue, weight changes, changes in heart rate, and intolerance to cold or heat. These conditions are often manageable with medication.

Pituitary gland inflammation (Hypophysitis): Inflammation of the pituitary gland, a small gland at the base of the brain that controls many other glands, can cause a deficiency in various hormones, including growth hormone, thyroid-stimulating hormone (TSH), and hormones that regulate the adrenal glands and reproductive system. Symptoms can include headaches, vision changes, fatigue, and loss of libido.

Adrenal insufficiency: The adrenal glands, located on top of your kidneys, produce hormones like cortisol. Adrenal insufficiency can cause fatigue, dizziness, and changes in blood pressure.

Type 1 Diabetes: In rare cases, immunotherapy can cause the immune system to attack the pancreas, leading to the development of Type 1 diabetes.

2. Targeted Therapy

Targeted therapies are drugs that specifically target certain genetic mutations in cancer cells. These treatments are often used in melanoma patients with specific gene changes, such as in the BRAF gene. Side effects can vary depending on the specific drugs used, but they can sometimes affect the endocrine system. For example, some targeted drugs have been linked to changes in the endocrine system. Your doctor will monitor you for any such changes.

3. Chemotherapy

While less commonly used for metastatic melanoma than immunotherapy and targeted therapy, chemotherapy can still be part of a treatment plan. Some chemotherapy drugs can have long-term effects on the endocrine system, particularly the reproductive system.

Effects on fertility and sex hormones: Chemotherapy, especially at high doses, can damage the ovaries in females and the testes in males. This can lead to a decrease in sex hormones (estrogen and testosterone, respectively) and may cause infertility. In females, this can lead to premature menopause, with symptoms like hot flashes, vaginal dryness, and bone loss. In males, it can cause symptoms such as hot flashes, difficulty with erections, changes in sexual desire, and bone loss.

Impact on puberty: If you are still in your adolescent years, chemotherapy can affect the onset or progression of puberty.

4. Radiation Therapy

If radiation therapy is part of your treatment, the long-term effects on hormones depend on the location being treated. Radiation to the head and neck area can affect the thyroid gland, leading to a higher risk of thyroid dysfunction or, in some cases, thyroid cancer later in life. Radiation to the pelvic area can affect the ovaries or testes, impacting fertility and hormone production.

What You Can Do

Talk to your doctor: The most important step is to have an open conversation with your oncology team. They can explain the specific risks associated with your treatment plan and what they will do to monitor for potential side effects.

Ask about fertility preservation: If fertility is a concern for you, it is crucial to discuss fertility preservation options with your doctor before starting treatment. There are various options available for both males and females.

Understand the signs and symptoms: Be aware of the potential symptoms of hormonal changes (e.g., extreme fatigue, unexplained weight changes, changes in mood, feeling too hot or cold) and report them to your care team promptly. Early detection and management are key to preventing long-term problems.

Follow-up care: Long-term follow-up care is essential for cancer survivors, especially for those who were young adults during treatment. Your doctor may recommend regular blood tests to check your hormone levels and other screenings to monitor for late effects.

Navigating treatment for metastatic melanoma is a complex journey, but you don't have to do it alone. Your healthcare team is there to support you and address any concerns you have about your health, both now and in the future."

**Question 4: I am an adolescent/young adult patient with metastatic melanoma and am currently receiving treatment. I want more information about how my genetics may or may not have impacted my diagnosis and treatment.**

Input 1:

"Understanding the role of genetics in your metastatic melanoma diagnosis and treatment is a crucial part of your care. Here's a breakdown of how genetic factors can be involved:

1. Inherited vs. Acquired Gene Mutations

There are two main types of genetic changes that can impact melanoma:

Acquired (Somatic) Mutations: These are the most common type and are not inherited. They are changes that happen in a person's lifetime, often caused by environmental factors like UV radiation from the sun or tanning beds. These mutations are only present in the cancer cells themselves and are not passed down to children.

Inherited (Germline) Mutations: These are less common but can significantly increase a person's risk of developing melanoma, often at a younger age. These mutations are passed down from a parent and are present in all of the body's cells. They account for about 5% to 10% of all melanoma cases.

2. Key Genes and Their Impact on Diagnosis

Several genes have been identified that play a role in melanoma. Some of the most significant include:

BRAF: This is one of the most common gene mutations in melanoma, found in about half of all cases. A mutated BRAF gene signals cancer cells to grow and divide uncontrollably.

CDKN2A: This is a tumor suppressor gene, and mutations in it are a primary cause of familial melanoma. This mutation is associated with a higher risk of developing multiple melanomas and other cancers, such as pancreatic cancer. People with this mutation are often diagnosed at a younger age.

MC1R: This gene is responsible for skin and hair pigmentation. Variants in this gene are more prevalent in adolescents and young adults with melanoma compared to older adults. It's often associated with fair skin, red hair, freckling, and a poor ability to tan.

NRAS: Mutations in this gene are found in about 20% of melanomas that do not have a BRAF mutation. These melanomas can be more aggressive and are more likely to have lymph node metastasis.

MITF: A specific variant of this gene, p.E318K, is considered to confer a moderate risk of developing cutaneous melanoma.

3. Genetics and Treatment: Targeted Therapy

Genetic testing of your tumor can be extremely valuable in guiding your treatment plan. This is because certain gene mutations make melanoma cells susceptible to specific medications. This approach is called targeted therapy.

BRAF and MEK Inhibitors: If your tumor has a BRAF mutation, you may be a candidate for targeted therapy with a combination of BRAF and MEK inhibitors. These drugs work by blocking the activity of the mutated proteins, which can slow or stop the growth and spread of the cancer.

C−KIT Inhibitors: Mutations in the C−KIT gene are more frequently seen in certain melanoma subtypes, such as those on the palms, soles, and under the nails (acral lentiginous melanoma). Medications that target the C−KIT protein have shown promise in clinical trials for patients with this mutation.

4. Genetic Counseling and Testing

Given your age and diagnosis, your healthcare team may have already recommended or discussed genetic testing.

Tumor Biomarker Testing: A sample of your cancer is tested for specific gene mutations (like BRAF, NRAS, and C−KIT) that can be targeted by therapy.

Germline Genetic Testing: If you have a strong family history of melanoma or were diagnosed at a young age, your doctor might suggest genetic testing on a blood sample to see if you have an inherited mutation in genes like CDKN2A. Genetic counseling is often a key part of this process to help you understand the risks and implications for you and your family.

It's important to have an open conversation with your oncology team about any genetic testing that has been done, what the results mean, and how they are being used to tailor your treatment."

Input 2:

"Understanding how genetics can impact a melanoma diagnosis and treatment is an important part of your journey. It's a complex topic, so let's break down the different ways genetics can play a role.

There are two main types of genetic changes that can be involved in melanoma: inherited (germline) mutations and acquired (somatic) mutations.

Inherited (Germline) Mutations

These are genetic changes you are born with that are present in every cell of your body. They are passed down from a parent and can increase your risk of developing melanoma and other cancers. They don't guarantee that you will get cancer, but they do make it more likely.

Familial Melanoma: While most cases of melanoma are sporadic, about 10% are hereditary. If you have a strong family history of melanoma or other specific cancers, it could be a sign of an inherited mutation.

Key Genes: The most common inherited gene mutation linked to melanoma is in the CDKN2A gene. Individuals with this mutation are at a significantly higher risk of developing melanoma, often at a younger age. This mutation is also associated with an increased risk of pancreatic cancer. Other genes like CDK4, BAP1, and MITF have also been identified in familial melanoma syndromes and can be associated with other cancers like eye melanoma, kidney cancer, and mesothelioma.

Genetic Counseling: If you or your family have a history that suggests a hereditary cancer syndrome, a genetic counselor can help you understand your risks and discuss whether genetic testing is appropriate for you and your family members. This testing can be done on a blood or saliva sample.

Acquired (Somatic) Mutations

These are genetic changes that occur in your skin cells during your lifetime, often due to environmental factors like sun exposure. They are present only in the tumor cells and are not passed down to your children. These mutations are extremely important because they can directly influence your treatment plan.

The BRAF Gene: This is the most common gene mutated in melanoma, accounting for about half of all cases. A specific mutation, called BRAF V600E, leads to uncontrolled cell growth. The good news is that there are targeted therapies, such as BRAF and MEK inhibitors, that are specifically designed to block the activity of this mutated protein. These drugs can be very effective in treating metastatic melanoma with this specific mutation.

Other Genes: Other genes can also be mutated in melanoma, including NRAS, KIT, and NF1. Similar to BRAF, the presence of these mutations can guide your doctors in selecting the most effective targeted therapy for you. For example, KIT mutations are more common in certain types of melanoma and may respond to specific targeted therapies.

Tumor Genetic Testing: Your doctors likely performed genetic testing on your tumor biopsy to look for these acquired mutations. This is a standard part of care for patients with metastatic melanoma because the results determine which treatments are most likely to work.

How this Impacts Your Treatment

Your diagnosis of metastatic melanoma at a young age makes understanding these genetic factors particularly relevant.

Treatment Decisions: The results of your tumor genetic testing are crucial for guiding your treatment. If you have a BRAF mutation, for instance, you may be a candidate for targeted therapy with BRAF and MEK inhibitors. These treatments can have a dramatic impact on the disease's progression.

Clinical Trials: For some genetic mutations, there may not be an FDA-approved drug yet, but your genetic profile might make you eligible for a clinical trial exploring new targeted therapies.

Prognosis: The specific genetic mutations in your tumor can also be associated with different clinical outcomes, which can help your medical team better understand and manage your disease.

Surveillance: If you have an inherited predisposition to melanoma, your doctors may recommend more frequent skin checks and monitoring for other associated cancers.

It is highly recommended that you talk with your oncologist or a genetic counselor. They can explain your specific genetic test results in detail, clarify which mutations were found in your tumor, and discuss what that means for your current and future treatment options. They can also address any questions you may have about inherited risk for you and your family.

Sources"

Input 3:

"I understand you are an adolescent/young adult patient with metastatic melanoma and want to know more about the role of genetics in your diagnosis and treatment. This is a very important and insightful question, as our understanding of melanoma genetics has significantly advanced in recent years and directly impacts how the disease is managed.

Genetics can be understood in two main ways when it comes to melanoma: inherited genes that increase your risk, and acquired genetic changes in the tumor itself that drive its growth.

1. Inherited Genetic Factors (Germline Mutations)

Most cases of melanoma are not inherited. However, a small percentage of cases, around 10%, are familial, meaning they run in families. This is due to inherited genetic changes (called ""germline mutations"") that increase a person's risk of developing melanoma.

CDKN2A and CDK4: The most common genes associated with familial melanoma are CDKN2A and, less frequently, CDK4. Mutations in these tumor suppressor genes disrupt the cell cycle, leading to uncontrolled cell growth. If you have a family history of melanoma, especially at a young age or with multiple family members affected, your doctor may recommend genetic counseling and testing for these genes.

MC1R: Variations in the MC1R gene are also linked to an increased risk of melanoma. This gene influences skin pigmentation, and variations are often associated with fair skin, red hair, and a higher sensitivity to UV radiation.

It's important to remember that even if you have an inherited genetic mutation, it doesn't guarantee you will get melanoma. It just means you have a higher risk. Environmental factors, especially UV radiation exposure, still play a significant role.

2. Acquired Genetic Factors (Somatic Mutations)

These are genetic changes that occur during a person's lifetime and are only present in the cancer cells, not in every cell of the body. They are not inherited from a parent and cannot be passed on to children. These ""somatic mutations"" are crucial because they can be targeted with specific therapies.

In adolescents and young adults with melanoma, certain genetic mutations are more common and have a direct impact on treatment.

BRAF Mutation: The BRAF gene is the most frequently mutated gene in cutaneous melanoma, and this mutation is particularly common in younger patients. Over 60% of melanomas in adolescents and young adults have a BRAF V600 mutation. This mutation leads to an overactive protein that signals cells to grow and divide uncontrollably. The good news is that this mutation can be targeted with specific medications.

Treatment Impact: If your tumor has a BRAF V600 mutation, you are likely eligible for targeted therapy. These drugs, known as BRAF and MEK inhibitors, specifically block the overactive proteins, causing the tumor to shrink or its growth to slow down. Using a combination of a BRAF inhibitor and a MEK inhibitor is often more effective than using either drug alone.

NRAS Mutation: Mutations in the NRAS gene are the second most common in melanoma, but they are found more frequently in older adults. These mutations can also activate pathways that lead to uncontrolled cell growth.

Treatment Impact: There are currently no approved targeted therapies that directly target the NRAS protein. However, some patients with NRAS-mutated melanoma may benefit from treatments that target the pathways activated by this mutation, such as MEK inhibitors, though these are often used in combination with other therapies.

Other Mutations: Other genes like NF1 and KIT can also be mutated in melanoma. The presence of these mutations may open up different treatment options.

How this Affects Your Care

Because the genetic profile of a melanoma tumor is so important for treatment decisions, doctors will typically perform a biopsy of the tumor and send it for genetic testing. This is often referred to as ""molecular profiling."" The results will tell your medical team if your tumor has a specific mutation, like the BRAF V600 mutation, and help them determine if targeted therapies are a suitable part of your treatment plan, in addition to other options like immunotherapy or chemotherapy.

It's important to have an open conversation with your healthcare team about the results of any genetic tests and how they are being used to guide your treatment. You can ask them about the specific mutations found in your tumor, what treatment options are available for those mutations, and how these therapies work."

Input 4:

"Thank you for your question. It's understandable that you want to learn more about the role of genetics in your diagnosis and treatment. This is a complex topic, and the information below is for educational purposes. It's crucial to discuss your specific situation with your oncology team, including your doctors, genetic counselor, and other healthcare professionals. They can provide personalized advice based on your medical history and the specific characteristics of your cancer.

Here is a general overview of how genetics can impact melanoma, especially in adolescents and young adults:

1. Acquired vs. Inherited Genetic Mutations

There are two main categories of genetic changes that can lead to cancer:

Acquired (Somatic) Mutations: These are the most common type of genetic changes in melanoma. They occur during a person's lifetime and are found only in the cancer cells, not in every cell of the body. These mutations are often caused by environmental factors like exposure to ultraviolet (UV) radiation from the sun or tanning beds, which damages the DNA in skin cells. The most common acquired mutation in melanoma is in the BRAF gene, found in about half of all cases. Other frequently affected genes include NRAS, CDKN2A, and NF1.

Inherited (Germline) Mutations: These are less common but can be very important, especially in young patients. These mutations are present in every cell of your body, as they are passed down from a parent. They can significantly increase your risk of developing melanoma and other cancers, often at an earlier age.

2. High-Risk Inherited Genetic Syndromes

If your melanoma is linked to an inherited mutation, it might be part of a hereditary syndrome. Some of these include:

Familial Atypical Multiple Mole Melanoma (FAMMM) Syndrome: This is the most well-known hereditary melanoma syndrome. It's characterized by having a large number of moles (often more than 50), some of which may be atypical, and a family history of melanoma. A significant percentage of FAMMM syndrome cases are caused by mutations in the CDKN2A gene. This gene is a tumor suppressor, meaning it normally helps control cell growth. When it's mutated, cells can grow out of control. Patients with a CDKN2A mutation also have an increased risk of other cancers, such as pancreatic cancer.

Other Genes: Other genes linked to an increased risk of melanoma include CDK4, BAP1, POT1, and MITF. Mutations in these genes can also be part of inherited cancer syndromes and may be associated with other types of tumors.

Xeroderma Pigmentosum (XP): This is a very rare genetic disorder that affects the body's ability to repair DNA damage caused by UV light. Individuals with XP have an extremely high risk of developing various skin cancers, including melanoma, at a young age.

3. How Genetics Can Impact Your Treatment

Genetic testing of your tumor can be a critical step in determining the most effective treatment plan for you.

Targeted Therapy: If your tumor has a specific mutation, such as in the BRAF gene, you may be a candidate for targeted therapy. These are drugs that specifically block the abnormal proteins produced by the mutated gene, stopping the cancer cells from growing and dividing. For example, BRAF inhibitors are used for melanomas with a BRAF mutation.

Immunotherapy: Immunotherapy drugs work by ""unleashing"" your own immune system to find and attack cancer cells. These treatments are often used for metastatic melanoma and are a standard of care, regardless of a specific genetic mutation. However, some genetic profiles may predict a better response to immunotherapy.

Clinical Trials and Research: Because you are an adolescent/young adult, your doctors may also consider clinical trials. Many of these trials are focused on precision medicine, which tailors treatment to the specific genetic characteristics of a patient's tumor. Your genetic profile may make you eligible for trials of new and innovative therapies.

4. Genetic Counseling and Testing

Given your diagnosis and age, your healthcare team may have already discussed or recommended genetic counseling. A genetic counselor can help you understand:

Whether your melanoma is likely sporadic (acquired) or familial (inherited).

The risks and benefits of genetic testing for inherited mutations.

The implications of a positive test result for you and your family members.

Screening recommendations for other cancers associated with a genetic syndrome.

Again, please remember that this information is a general overview. It is essential to have an open conversation with your healthcare team to address your specific questions and concerns. They are your best resource for understanding how genetics may have influenced your cancer and the best path forward for your treatment."

Input 5:

"Thank you for your question. It's completely understandable to want to know more about the factors that contributed to your diagnosis and how they might affect your treatment. As a young person with a metastatic melanoma diagnosis, this information is particularly relevant, and it's a topic that a lot of research is currently exploring.

Here's a breakdown of how genetics can play a role in melanoma, both in diagnosis and treatment, keeping in mind the context of an adolescent/young adult patient.

The Two Types of Genetic Changes

It's important to distinguish between two main types of genetic changes:

Inherited (Germline) Mutations: These are genetic changes that are present in every cell of your body and were passed down from a parent. They can increase your lifetime risk of developing melanoma and other cancers. In the context of melanoma, about 5-10% of cases are considered hereditary.

Acquired (Somatic) Mutations: These are genetic changes that happen within a specific cell during your lifetime. They are not inherited and are only present in the cancer cells themselves. These mutations are often caused by environmental factors, like UV radiation exposure, and they are what drive the growth of the cancer.

Genetics and Your Diagnosis

While most cases of melanoma are not inherited, a family history of melanoma, especially in young patients, is a significant risk factor. Several genes have been identified that, when mutated, can increase the risk of melanoma.

CDKN2A: This is the most well-known gene associated with familial melanoma. Mutations in this gene can significantly increase the risk of developing melanoma and are also linked to an increased risk of pancreatic cancer.

MC1R: This gene is responsible for determining skin pigmentation, and variations in it are linked to red hair, fair skin, and a higher risk of melanoma.

Other Genes: Researchers have also identified other genes like CDK4, BAP1, TERT, and POT1 that are associated with a predisposition to melanoma and other cancers.

If you have multiple family members who have had melanoma or other specific cancers, it may be a good idea to speak with your doctor about a referral to a genetic counselor. They can help you assess your risk and determine if genetic testing is appropriate.

Genetics and Your Treatment

Understanding the specific genetic makeup of your tumor (the somatic mutations) is a critical part of modern cancer care, especially for metastatic melanoma. This is often done through a process called ""biomarker testing"" or ""tumor profiling.""

Targeted Therapy: The results of this testing can reveal specific mutations that can be ""targeted"" with certain medications. For example, the BRAF mutation is one of the most common mutations found in melanoma cells. If your tumor has this mutation, you may be eligible for targeted therapies that specifically block the proteins created by this faulty gene, which can shrink or slow the cancer's growth.

Immunotherapy: Immunotherapy has revolutionized the treatment of metastatic melanoma. Some research suggests that patients with certain inherited genetic variants may have a better response to immunotherapy. Additionally, specific genetic signatures within the tumor itself can help predict how a patient will respond to immunotherapy, which can help doctors decide on the most effective treatment plan. For example, some studies have identified genes that, when highly expressed, are associated with resistance to immunotherapy.

The Bottom Line for You

As a young patient with metastatic melanoma, it's very likely that your care team has already considered some of these genetic factors.

Ask about Tumor Profiling: You can ask your doctor if they have performed genetic testing on your tumor (biomarker testing) to look for specific mutations like BRAF or others. This information is crucial for tailoring your treatment plan.

Discuss Your Family History: If you haven't already, be sure to share a detailed family history of cancer with your oncologist. If you have a strong family history, they may recommend you see a genetic counselor.

Stay Informed: The field of cancer genetics is rapidly evolving. Continue to ask your healthcare providers questions and stay informed about new developments in treatment and testing.

Navigating a cancer diagnosis is challenging, and it's empowering to understand the science behind your disease. Your healthcare team is your best resource for personalized information, so don't hesitate to ask them these questions directly.

Sources"

**Question 5: I am an adolescent/young adult patient with metastatic melanoma and am currently receiving treatment. I want more information about the long-term side effects of treatment.**

Input 1:

"Receiving a diagnosis of metastatic melanoma and undergoing treatment as an adolescent or young adult can be a difficult experience, and it's completely understandable to want to know more about the potential long-term side effects. The long-term effects you may experience will depend on the specific type of treatment you receive, such as immunotherapy, targeted therapy, or chemotherapy.

Here is a general overview of the long-term side effects associated with different types of melanoma treatments. It is crucial to discuss any concerns you have with your medical team, as they can provide the most accurate and personalized information based on your specific treatment plan and health status.

Immunotherapy

Immunotherapy, particularly immune checkpoint inhibitors, is a common and effective treatment for metastatic melanoma. These drugs work by ""taking the brakes off"" your immune system so it can recognize and attack cancer cells. While this is highly effective, it can also lead to the immune system attacking healthy organs. These are called ""immune-related adverse events.""

Long-term side effects of immunotherapy can include:

Endocrine disorders: The immune system may attack hormone-producing glands, such as the thyroid or adrenal glands. This can lead to conditions like hypothyroidism (underactive thyroid) or adrenal insufficiency, which may require long-term medication.

Gastrointestinal issues: Chronic inflammation of the colon (colitis) can cause persistent diarrhea and abdominal pain.

Organ inflammation: In rare cases, immunotherapy can cause long-term inflammation of the lungs (pneumonitis), liver (hepatitis), or heart (myocarditis).

Fatigue: Persistent and overwhelming fatigue is a common long-term side effect that can last for months or even years after treatment.

Arthritis: Joint pain and inflammation can be a long-lasting side effect.

Targeted Therapy

Targeted therapy drugs are designed to attack specific vulnerabilities in cancer cells. These are often used when the melanoma has a specific genetic mutation, like a BRAF mutation.

Possible long-term side effects of targeted therapy can include:

Skin problems: Rashes, dryness, and a thickening of the skin are common. Some people may also experience increased sensitivity to sunlight.

Cardiovascular issues: Targeted therapies can sometimes affect heart function, and your medical team may monitor this with regular tests.

Nerve damage: Some targeted therapies may lead to a condition called peripheral neuropathy, which can cause long-term numbness, tingling, or pain in the hands and feet.

Other cancers: There is a small, increased risk of developing other skin cancers (like squamous cell carcinomas) with some targeted therapies, which are usually less serious than melanoma and can be treated by removal.

Fertility issues: Many targeted therapies can cause birth defects, so it's important to discuss pregnancy plans with your doctor.

Chemotherapy

While chemotherapy is used less frequently for melanoma than immunotherapy or targeted therapy, it may be an option in certain cases.

Long-term side effects of chemotherapy can include:

Peripheral neuropathy: This can cause lasting numbness, tingling, or a burning sensation in the hands and feet.

Organ damage: Some chemotherapy drugs can cause permanent damage to the heart, lungs, kidneys, or reproductive organs. Your doctors will monitor your organ function closely.

Fertility issues: Chemotherapy can damage reproductive organs, potentially leading to long-term or permanent fertility problems. It is very important to discuss fertility preservation options with your medical team before starting treatment if this is a concern for you.

Important Considerations for Young Adults

As a young adult, you may have specific concerns related to long-term side effects that are particularly relevant to your stage of life:

Fertility: Discussing fertility preservation options with your medical team is crucial, as some treatments can affect your ability to have children in the future.

Mental health: The emotional and psychological impact of a cancer diagnosis and treatment can be significant. Long-term side effects like fatigue and chronic pain can also contribute to mental health challenges. It is important to seek support from therapists, support groups, or a mental health professional.

Overall health: Maintaining a healthy diet and lifestyle, including regular exercise as your body allows, can help manage some side effects and improve your overall well-being.

Your medical team is your best resource for understanding and managing these potential side effects. They can provide detailed information about your specific treatment plan, help you monitor for any emerging issues, and connect you with supportive care services. It is essential to communicate any new or worsening symptoms to them as soon as they appear."

Input 2:

"Navigating a cancer diagnosis and treatment as a young person can be incredibly challenging, and it's completely understandable to want to know what to expect in the long run. The long-term side effects of melanoma treatment can be varied and depend on the type of treatment you receive. Here is a general overview of potential long-term side effects associated with the most common treatments for metastatic melanoma.

Immunotherapy

Immunotherapy works by harnessing your own immune system to fight cancer cells. While this can be very effective, it can also lead to long-term side effects as the immune system may attack healthy tissues in the body. These are often referred to as ""immune-related adverse events"" (irAEs). Some of the potential long-term effects include:

Endocrine disorders: The immune system can attack hormone-producing glands, such as the thyroid, pituitary gland, and adrenal glands. This can lead to conditions like hypothyroidism (underactive thyroid) or adrenal insufficiency, which may require lifelong hormone replacement therapy.

Inflammatory conditions: Immunotherapy can cause chronic inflammation in various organs. This may lead to long-term issues like:

Colitis: Inflammation of the colon, causing diarrhea and abdominal pain.

Hepatitis: Inflammation of the liver.

Pneumonitis: Inflammation of the lungs, causing a cough or shortness of breath.

Arthritis: Joint pain and inflammation.

Vitiligo: Changes in skin pigmentation, where patches of skin lose their color. This is a common and often permanent side effect of immunotherapy for melanoma.

Other potential effects: Less common but serious long-term side effects can affect the nervous system, kidneys, or heart.

Targeted Therapy

Targeted therapy drugs work by blocking specific genes or proteins that help cancer cells grow and survive. The long-term side effects of these treatments can also be a concern.

Cardiovascular issues: Some targeted therapies can affect the heart, potentially leading to high blood pressure or other heart-related problems.

Skin problems: Long-term skin issues are common, including rash, dryness, itching, and increased sensitivity to the sun. Some patients may also experience a thickening of the skin on their palms and soles (hand-foot syndrome).

Liver and kidney problems: Targeted therapies can sometimes affect the function of the liver or kidneys, which is monitored through regular blood tests.

Increased risk of other skin cancers: In some cases, targeted therapy can increase the risk of developing new squamous cell skin cancers.

Chemotherapy

While newer treatments like immunotherapy and targeted therapy are now more common for advanced melanoma, chemotherapy may still be used in certain situations. The long-term side effects of chemotherapy are well-documented and can include:

Peripheral neuropathy: Nerve damage that can cause long-lasting pain, tingling, or numbness, especially in the hands and feet.

Organ damage: Some chemotherapy drugs can cause permanent damage to organs like the heart, kidneys, or lungs.

Infertility: Chemotherapy can affect the reproductive system, potentially leading to infertility in both males and females. If this is a concern, it's important to discuss fertility preservation options with your medical team before beginning treatment.

General Long-Term and Late Effects

Beyond the specific treatments, there are other long-term and late effects of cancer and its treatment that are particularly relevant for adolescents and young adults:

Cognitive impairment: Sometimes referred to as ""chemo brain,"" this can involve changes in memory, focus, and thinking.

Mental and emotional health: Many young cancer survivors experience anxiety, depression, post-traumatic stress, and fear of recurrence. Talking to a mental health professional or joining a support group can be incredibly helpful.

Hearing and vision changes: High doses of certain chemotherapy drugs or radiation to the head can cause permanent hearing loss or vision changes.

Increased risk of secondary cancers: While rare, some cancer treatments can slightly increase the risk of developing a new, different type of cancer years later.

It is crucial to have an open and honest conversation with your healthcare team about any side effects you are experiencing. They can provide you with personalized information, help you manage symptoms, and develop a long-term survivorship plan. A survivorship plan is a detailed document that outlines your cancer history, treatment, and recommendations for future check-ups and screenings to monitor for potential long-term effects."

Input 3:

"I understand that you are an adolescent/young adult patient with metastatic melanoma and are seeking information about the long-term side effects of your treatment. It's important to have open and honest conversations with your healthcare team about any side effects you're experiencing, as they can provide personalized guidance and management strategies. The information provided here is for educational purposes and should not replace a consultation with your doctor.

The long-term side effects of metastatic melanoma treatment can vary widely depending on the specific type of treatment you are receiving, the dosage, your overall health, and how your body responds. The two primary types of systemic therapy for metastatic melanoma are immunotherapy and targeted therapy.

Long-Term Side Effects of Immunotherapy

Immunotherapy works by ""unleashing"" your body's immune system to fight cancer cells. However, this can sometimes cause the immune system to attack healthy organs and tissues, leading to what are known as immune-related adverse events. While many of these are temporary and resolve after treatment, some can become chronic and require long-term management.

Common Chronic Side Effects:

Endocrine System Problems: Immunotherapy can affect the glands that produce hormones, leading to conditions like hypothyroidism (underactive thyroid) or adrenal insufficiency. These conditions are often managed with daily medication, but the effects may be permanent.

Musculoskeletal Issues: Joint pain (arthritis) and inflammation can be a persistent side effect.

Skin Problems: Some patients may experience ongoing skin issues like rashes, itching, or changes in skin color, such as vitiligo (loss of skin pigmentation).

Fatigue: Persistent and overwhelming fatigue is a common side effect of cancer and its treatment, and it can continue long after treatment has ended.

Gastrointestinal Issues: Chronic diarrhea or colitis (inflammation of the colon) can occur and may require ongoing management.

Other Potential Long-Term Side Effects:

Neurological Complications: Rarely, immunotherapy can cause inflammation in the nervous system, leading to symptoms like numbness, tingling, or pain.

Cardiovascular Issues: Though rare, serious heart-related side effects like inflammation of the heart muscle (myocarditis) have been reported.

Fertility Issues: Treatment for melanoma, including immunotherapy, may have an impact on fertility. This is a very important topic to discuss with your healthcare team if you are considering having children in the future.

Long-Term Side Effects of Targeted Therapy

Targeted therapy drugs work by targeting specific genetic mutations in cancer cells, such as the BRAF gene. While they are designed to be more precise than traditional chemotherapy, they can still have long-term side effects because the proteins they target are also found in healthy cells.

Common Chronic Side Effects:

Skin Changes: Rashes, dry skin, and increased sensitivity to the sun are common side effects that can persist. Some targeted therapies can also lead to the development of new, usually less serious, skin cancers.

Cardiovascular Issues: Certain targeted therapies can cause heart damage, and your doctor may monitor your heart function before, during, and after treatment.

Joint and Muscle Pain: Chronic pain in your joints or muscles can be a long-term side effect.

Fatigue: As with other cancer treatments, persistent fatigue is a common complaint.

Neurological Effects: Numbness or tingling in the hands and feet (peripheral neuropathy) can be a lasting side effect.

Psychosocial and Emotional Effects

Living with a cancer diagnosis and undergoing treatment can have a significant and lasting impact on your mental health. Studies have shown that people with melanoma are more likely to experience anxiety and depression compared to the general population. The fear of cancer recurrence and concerns about body image can also be ongoing issues. It's crucial to seek support from your healthcare team, social workers, or a mental health professional to help you navigate these challenges.

Important Things to Remember

Communication is Key: Report any new or worsening symptoms to your healthcare team immediately. Early treatment of side effects can prevent them from becoming more severe or lasting longer.

Individualized Experience: Everyone's experience with treatment and side effects is unique. Your doctor will monitor your specific situation and tailor your care accordingly.

Support Systems: Don't hesitate to lean on your friends, family, and support groups. They can be a valuable source of emotional support and practical help."

Input 4:

"Navigating cancer treatment as an adolescent or young adult can be especially challenging, and it's completely understandable to want to know more about what the future might hold. The long-term side effects of metastatic melanoma treatment can vary widely depending on the specific type of treatment you're receiving, your overall health, and how your body responds.

It is important to remember that this information is not a substitute for medical advice. You should always discuss any concerns you have with your healthcare team, as they can provide guidance tailored to your specific situation.

Here's an overview of potential long-term side effects associated with common metastatic melanoma treatments:

Immunotherapy

Immunotherapy works by boosting your immune system to fight cancer cells. Because it activates your immune system, it can sometimes cause long-term inflammation and autoimmune-like conditions that may affect healthy organs and tissues. These side effects, known as immune-related adverse events (irAEs), can sometimes persist for months or even years after treatment ends.

Potential long-term side effects of immunotherapy can include:

Endocrine Issues: The immune system can attack hormone-producing glands, leading to conditions like hypothyroidism (underactive thyroid) or adrenal insufficiency. These conditions may require lifelong hormone replacement therapy.

Musculoskeletal Problems: Some patients experience persistent joint or muscle pain, often described as a form of arthritis.

Gastrointestinal Issues: Inflammation of the colon (colitis) can lead to chronic diarrhea and other digestive problems.

Skin and Hair Changes: You may experience long-lasting skin rashes, itching, or vitiligo (loss of skin color). In some cases, hair loss or changes in hair growth may be long-term.

Neurological Issues: Although less common, immunotherapy can cause inflammation of the brain or nervous system, which may result in a variety of symptoms, including headaches, numbness, or tingling.

Eye Problems: Some people may develop eye conditions like uveitis, which is inflammation of the eye's middle layer. This can cause blurry vision, pain, or light sensitivity and may require long-term management with an ophthalmologist.

Targeted Therapy

Targeted therapies work by specifically targeting certain genes or proteins in cancer cells. These treatments can also affect healthy cells that have similar characteristics, leading to side effects. While many side effects are temporary and resolve after treatment, some can become long-term.

Potential long-term side effects of targeted therapy can include:

Skin, Hair, and Nail Changes: This is a very common side effect. You may experience persistent acne-like rashes, dry skin, or hand-foot syndrome (redness, pain, and blistering on the palms and soles). Long-term use can sometimes lead to hair loss, or changes in hair growth and color.

Cardiovascular Issues: Certain targeted therapies can cause high blood pressure or damage the heart. Your healthcare team will monitor your heart function closely before, during, and after treatment.

New Skin Cancers: Some targeted therapies may increase the risk of developing new skin cancers, such as squamous cell carcinoma. Regular skin checks by a dermatologist are crucial.

Slow Wound Healing: Problems with wound healing and blood clotting can sometimes persist after treatment.

General and Psychosocial Side Effects

Regardless of the specific treatment, cancer and its treatment can have a significant long-term impact on your overall health and well-being.

""Chemo Brain"" or Cognitive Impairment: This term refers to changes in memory, thinking, and focus that can be caused by various cancer treatments. While it's often associated with chemotherapy, other treatments can also contribute to this.

Fatigue: Persistent tiredness and weakness are common long-term side effects for many cancer survivors.

Mental and Emotional Distress: Young adults with a cancer diagnosis are at a higher risk for mental health challenges, including anxiety, depression, post-traumatic stress disorder (PTSD), and fear of recurrence. These are all valid and important feelings to address.

Fertility Issues: Some cancer treatments can affect fertility. It's important to talk to your doctor about this possibility and any options you may have for preserving fertility if you are interested in having children in the future.

Your healthcare team is your best resource for managing these concerns. They can provide you with a survivorship care plan that includes recommendations for long-term monitoring, screenings, and management of any ongoing side effects. Please don't hesitate to share all of your concerns with them."

Input 5:

"Thank you for reaching out with this important question. It's completely understandable that you're seeking more information about the long-term side effects of your treatment. As a young adult patient, you have specific concerns that your care team should address. It's crucial to have open and honest conversations with your doctors and nurses about what to expect, as they can provide you with the most accurate and personalized information based on your specific treatment plan, overall health, and the type of metastatic melanoma you have.

The long-term side effects of treatment for metastatic melanoma can vary greatly depending on the type of therapy you are receiving. The main types of systemic treatments are immunotherapy, targeted therapy, and chemotherapy. Here is a general overview of the potential long-term effects associated with each:

Long-Term Side Effects of Immunotherapy

Immunotherapy, particularly with checkpoint inhibitors, is a common and effective treatment for metastatic melanoma. It works by ""unleashing"" your immune system to fight cancer, but sometimes this can lead to an overactive immune response that affects healthy organs. These are known as immune-related adverse events (irAEs). While many are temporary, some can become long-term or permanent.

Endocrine Problems: Your immune system might attack your hormone-producing glands. The most common is a change in thyroid function, but it can also affect the pituitary gland or adrenal glands. These conditions often require long-term hormone replacement medication.

Inflammation in Organs: Immunotherapy can cause inflammation (colitis) in the colon, leading to long-term diarrhea or gastrointestinal issues. It can also cause inflammation in the liver (hepatitis), lungs (pneumonitis), or other organs, which may require ongoing management.

Fatigue: Many patients experience significant, long-lasting fatigue that is different from simple tiredness. It's a common and sometimes debilitating side effect that can persist long after treatment ends.

Rheumatological Issues: Some people may develop long-term joint pain, swelling, and other arthritis-like symptoms.

Long-Term Side Effects of Targeted Therapy

Targeted therapies are drugs that specifically attack cancer cells with certain genetic mutations, like the BRAF mutation in melanoma. These treatments often come with their own set of potential long-term side effects.

Skin Problems: This is a very common side effect. It can include persistent rashes, dry skin, and increased sensitivity to the sun. Some patients may also develop new squamous cell skin cancers.

Heart Problems: Some targeted therapies can affect heart function, and your doctor will likely monitor your heart health before, during, and after treatment.

Other Potential Effects: Long-term use can sometimes lead to issues like fever, joint pain, muscle pain, and an increased risk of developing other types of cancer, though this risk is usually small.

Long-Term Side Effects of Chemotherapy

While less common now for first-line treatment of metastatic melanoma, chemotherapy is still used in some cases. The long-term effects are often related to the damage chemo drugs can cause to healthy, fast-growing cells.

Nerve Damage (Peripheral Neuropathy): This is a serious side effect of some chemo drugs that can cause long-term or permanent tingling, numbness, or pain in your hands and feet.

Organ Damage: In rare cases, chemotherapy can cause permanent damage to organs like the heart, lungs, kidneys, or reproductive organs. Your doctors should be monitoring your organ function closely.

Fertility Issues: Chemotherapy can damage reproductive organs and may cause long-term or permanent infertility. This is a particularly important issue for young adults, and it's essential to discuss fertility preservation options with your care team before starting treatment.

Cognitive Changes (""Chemo Brain""): Some patients report long-term problems with memory and concentration after chemotherapy.

Psychosocial and Quality of Life Concerns

Beyond the physical side effects, living with metastatic melanoma as a young adult presents unique challenges:

Mental Health: Studies have shown that people with melanoma, especially young adults, have a higher prevalence of anxiety, depression, and fear of cancer recurrence. It's crucial to seek support from mental health professionals who specialize in oncology.

Social and Developmental Challenges: As a young adult, you are at a critical time for developing your identity, career, and relationships. Cancer can interrupt these plans and make it difficult to navigate social situations, dating, and professional goals.

Body Image: Changes to your body from surgery, scars, or treatment side effects can affect your self-esteem and body image.

Survivorship Care Plans: It's a good idea to work with your healthcare team to create a survivorship care plan. This is a document that summarizes your treatment, outlines a schedule for follow-up exams, and lists potential long-term side effects to watch for. It can be a valuable tool for managing your health in the years to come.

Remember, early communication with your healthcare team is key. If you experience any new or worsening symptoms, even if they seem minor, you should report them immediately. Your doctors can often manage side effects more effectively if they are caught early.

Sources"
