# Supplementary material for: Quality, Empathy, and Readability of AI Chatbot Responses to the Survivorship Needs of Adolescents and Young Adults With Melanoma: Evaluation Study
Source: JMIR Cancer. 2026 Mar 26;12:e84234. doi: 10.2196/84234 (PMC13020680; doi:10.2196/84234)
Supplement: Multimedia Appendix 7 [file cancer-v12-e84234-s007.docx]

Chat GPT

On Treatment Questions

**Question 1: I am an adolescent/young adult patient with metastatic melanoma and am currently receiving treatment. I want more information about what will happen when treatment finishes.**

Chat GPT

**Input 1:**

I'm really glad you reached out—navigating life during and after treatment for metastatic melanoma can bring a lot of questions and uncertainty. While every person’s experience is different, here’s a general overview of what to expect when your treatment ends:
---
### 🔄 **What Happens After Treatment for Metastatic Melanoma?**
#### 1. **Monitoring and Follow-Up Care**
Even after active treatment ends, your medical team will continue to monitor you closely. This typically includes:
* **Regular scans (CT, PET, or MRI)** to check for recurrence or progression.
* **Blood tests** (such as LDH levels).
* **Physical exams** every few months at first, then less frequently over time.
**Frequency:**
* Every 3–6 months for the first 2–3 years
* Then every 6–12 months, depending on your case
> ✅ Tip: Keep a personal calendar of follow-up visits and tests.
---
#### 2. **Long-Term or Maintenance Therapy**
Some people with metastatic melanoma remain on **immunotherapy (like nivolumab or pembrolizumab)** or **targeted therapy (for BRAF mutations)** long term, either:
* Because it's working well, or
* As a maintenance strategy to keep the cancer from returning
Your oncologist will guide if continued treatment is needed or if a break is safe.
---
#### 3. **Side Effects: Managing the Long-Term Impact**
Even after treatment ends, some side effects may continue, including:
* **Fatigue**
* **Skin changes or scarring**
* **Lymphedema** (if lymph nodes were removed)
* **Immune-related issues** (from immunotherapy)
Be open with your care team about any symptoms, even months after stopping treatment.
---
#### 4. **Emotional Health & Adjustment**
Ending treatment can be unexpectedly emotional. You might feel:
* **Relief** that it's over
* **Anxiety** about recurrence
* **Disconnection** from your care team or support system
**Support Options:**

* AYA (Adolescent and Young Adult) oncology programs
* Cancer support groups (online or in-person)
* Counseling or therapy (many cancer centers offer this)
---
#### 5. **Survivorship Planning**
Your care team may provide a **"survivorship care plan"** that includes:
* Your treatment summary
* What follow-up care you need
* Signs to watch for
* Tips for wellness (exercise, nutrition, skin protection, etc.)
> 🧴 **Protect your skin** long-term: Avoid sunburns, wear SPF daily, and do regular self-skin checks.
---
#### 6. **Fertility, Career, and Life Goals**
As a young person, you may also need guidance about:
* **Fertility options** (if affected by treatment)
* **Education or career planning**
* **Dating and relationships**
* **Transitioning from pediatric/AYA care to adult oncology**
Don’t hesitate to ask your care team for referrals to specialists who can help.
---
### 💬 You Might Ask Your Team:
* “What signs should I watch for that might mean the cancer is coming back?”
* “What should I expect in terms of recovery or side effects long-term?”
* “How often will I have scans or follow-up appointments?”
* “Are there survivorship resources or AYA support groups I can connect with?”
---
Would you like help creating a list of specific questions to ask at your next appointment? Or information about survivorship programs near you?

**Input 2:**

I'm really glad you reached out—navigating life after treatment for metastatic melanoma can bring a lot of questions and emotions. Here’s a clear breakdown of what to expect **after treatment finishes**, with a focus on your age group (adolescents and young adults), and what support and follow-up usually look like.
---
### 🧠 What Happens After Treatment for Metastatic Melanoma?
**1. Regular Follow-Up Appointments**
Even when treatment ends, your care doesn’t stop. You'll likely have:
* **Scans (like CT, PET, or MRI)** every few months (e.g., every 3–6 months at first).
* **Blood tests** to check for signs of recurrence or side effects.
* **Physical exams** to watch for any symptoms.
These visits are to:
* Monitor for recurrence.
* Manage any side effects or complications from treatment.
* Support your overall health (mental and physical).
---
**2. Long-Term Side Effects and Recovery**
Treatments like immunotherapy or targeted therapy can cause:
* **Fatigue** (very common, and may last a while).
* **Hormonal changes** (some treatments affect your thyroid or adrenal glands).
* **Skin issues** (rashes, dryness, sensitivity).
* **Neuropathy** or joint pain, depending on the drugs used.
Sometimes these effects continue even after treatment ends. Your team can help you manage them.
---
**3. Mental and Emotional Health**
Ending treatment can feel like a strange transition. Many young patients feel:
* **Relief**, but also **anxiety** about “what now?”
* **Fear of recurrence** is very common.
* **Disconnection** from the medical team, now that you're not seeing them as often.
* **Isolation** if friends/family don’t fully understand your experience.
> Talking to a **therapist** or **AYA oncology social worker**, or joining a **young adult cancer support group**, can be incredibly helpful.
---
**4. Lifestyle and Health Changes**
* **Sun protection** is critical — your skin will always be more vulnerable.
* **Nutrition and exercise** can improve energy, mood, and recovery.
* You may need **vaccinations**, especially if you were immunosuppressed during treatment.
* **Fertility discussions** (if relevant) should continue if they were impacted by treatment.
---
**5. Career, School, and Life Planning**
You may be thinking about:
* Returning to school or work.
* Taking time off or changing paths.
* Finding purpose or redefining goals.
A **survivorship care plan** can help—this is a document your team can create outlining:
* Your treatment history.
* Your ongoing needs.
* Recommendations for care and life planning.
---
**6. What If the Cancer Doesn’t Go Away Completely?**
For some people, melanoma becomes more of a **chronic illness** than something that can be completely cured. In those cases:
* You might have **maintenance therapy** or **periodic treatments**.
* The goal is to keep the cancer controlled and preserve quality of life.
* Your doctors will talk with you about the best strategies and options available.
---
### 🧭 Resources to Help You Navigate
* **Stupid Cancer** – Support and info for AYA cancer patients: [<https://stupidcancer.org/](https://stupidcancer.org/>)
* **Melanoma Research Foundation** – Patient support, treatment updates: [<https://melanoma.org/](https://melanoma.org/>)
* **Imerman Angels** – 1-on-1 peer support with other young cancer survivors: [<https://imermanangels.org/](https://imermanangels.org/>)
* **CancerCare** – Free counseling and financial support services: [<https://cancercare.org/](https://cancercare.org/>)
---
Would you like help **creating a list of questions** to bring to your care team for your next visit? Or would you like to talk more about **life after melanoma** from an emotional or practical perspective?

**Input 3:**

I'm really glad you reached out with this question — it's an important one. Finishing treatment for metastatic melanoma is a major milestone, but it can also bring up a lot of uncertainty about what comes next. Here’s an overview of what typically happens after treatment ends, tailored for adolescent and young adult (AYA) patients:
---
## 🌅 What Happens After Treatment for Metastatic Melanoma?
### 1. **Follow-Up Care and Monitoring**
After active treatment finishes, you’ll usually enter a **surveillance or follow-up phase**. This means your care team will closely monitor you for:
* **Signs of recurrence** (if the cancer comes back)
* **New cancers or health issues** related to treatment
* **Ongoing side effects** or late effects from the drugs (especially immunotherapy or targeted therapy)

**This may involve:**
* Physical exams every few months (often every 3–6 months at first)
* Regular imaging (CT, PET, or MRI scans)
* Blood tests
* Skin checks (for new or recurring melanomas)

### 2. **Managing Side Effects (Some May Be Long-Term)**
Even after treatment ends, **side effects may continue** or develop later (called "late effects"). These can include:
* **Fatigue**
* **Hormonal issues** (especially if immunotherapy affected your thyroid or adrenal glands)
* **Skin changes**
* **Neuropathy** (numbness or tingling)
* Emotional effects like **anxiety** or **depression**
You'll likely still need support from specialists like:
* Dermatologists
* Endocrinologists
* Mental health professionals
* Fertility experts (if treatment may have affected fertility)

### 3. **Life After Cancer: Emotional and Social Challenges**
Many AYA patients say that **life after treatment** can be one of the hardest parts. You might feel:
* **Anxious about the cancer coming back**
* **Isolated** if friends/family don’t quite understand what you’ve been through
* **Confused about identity**, goals, or next steps (like school, work, relationships)

These feelings are valid, and it’s okay to ask for help. Talking with a therapist, joining a support group, or connecting with an AYA oncology program can make a big difference.
### 4. **Survivorship Care Plan**
Before or soon after finishing treatment, your care team should give you a **survivorship care plan**, which includes:
* A summary of the treatment you received
* A schedule for follow-up visits and tests
* Signs to watch for (symptoms of recurrence or late effects)
* Healthy lifestyle advice (nutrition, sun protection, exercise, mental health)

You can take this to your primary care doctor or any new healthcare provider in the future.

### 5. **Clinical Trials or Maintenance Therapy**
In some cases, your doctor might recommend:
* **Maintenance treatment** (to reduce risk of recurrence)
* **Participation in clinical trials** (especially if you're high-risk or want to explore new options)
Ask your oncologist if any of these apply to you.
---
## 💬 Tips Just for AYAs

* **Ask questions** — Your care team wants to help you plan for the future.
* **Stay connected** — Peer support can help you feel less alone.
* **Plan your goals** — Whether it’s college, work, travel, or relationships, this is your time to start rebuilding or redefining your future.
* **Protect your skin** — You’ll need to be vigilant about sun exposure for the rest of your life.
---
Would you like help creating a personalized checklist or questions to ask your doctor before your treatment ends? Or maybe recommendations for AYA support programs or resources?

**Input 4:**

I'm really glad you reached out — that’s a very important question, and it’s completely understandable to want clarity about what comes next. While I can’t give you specific medical advice, I can provide a general overview of what may happen after treatment finishes for **metastatic melanoma** in **adolescents and young adults (AYA)** like yourself. The journey after treatment can vary a lot depending on your individual situation, including your response to treatment, the type of treatment you’ve received, and your overall health.
---
### 🔍 After Treatment for Metastatic Melanoma: What to Expect
#### 1. **Surveillance and Follow-up Care**
Once treatment ends, most patients move into a **surveillance or follow-up phase**. This typically includes:
* **Regular check-ups** with your oncology team
* **Imaging scans** (e.g., CT, PET, or MRI) at scheduled intervals to monitor for recurrence or progression
* **Blood tests**, sometimes including LDH levels
* Skin checks to monitor for new melanomas

💡 *Follow-up frequency usually starts every 3–6 months, then may become less frequent if things remain stable.*
---
#### 2. **Managing Long-Term or Late Effects**
Depending on the treatment(s) you've received — such as **immunotherapy (like checkpoint inhibitors)** or **targeted therapy (like BRAF/MEK inhibitors)** — you may experience some **long-term or delayed side effects**, such as:
* Fatigue
* Skin issues (rash, vitiligo, dryness)
* Hormonal imbalances (especially if immunotherapy affected endocrine glands)
* Nerve or joint problems
* Mental health impacts like anxiety or depression

You might be referred to specialists (like an endocrinologist, dermatologist, or psychologist) to manage these.
---
#### 3. **Transitioning Back to "Normal" Life**
This can be surprisingly challenging. Many AYAs talk about feeling:
* Uncertain or anxious without regular treatment
* Isolated from peers who haven’t been through cancer
* Pressured to "bounce back" quickly

You might find it helpful to:
* Work with an AYA survivorship program (many cancer centers have them)
* Connect with AYA cancer support groups (like Stupid Cancer, Elephants and Tea, or Canteen)
* Ask about **survivorship care plans** — a written summary of your treatment and a plan for monitoring
---
#### 4. **Risk of Recurrence or Progression**
Even after active treatment ends, melanoma can recur — especially metastatic melanoma. Your doctor will talk with you about:
* The current **status of your disease** (complete remission, stable disease, partial response, etc.)
* The risk of **relapse or spread**
* What signs/symptoms to watch for

Having a plan in place for what happens **if** the cancer returns can actually reduce anxiety for many people.
---
#### 5. **Clinical Trials and Maintenance Therapy**
In some cases, your doctor might recommend:
* **Ongoing immunotherapy** as a maintenance approach
* **Enrolling in a clinical trial** for long-term monitoring or new treatments
* **Observation only**, if there's no evidence of disease
---
#### 6. **Fertility, Career, and Life Planning**
As a young adult, you might also want to talk about:
* Fertility (if that hasn’t already been addressed)
* Returning to work/school
* Insurance and financial planning
* Long-term emotional well-being

There are social workers, AYA navigators, and survivorship counselors who can help.
---
### 🧭 Questions to Ask Your Oncology Team
Here are a few questions you might want to bring up at your next appointment:

* “What does my follow-up care look like?”
* “What symptoms should I be watching out for?”
* “Are there any late side effects I should be aware of?”
* “What’s my current disease status?”
* “Who can I talk to about mental health or support groups?”
* “Is there a survivorship program here?”
---
Would you like a printable **checklist of post-treatment questions** or a **personalized survivorship care plan template**? I can create one for you.

And if you tell me more about your specific treatment (e.g., immunotherapy, BRAF/MEK inhibitors, etc.), I can tailor this more closely to your experience.

**Input 5:**

Thank you for sharing that — navigating metastatic melanoma and its treatment is a serious challenge, and it’s completely understandable to want to know what to expect when treatment finishes. Here’s a general overview of what typically happens next, tailored for adolescent and young adult (AYA) patients:
---
## 🧭 What Happens After Treatment for Metastatic Melanoma?
### 1. **Follow-Up and Monitoring (Surveillance)**
Even after active treatment ends, your medical team will continue to monitor you closely. This typically involves:
 **Regular scans (CT, PET, or MRI)** to check for recurrence or spread
* **Blood tests**, including tumor markers (like LDH)
* **Physical exams** and skin checks
* **Frequency**: Every 3–6 months for the first couple of years, then less frequently if you're stable

> 🔄 Surveillance continues even if you're feeling well — melanoma can recur months or even years after treatment.
---
### 2. **Managing Long-Term Side Effects**
Immunotherapy, targeted therapy, and other treatments can cause long-term or late-onset side effects, such as:
* **Fatigue**
* **Joint pain**
* **Hormone issues** (especially if the pituitary gland is affected by immunotherapy)
* **Skin problems**
* **Cognitive or mood changes**
💡 Your care team may include specialists like endocrinologists, dermatologists, or psychologists to help manage these.

---
### 3. **Emotional & Mental Health Support**
Finishing treatment can bring a mix of emotions:
* Relief that treatment is done
* Anxiety about the cancer returning
* Feeling "lost" without regular contact with your care team
You’re not alone in this. Many AYA patients experience these feelings. Counseling, peer support groups, or survivorship programs can help.
---

### 4. **Lifestyle and Wellness**
Your oncologist may encourage:

* **Sun protection** (critical for melanoma survivors)
* **Healthy diet and exercise**
* **Mental health care**
* **Avoiding smoking and excess alcohol**
* **Skin self-exams** monthly

These help reduce the risk of recurrence and improve overall well-being.
---
### 5. **Returning to School, Work, or Normal Life**
You might want (or need) to resume:
* **School or university**
* **Work**
* **Social life and dating**
This transition can be tricky. Ask your team if there’s an AYA care navigator or social worker to help ease this shift and advocate for accommodations if needed.

---
### 6. **If the Treatment Wasn’t Curative**
If your melanoma isn’t curable but is under control, treatment might shift to a **maintenance** or **chronic disease management** model. This might include:

* Ongoing low-dose treatment
* Regular check-ins to monitor progression
* Quality of life-focused care
In some cases, **clinical trials** may be available if standard treatments no longer work.

---
## 👥 Survivorship Care Plan
Ask your oncology team for a **Survivorship Care Plan**. This usually includes:

* A summary of your treatments
* A follow-up schedule
* A list of possible late effects to watch for
* Info for your primary care provider (PCP)

This helps ensure smooth communication as you transition out of cancer treatment.
---
Would you like help preparing questions for your next appointment or finding AYA-specific support groups or resources?

**Question 2: I am an adolescent/young adult patient with metastatic melanoma and am currently receiving treatment. In the last month I have become more worried about my cancer spreading. What do I need to know and what steps can I take to cope with this fear?**

Input 1:

I'm really sorry you’re going through this. Facing metastatic melanoma as an adolescent or young adult is incredibly tough—not just physically, but emotionally and psychologically as well. It's completely understandable that your fear of the cancer spreading has intensified, especially as you go through treatment. Here’s what you need to know, and some practical steps you can take to manage that fear:

---
## **What You Need to Know**
### 1. **Fear of Progression is Normal**
* It's called **Fear of Cancer Progression (FoP)**, and it's very common in patients with metastatic cancers—especially for younger people who often feel "out of sync" with peers.
* You're not alone, and your fear doesn't mean you're "weak" or "not coping."

### 2. **Monitoring and Communication are Key**
* Regular scans, bloodwork, or physical assessments help your care team keep close watch.
* Don’t hesitate to **ask your oncologist** about what to expect, your current response to treatment, and next steps. Sometimes the unknown causes more anxiety than the facts.

### 3. **Your Emotions Are Also a Part of Your Care**
* Oncology isn't just about treating the disease—it’s about treating the whole person, and that includes your mental health.
* Persistent anxiety, fear, or sadness is **not something to "just deal with."** It’s valid, and help is available.

---
## **Steps You Can Take to Cope**

### 🌱 **1. Talk Openly (with your team, family, or a professional)**
* Share your fear with your **oncologist or nurse navigator**—they can explain what signs to look for and help you feel more in control.
* Ask to speak to an **oncology social worker** or **psychologist**—many cancer centers offer this support for free.

### 🧠 **2. Consider Psychological Tools**
* **Cognitive Behavioral Therapy (CBT)** can help you notice and challenge the unhelpful thought loops.
* Some cancer centers offer **Fear of Recurrence Programs** tailored to young people.
* **Mindfulness** and guided meditation apps (like *Headspace*, *Calm*, or *Insight Timer*) can help reduce panic when fears spike.

### 📝 **3. Journal or Track Your Emotions**
* Try writing a short note to yourself when you feel overwhelmed: "What triggered this worry?" "Is this a thought or a fact?"
* This helps you recognize patterns and communicate them better when you talk to your care team or therapist.

### 🤝 **4. Connect with Other Young People with Cancer**
* Organizations like **Stupid Cancer**, **Elephants and Tea**, or **Teen Cancer America** offer support communities specifically for young adults with cancer.
* Hearing how others cope can make you feel less isolated and give you new strategies.

### 🧬 **5. Focus on What You *Can* Control**
* Keeping to your treatment schedule.
* Asking questions when you need answers.
* Eating as well as possible, getting some movement in, sleeping when you can.
* These small, consistent things give a sense of agency when the big picture feels chaotic.

### 💊 **6. Medication for Anxiety or Depression**
* Sometimes the fear becomes overwhelming. There’s no shame in asking about short-term or ongoing medication support.
* Talk to your oncology team—they can safely guide you based on your current treatment.

---
## You Deserve Support
You're not expected to handle this alone. Fear of progression is common, but it doesn’t have to dominate your life. Getting the right support—mental, emotional, peer-based, and medical—can make a massive difference.

If you'd like, I can:
* Help you draft questions for your doctor about this fear.
* Guide you to support organizations based in your area or online.
* Recommend tools or apps that can help you manage anxiety day-to-day.

Would you like help with any of those?

Input 2: I'm really glad you reached out—what you're going through is difficult, and it's completely valid to feel fear when facing metastatic melanoma, especially as a young person.

### **Understanding Your Fear: Why It Happens**
Fear of progression (FoP) is common in people with cancer, especially metastatic cancer. For adolescents and young adults (AYAs), this fear can be even more intense because:

* You're in a life stage focused on building a future (career, relationships, independence).
* You may feel isolated, since most people your age are not dealing with serious illness.
* You're faced with uncertainty and loss of control over your body and future.

Your fear doesn't mean you're weak or ungrateful for treatment—it means you're human.
---
## 🧠 What You Need to Know
### 1. **Metastatic Melanoma Outlook Has Improved**
* **New treatments (immunotherapy and targeted therapy)** have significantly improved outcomes in recent years.
* While metastatic melanoma is still serious, many people now live longer and better-quality lives than ever before.

### 2. **Worry Doesn't Always Match Reality**
* It's common for fear to spike even when scans are stable.
* Anxiety often comes before scans (known as "scanxiety") or during treatment side effects.
---
## 🛠 Steps You Can Take to Cope With the Fear

### **1. Talk to Your Medical Team**
* **Ask clear questions** about your current status and what signs of progression to watch for.
* Let them know you’re feeling increased fear—they can refer you to psycho-oncology or counseling resources.

### **2. Ground Yourself in Facts**
* Keep a journal of scan results and treatments to remind yourself what’s actually happening.
* Use trusted sources like:

 * [Melanoma Research Foundation](https://www.melanoma.org/)
 * [Cancer.Net (AYA-specific)](https://www.cancer.net/navigating-cancer-care/young-adults)
 * Your cancer center’s patient education resources

### **3. Manage What You *Can* Control**
* Stick to treatment, attend appointments, and ask questions.
* Prioritize sleep, movement, hydration, and nutrition (small goals help).

### **4. Emotional & Mental Health Support**
* **Talk to a counselor or therapist**, especially one trained in cancer or chronic illness.
* Cognitive Behavioral Therapy (CBT) and mindfulness-based stress reduction (MBSR) are proven to help reduce fear of progression.

### **5. Find AYA Peer Support**
* You're not alone. Connecting with others your age who are going through this can reduce fear and isolation.
* Try:

 * **Stupid Cancer** (peer groups & meetups)
 * **Elephants and Tea**
 * **Dear Jack Foundation**
 * **I’m Too Young For This! Cancer Foundation**

### **6. Practice “Both-And” Thinking**
* It’s okay to be **hopeful and scared** at the same time.
* Allow yourself to live your life as fully as possible *while* holding space for your fear.

---
## 🧘‍♂️ Coping Tools You Can Try Today

| Strategy | Description |
| ----------------------- | ----------------------------------------------------------------------------------- |
| **Box breathing** | Inhale 4 sec → Hold 4 → Exhale 4 → Hold 4. Repeat. |
| **Worry time** | Set a 10-minute timer to think about your fear. Outside that time, gently redirect. |
| **Gratitude journal** | Write down 1–2 small things each day that gave you joy or comfort. |
| **Guided meditation** | Try apps like Insight Timer, Headspace (some offer cancer-specific tracks). |
| **Creative expression** | Art, writing, music, or voice notes—whatever helps you process. |

---
## 🧡 Final Thought

Living with metastatic melanoma as a young person is not just about managing disease—it’s about learning to **live with uncertainty** and still hold on to meaning, hope, and connection.

Would you like help finding AYA cancer resources near you, or specific mindfulness tools for coping with fear?

Input 3: I'm really glad you reached out with this. It’s completely valid and understandable to feel increased fear when living with metastatic melanoma — especially when you're young. Fear of your cancer spreading (also called **fear of progression**) is something many people in your situation experience, and you are *not alone*. Here’s what you need to know and some helpful, practical steps you can take:

---

### 🧠 What You Need to Know

#### 1. **Your Emotions Are Valid**
* Fear of progression is *common*, especially among adolescents and young adults (AYA) with cancer.
* You're navigating a life-threatening illness *and* the typical challenges of young adulthood — that's a lot.

#### 2. **The Fear Won’t Always Stay This Intense**
* Fear may spike around scan times ("scanxiety"), treatment changes, or physical changes.
* Over time, many people find ways to manage their worry, even if it doesn’t disappear completely.

#### 3. **Worry Is Not the Same as Reality**
* Feeling more worried doesn’t necessarily mean your cancer *is* spreading.
* Stay grounded in your clinical information — if you're unsure or unclear, ask your team for updates.

---
### ✅ Steps You Can Take to Cope

#### 1. **Talk Openly with Your Oncologist or Nurse**
* Share that your worry has increased — they can give you personalized information and reassurance.
* Ask about your current scans, markers, and what signs they look for in disease progression.

#### 2. **Ask About Supportive Care Services**
* Psychosocial oncology teams, counselors, or therapists who specialize in cancer can help a lot.
* Many cancer centers offer free or low-cost therapy for AYA patients.

#### 3. **Build Coping Routines**
Try one or more of these strategies:

* **Mindfulness and Meditation**: Apps like *Headspace* or *Insight Timer* have cancer-specific tracks.
* **Journaling**: Write out your fears — often helps bring clarity and emotional release.
* **Scheduled Worry Time**: Give yourself 10–15 minutes daily to feel the fear. Outside of that time, gently redirect your thoughts.

#### 4. **Connect with Others**
* Join AYA cancer support groups — talking to peers can make a big difference.
* Online communities (like Stupid Cancer, Elephants and Tea, or CancerCare) can be very helpful.

#### 5. **Engage in Meaningful Activities**
* Pursue things that bring you joy, connection, or purpose — even small things.
* When your mind is busy and engaged, it can reduce spiraling worry.

---
### 🛑 When to Seek Extra Help

Reach out to a mental health professional if:
* Your fear is affecting your sleep, appetite, or ability to function.
* You feel overwhelmed daily or struggle to enjoy anything.
* You’re experiencing panic attacks, depression, or hopelessness.

You deserve support that goes beyond just treating your cancer. Treating your mental health is just as critical.
---
### You’re Not Alone

Even though this is an incredibly isolating experience at times, there is support out there — and you *can* build tools to help you live with uncertainty and fear. You’re showing strength just by acknowledging how you feel and asking for support.

If you want, I can help you write questions to ask your oncologist or help find online support groups specifically for AYA metastatic melanoma patients.

Would you like that?

Input 4: I'm really sorry you’re dealing with this — it’s completely understandable to feel fear when living with metastatic melanoma. That fear is valid. But you're not powerless in facing it. Here's what you should know and what you can do to help manage it:

---
## 🧠 What You Need to Know About Fear of Progression
1. **Fear of progression is common — and normal**
 Many adolescents and young adults (AYA) with cancer experience anxiety about recurrence or progression. It doesn’t mean you’re weak or ungrateful for treatment; it means you care deeply about your life and your future.

2. **It’s a recognized psychological challenge**
 The fear of cancer spreading (often called "Fear of Progression" or "FoP") can be intense, even when your medical team tells you things are stable. It can affect sleep, concentration, relationships, and mood.

3. **There are patterns to this fear**
 It often spikes before scans (“scanxiety”), after hearing about other patients’ stories, or when symptoms arise — even ones unrelated to cancer.

---
## 🛠️ What You Can Do to Cope

### 1. **Talk to your medical team**
* **Ask for clarity** on your current condition and what signs to watch for. Sometimes fear is worsened by uncertainty or misinformation.
* Consider asking: *“Can you help me understand what would be signs of progression, and what isn’t?”*

### 2. **Acknowledge the fear — don't suppress it**
* Try not to fight the feeling. Instead, name it. Write it down, tell someone, or say it aloud: *“I’m scared the cancer is spreading.”* Naming it reduces its grip.

### 3. **Use grounding techniques**
When fear spirals, try:

* **5-4-3-2-1 technique:** Name 5 things you see, 4 you feel, 3 you hear, 2 you smell, and 1 you taste.
* **Deep breathing:** Inhale for 4, hold for 4, exhale for 4.
* **Mindfulness apps:** Insight Timer, Headspace, or Calm (many have free cancer-focused content).

### 4. **Connect with others your age**
* Being an AYA with cancer can feel isolating.
* Consider groups like:
 * **Stupid Cancer** – AYA-specific support and meetups.
 * **Elephants and Tea** – Stories by AYAs with cancer.
 * **GRYT Health** – Digital platform for young adults with cancer.

### 5. **Speak with a mental health provider**
* Ask for a therapist or psychologist who understands oncology.
* **Cognitive Behavioral Therapy (CBT)** and **Acceptance and Commitment Therapy (ACT)** have proven helpful in managing fear of progression.

### 6. **Ask about palliative care — even now**
* Palliative care isn’t just for end-of-life. It's for improving quality of life during treatment. They can help manage physical symptoms **and** emotional distress.

---
## 📅 Practical Step You Can Take This Week
* ✅ **Make a list of questions** to ask your oncologist about your current situation.
* ✅ **Schedule a session** with a counselor or psychologist (ask your cancer center — most have one).
* ✅ **Join one support group** (online or in-person) for AYA cancer patients.
* ✅ **Practice grounding daily** — even just 5 minutes.
---
## ✍️ If It Helps to Journal
Here are a few journal prompts:
* *What am I most afraid of right now?*
* *What helps me feel safe or calm, even briefly?*
* *What do I want to say to this fear?*
---
## 🌱 Final Note

This fear can feel like it will never leave — but many patients find that it comes in waves, and that support, education, and meaningful connection make it much more manageable.

You're not alone. And you're not doing anything wrong by feeling this way.

If you'd like, I can help you draft questions for your oncologist or explore support options near you.

Input 5:

I'm really sorry you're going through this. Facing metastatic melanoma is a serious and difficult journey, and feeling more worried — especially about the cancer spreading — is completely understandable. You're not alone in this, and it's important to know that there *are* ways to cope with that fear and take steps toward feeling more supported and in control.

---
### 💡 **What You Need to Know**

1. **Your Feelings Are Valid**
 * Fear of progression (also called "scanxiety" or fear of recurrence) is **very common** in people with cancer, especially those who are young and facing a metastatic diagnosis.
 * This fear often spikes around scans, new symptoms, or changes in treatment.

2. **Understanding Metastatic Melanoma**
 * Treatments have advanced *significantly* in recent years (e.g., immunotherapies and targeted therapies), and many patients are now living much longer with better quality of life.
 * Your healthcare team can give you the most accurate picture of how your cancer is responding to treatment. Ask them directly about your current status if you're uncertain.

3. **You’re Not Alone**
 * Other young people facing similar diagnoses have the same worries. There are support systems and resources specifically for adolescents and young adults (AYA) with cancer.

---
### 🧭 Steps You Can Take to Cope With This Fear

#### 1. **Talk to Your Medical Team**
* Let them know you’ve been feeling more anxious.
* Ask specific questions:
 → *“How is my cancer responding to treatment right now?”*
 → *“Are there signs it’s spreading?”*
 → *“What symptoms should I look out for?”*
* Sometimes, understanding the facts can reduce the anxiety that comes from uncertainty.

#### 2. **Mental Health Support**
* Consider seeing a **psychologist, counselor, or psychiatrist** who specializes in cancer care (psycho-oncology).
* Cognitive Behavioral Therapy (CBT) and Acceptance and Commitment Therapy (ACT) have been shown to help people cope with fear of cancer recurrence or progression.
* If anxiety is severe, medication might be an option your provider can discuss.

#### 3. **AYA Cancer Support Programs**
* Look for AYA programs at your hospital or online (e.g., **Stupid Cancer**, **Elephants and Tea**, **Teen Cancer America**).
* Talking with people your age who “get it” can be incredibly helpful and validating.

#### 4. **Mindfulness and Stress-Reduction Techniques**
* Practices like **guided imagery, breathing exercises, meditation, yoga**, or even journaling can reduce stress and help bring your focus back to the present.
* Try apps like **Calm**, **Headspace**, or **Insight Timer** — many have cancer-specific meditations.

#### 5. **Stay Engaged in Life**
* When fear takes up space, try to also make space for joy and meaning — hobbies, music, connection, nature, creativity, humor — whatever gives you a sense of **you-ness**.

---
### 🛠️ Specific Coping Tools
* **Fear Inventory**: Write down what you're afraid of, what triggers those fears, and what helps ease them.
* **Grounding Techniques**: When you're overwhelmed, name 5 things you can see, 4 you can touch, 3 you can hear, 2 you can smell, and 1 you can taste.
* **Create a “Worry Window”**: Give yourself 15 minutes a day to worry as much as you want — then try to refocus afterward.
* **Ask for Updates Regularly**: Schedule brief updates from your care team so you’re not left guessing.
* **Create a “Control List”**: What *can* you control today? That can include hydration, meds, how you talk to yourself, and who you connect with.
---
### 🌱 Final Thought

You're facing something incredibly tough — and you're still here, still asking good questions, still trying to live your life. That says a lot about your strength, even if you don’t always feel strong. Coping with the fear of cancer spreading isn’t about getting rid of the fear entirely — it’s about learning how to live *with* it in a way that still allows you to find meaning, connection, and moments of peace.

If you'd like, I can help you find specific support groups or mental health services in your area. You don’t have to go through this alone.

**Question 3: I am an adolescent/young adult patient with metastatic melanoma and am currently receiving treatment. How will my treatment affect long-term hormone changes?**

Input 1:

I'm really glad you’re asking this—understanding the long-term effects of melanoma treatment, especially on hormones, is crucial for adolescents and young adults (AYA). Here's an overview tailored to your situation:

### 🔬 **How Metastatic Melanoma Treatment May Affect Hormone Levels Long-Term**

#### **1. Type of Treatment Matters**

The impact on your hormones largely depends on the treatment you're receiving. Common treatments for metastatic melanoma include:

* **Immunotherapy (e.g., checkpoint inhibitors like nivolumab, pembrolizumab)**

* **Targeted therapy (e.g., BRAF and MEK inhibitors)**

* **Radiation**

* **Chemotherapy (less commonly used now for melanoma)**

Each of these can have different effects on the endocrine (hormone) system.

### 🧠 **Most Affected Hormone Systems**

**Checkpoint inhibitors** (especially **anti-CTLA-4** and **anti-PD-1** therapies) can cause **immune-related adverse events (irAEs)** that may impact hormone-producing glands, including:

#### 🔹 Thyroid Gland

* **Hypothyroidism** or **hyperthyroidism** can occur.

* Sometimes this damage is **permanent**, requiring lifelong thyroid hormone replacement (like levothyroxine).

#### 🔹 Pituitary Gland (Hypophysitis)

* This is inflammation of the pituitary, which can reduce production of key hormones.

* Can lead to deficiencies in:

* **Cortisol** (via ACTH)

* **Thyroid-stimulating hormone (TSH)**

* **Sex hormones** (LH/FSH)

* May result in **adrenal insufficiency** or **hypogonadism** (affecting puberty, menstrual cycles, libido, fertility).

#### 🔹 Adrenal Glands

* May be affected directly or via pituitary damage.

* Could cause **fatigue, low blood pressure, and need for steroid replacement**.

#### 🔹 Gonadal Function (Ovaries/Testes)

* Less commonly affected directly by immunotherapy, but:

* **Targeted therapy or radiation** may impact **fertility or hormone production**.

* For those who receive **brain radiation**, the hypothalamic-pituitary axis might be disrupted.

### 🧬 **Long-Term Consequences in AYA Patients**

Because you’re still in a phase of physical and sexual development, hormone changes can significantly affect:

* **Puberty progression or delay**

* **Menstrual regularity or early menopause**

* **Testosterone levels (libido, muscle mass, mood)**

* **Bone density**

* **Fertility**

### ✅ **What You Can Do**

1. **Regular Endocrine Monitoring**

* Request regular hormone bloodwork:

* TSH, free T4

* ACTH and cortisol

* LH/FSH

* Estradiol/testosterone

* Prolactin

* Electrolytes (esp. sodium)

* Ask your oncologist to refer you to an **endocrinologist**.

2. **Discuss Fertility Preservation**

* If not already done, discuss **egg/sperm banking** before future treatments that could impact fertility.

3. **Stay Alert to Symptoms**

* Report fatigue, weight changes, mood shifts, missed periods, or libido changes promptly.

4. **Lifelong Follow-Up**

* Some hormone issues (like adrenal insufficiency) require **lifelong treatment and monitoring**.

Would you like to tell me which specific treatment you're receiving? That way I can tailor this even more accurately.

Input 2:

I'm really glad you're reaching out with this question — it’s an important one. Since you're an adolescent or young adult (AYA) with **metastatic melanoma**, your treatment plan may include **immunotherapy, targeted therapy, chemotherapy**, or **radiation**, and each of these can have **different long-term effects on hormones**. Here's a breakdown of what might happen, based on current knowledge:

---

### 💊 **1. Immunotherapy (e.g., checkpoint inhibitors like nivolumab or pembrolizumab)**

These drugs stimulate your immune system to attack the cancer, but they can sometimes cause the immune system to attack normal organs — including hormone-producing glands.

**Possible long-term hormone effects:**

* **Hypothyroidism or hyperthyroidism** (thyroid gland affected)

* **Adrenal insufficiency** (adrenal glands affected)

* **Hypophysitis** (inflammation of the pituitary gland → can lead to decreased production of multiple hormones like cortisol, sex hormones, and thyroid hormones)

* **Diabetes** (if the pancreas is affected)

👉 These endocrine side effects may be **permanent** and require **lifelong hormone replacement**.

---

### 🎯 **2. Targeted therapy (e.g., BRAF or MEK inhibitors for BRAF-mutant melanoma)**

Used in patients whose tumors have specific mutations (like BRAF V600E). These therapies are generally less likely to directly affect hormone levels, but:

**Possible effects:**

* May **interact with hormone metabolism** or medications

* Indirect effects if used long-term or combined with other treatments

---

### ☢️ **3. Radiation therapy**

If you receive **radiation to the brain** (e.g., for brain metastases), this can damage the **pituitary gland**, which controls:

* Growth hormone

* Sex hormones (estrogen, testosterone)

* Thyroid hormone

* Cortisol

This damage can lead to **hypopituitarism**, which often requires **lifelong hormone replacement**.

---

### 💉 **4. Chemotherapy** (less common for melanoma, but sometimes used)

Chemotherapy in adolescents and young adults can:

* Affect **gonadal function** (ovaries or testes)

* Lead to **premature menopause** or **low testosterone**

* Impact **fertility**

This is more relevant if you're receiving **cytotoxic agents** or undergoing **stem cell transplant**, though these are not typically first-line in melanoma.

---

### 👩‍⚕️ What You Can Do:

* Ask your oncology team for **baseline and regular hormone testing** (thyroid function, cortisol, sex hormones)

* If you notice symptoms like **fatigue, weight changes, menstrual changes, mood swings, or sexual dysfunction**, report them promptly

* Consider a referral to an **endocrinologist** experienced in cancer survivorship or AYA care

* If fertility is a concern, **ask about preservation options** if still relevant

---

### Summary Table

| **Treatment** | **Potential Long-Term Hormone Effects** |

| ----------------- | ---------------------------------------------------------------- |

| Immunotherapy | Hypothyroidism, adrenal insufficiency, pituitary dysfunction |

| Targeted therapy | Rare direct effects, possible indirect hormone changes |

| Radiation (brain) | Pituitary damage → multi-hormonal deficiencies |

| Chemotherapy | Gonadal failure → early menopause, infertility, low testosterone |

---

Would you like a checklist to take to your oncologist or endocrinologist to guide this conversation?

Input 3

I'm really glad you're reaching out with this important question. While I can’t give medical advice, I can share some information to help guide your discussion with your healthcare team.

### Long-term Hormonal Changes and Metastatic Melanoma Treatment

The impact of your treatment on hormones depends on **what kind of therapy you're receiving**. Here’s how the most common types of treatment for metastatic melanoma can affect your hormones:

---

### 1. **Immunotherapy (e.g., anti-PD-1 like nivolumab, pembrolizumab)**

This is commonly used in metastatic melanoma.

#### Hormonal Side Effects:

* **Immune-related endocrinopathies** can occur, including:

- Hypophysitis** (inflammation of the pituitary gland): can lead to *low levels of multiple hormones* (cortisol, thyroid hormone, sex hormones).

-Thyroid dysfunction**: can cause *hypothyroidism* or *hyperthyroidism*.

-Adrenal insufficiency**.

-Type 1 diabetes** (less common, but possible).

These may be **permanent** in some cases and require lifelong hormone replacement (e.g., thyroid hormone, corticosteroids, sex hormones).

---

### 2. **Targeted Therapy (e.g., BRAF and MEK inhibitors like dabrafenib + trametinib)**

Used for melanoma with **BRAF V600 mutations**.

#### Hormonal Side Effects:

* These generally do **not** directly affect hormones.

* However, rare side effects could include **fever**, fatigue, and possible effects on the hypothalamic-pituitary axis indirectly (though less common than with immunotherapy).

---

### 3. **Chemotherapy (less common now in melanoma)**

* Can affect hormones, especially **gonadal function** (sex hormones).

* In adolescents/young adults, this may mean:

-Early menopause** or menstrual irregularities in females.

-Reduced sperm production** or testosterone levels in males.

Ask your care team if **fertility preservation** was or should be discussed if this applies.

---

### 4. **Radiation Therapy**

* If the **brain** or **pituitary gland** is irradiated (for brain metastases), this can cause:

* **Hypopituitarism**, affecting growth hormone, thyroid hormone, adrenal hormones, and sex hormones.

* The damage can be **permanent** and may require hormone replacement.

---

### What You Can Do:

* **Endocrine monitoring**: Your oncology team should check your hormone levels regularly (TSH, cortisol, sex hormones, etc.).

* If you're having symptoms like **fatigue, weight changes, mood changes, or changes in sexual function or menstruation**, tell your doctor—these could be hormone-related.

* Ask about a **referral to an endocrinologist**, especially if you’ve had endocrine side effects or brain radiation.

---

### Summary

As a young adult, preserving your hormonal health is essential—not just for current wellbeing but also for long-term issues like:

* Fertility

* Bone health

* Metabolism

* Mood and cognition

Even if your cancer treatment is working well, keeping an eye on your endocrine function is important.

Would you like help preparing specific questions to ask your doctor or oncologist about this?

Input 4

I'm really glad you asked this—it's an important and often overlooked part of cancer treatment, especially for adolescents and young adults (AYA). Metastatic melanoma treatments can affect your hormones in different ways depending on the **type of treatment you're receiving**, your **age**, your **sex assigned at birth**, and your **baseline hormone levels**.

Here’s a breakdown of how **metastatic melanoma treatments** might affect long-term hormone levels:

---

### 🧪 1. **Immunotherapy (e.g., checkpoint inhibitors like nivolumab, pembrolizumab, or ipilimumab)**

These are commonly used for metastatic melanoma.

#### 🔥 Risk: Autoimmune endocrine side effects

* These treatments can cause **immune-related adverse events (irAEs)** where the immune system attacks hormone-producing glands.

* **Most commonly affected glands**:

* **Thyroid** → Hypothyroidism or hyperthyroidism

* **Pituitary gland (hypophysitis)** → Can lead to deficiencies in:

* Cortisol (adrenal insufficiency)

* Gonadotropins (affecting sex hormones like estrogen or testosterone)

* Thyroid-stimulating hormone (TSH)

* **Adrenal glands**

#### 📉 Long-Term Impact:

* May require **lifelong hormone replacement therapy** (e.g., thyroid hormone, cortisol, testosterone/estrogen)

* Effects may **not reverse** even after stopping treatment

---

### 💊 2. **Targeted Therapy (e.g., BRAF/MEK inhibitors like dabrafenib + trametinib)**

Used if your tumor has a **BRAF mutation**.

#### 🔥 Risk: Less common hormone impact

* These therapies generally **do not directly affect hormone levels** long-term.

* Rare cases of:

* **Fever and fatigue** that may indirectly affect the endocrine system

* Possible **impact on fertility**, though less studied

---

### 🧠 3. **Radiation Therapy (especially if treating brain metastases)**

Radiation to the brain can damage the **hypothalamic-pituitary axis**.

#### 📉 Long-Term Impact:

* Hormone production (like growth hormone, sex hormones, ACTH, and TSH) may decline

* May cause **central adrenal insufficiency** or **gonadal failure**

---

### 🎯 4. **Surgical Treatment (e.g., adrenalectomy)**

If surgery involves hormone-producing glands (e.g., adrenal glands), this can directly cause **hormonal deficiencies**.

---

### ⚠️ What You Might Notice or Experience:

* **Fatigue**

* **Cold intolerance, weight gain** → Suggests hypothyroidism

* **Low libido, irregular periods, hot flashes** → Suggests low sex hormones

* **Mood changes, depression**

* **Increased thirst/urination** → Possible diabetes insipidus if pituitary involved

---

### 🧬 Special Considerations for AYA Patients:

* **Fertility preservation** is a key concern:

* Chemotherapy (less common in melanoma) and some hormone changes can affect sperm or egg production

* Ask your oncologist about sperm banking, egg freezing, or ovarian suppression (if applicable)

Pubertal development** can be affected in younger adolescents

---

### ✅ What You Should Do:

1. **Ask for baseline and follow-up hormone panels**, especially:

* TSH, Free T4

* Cortisol

* ACTH

* LH, FSH

* Estradiol or testosterone

2. **See an endocrinologist** if you develop symptoms or lab abnormalities

3. **Talk to your team about fertility and sexual health early** in your treatment journey

4. **Keep records** of any hormone replacements you start

---

If you tell me what treatment(s) you're currently on (e.g., specific immunotherapy or targeted drugs), I can give you more personalized insight.

Would you like help drafting questions to ask your oncologist or endocrinologist?

Input 5

Thanks for sharing that. Metastatic melanoma treatments can vary, but many common ones—like immunotherapy, targeted therapy, or sometimes radiation—might have different impacts on your hormones in the long run. Here’s a general overview of what you might expect:

### Immunotherapy (e.g., checkpoint inhibitors like nivolumab, pembrolizumab)

* These treatments help your immune system attack cancer cells.

* Sometimes, they can cause **immune-related side effects** where your immune system attacks healthy tissues, including hormone-producing glands like the thyroid, pituitary, or adrenal glands.

* This can lead to **long-term hormone issues** such as hypothyroidism (low thyroid hormone), adrenal insufficiency, or hypophysitis (pituitary inflammation), which may require lifelong hormone replacement.

### Targeted Therapy (e.g., BRAF or MEK inhibitors)

* These drugs target specific mutations in melanoma cells.

* They generally have fewer direct effects on hormone glands, but side effects vary by drug.

* Long-term hormone effects are less common but still possible depending on side effects or if other organs are involved.

### Radiation Therapy

* If you receive radiation to areas near hormone glands (like the brain/pituitary or adrenal glands), this can impact hormone production.

* Radiation to the brain may affect pituitary function, leading to hormone imbalances that might require replacement therapy.

### Other Considerations

* Stress from cancer and treatment can also temporarily disrupt hormone balance.

* Some hormone effects may develop months or years after treatment, so regular monitoring is important.

* Your healthcare team will typically check your hormone levels during and after treatment and help manage any issues.

---

**Bottom line:**

Long-term hormone changes are possible, especially with immunotherapy or radiation involving hormone glands. These changes are often manageable with hormone replacement and monitoring. It’s important to keep your doctors informed of any symptoms like fatigue, weight changes, mood shifts, or temperature intolerance, which might signal hormone imbalances.

If you want, I can help you understand what symptoms to watch for or how hormone testing works. Would you like that?

**Question 4: I am an adolescent/young adult patient with metastatic melanoma and am currently receiving treatment. I want more information about how my genetics may or may not have impacted my diagnosis and treatment.**

Input 1

I'm really glad you’re looking into how your genetics might influence your metastatic melanoma. Understanding this can give you more insight into why certain treatments were chosen and what options might be available going forward. Here's a breakdown tailored to adolescent and young adult (AYA) patients:

---

### 🧬 **How Genetics May Have Contributed to Your Diagnosis**

#### 1. **Inherited (Germline) Mutations**

Some people are born with mutations in genes that increase their risk of melanoma. These are inherited from one or both parents and are present in every cell of the body.

* **CDKN2A**: The most common inherited mutation linked to melanoma. It increases the risk of both melanoma and pancreatic cancer.

* **CDK4**, **BAP1**, **MITF**, and **POT1**: Other, less common genes associated with inherited melanoma risk.

* Family history of melanoma, especially multiple family members or multiple melanomas in one person, raises suspicion for a hereditary cause.

🧪 If you haven't already, a **genetic counseling session** and possible **germline genetic testing** could help identify inherited risk and may impact your care and your family’s.

---

### 🧬 **Tumor (Somatic) Genetics and Their Impact on Treatment**

These are changes that happen in the melanoma cells themselves and are **not inherited**—they develop during your life. These mutations are a big part of why melanoma forms and how it behaves, especially in younger patients.

#### 1. **BRAF Mutations**

* Found in about **40-60%** of melanomas.

* Especially common in **younger** patients.

* **BRAF V600E** is the most common type.

* **Targeted therapies** like **dabrafenib + trametinib** or **vemurafenib + cobimetinib** are used to block this pathway.

#### 2. **NRAS Mutations**

* Found in \~15-20% of melanomas.

* These tumors don't respond to BRAF inhibitors.

* Clinical trials or MEK inhibitors may be options.

#### 3. **NF1 and KIT Mutations**

* Less common but can occur in some melanoma subtypes.

* KIT mutations are more common in **acral** (palms, soles) or **mucosal** (inside body surfaces) melanomas.

#### 4. **Tumor Mutational Burden (TMB) and PD-L1**

* These tests help predict whether **immunotherapy** will work well.

* High TMB = more mutations = possibly better response to immune checkpoint inhibitors (like nivolumab or pembrolizumab).

---

### 🧪 **Genetic Testing in Practice**

You might have had or could still have:

* **Germline genetic testing** – to assess inherited risk.

* **Tumor genomic profiling** – to guide treatment (like targeted therapy or immunotherapy).

You can ask your care team:

* “Have I had both germline and tumor genetic testing?”

* “What were the results of my BRAF/NRAS/KIT testing?”

* “Would any genetic results affect my siblings or children?”

---

### 💡 Why Genetics Matter for AYAs Specifically

* AYA patients often have **different mutation patterns** than older adults.

* You're more likely to have **BRAF-mutant tumors**, which respond to targeted therapy.

* Younger immune systems may also respond **differently** to immunotherapy.

* Understanding your genetics can help with **clinical trial eligibility**, which is especially important in advanced melanoma.

---

### ✅ What You Can Do Next

1. **Request a copy of your genetic and molecular testing results** (if available).

2. **Ask for a referral to a genetic counselor**, especially if there's any family history of cancer.

3. **Talk to your oncologist** about whether your treatment is based on a known genetic mutation.

4. **Explore clinical trials** if standard treatments are not effective—some are mutation-specific.

---

Would you like help interpreting any specific genetic test results you’ve already received? Or would you like help preparing questions for your next appointment with your oncology/genetics team?

Input 2

I'm really glad you're asking this — understanding the role of genetics in metastatic melanoma can give you more clarity about your diagnosis and treatment. Here's an overview tailored for an adolescent or young adult (AYA) patient like you:

---

## 🔬 **Genetics and Melanoma: What You Should Know**

### 1. **Inherited (Germline) Genetics: Did You Inherit a Risk?**

Some people are born with gene mutations that increase their risk of developing melanoma. These are inherited from one or both parents and are present in all the cells of your body.

* **Common inherited gene mutations linked to melanoma:**

* **CDKN2A**: The most commonly mutated gene in familial melanoma.

* **CDK4, BAP1, TERT, MITF**: Less common, but still linked to hereditary melanoma risk.

* **Signs you might have an inherited predisposition:**

* Multiple family members with melanoma or other cancers.

* You had melanoma at a young age.

* You have many atypical moles or a history of other skin cancers.

> 💡 If you haven’t already, ask your care team about **genetic counseling and testing** for hereditary cancer risk. It can help guide not only your care, but also screening recommendations for your family.

---

### 2. **Somatic (Tumor) Mutations: What’s Driving Your Cancer?**

These mutations happen in the melanoma cells themselves and are not inherited. They influence **how your melanoma behaves** and how it responds to **targeted therapy**.

* **Most important tumor mutations in melanoma:**

* **BRAF mutations** (especially **BRAF V600E**): Found in about 40–50% of melanomas, especially in younger patients.

* Targeted therapies: **BRAF inhibitors** (like dabrafenib) + **MEK inhibitors** (like trametinib).

* **NRAS mutations**: Seen in \~20% of melanomas; currently no direct targeted therapies, but may affect immunotherapy response.

* **NF1, c-KIT, GNAQ/GNA11**: Other mutations, more common in rare melanoma subtypes.

> 🧪 If you have metastatic melanoma, your doctors likely performed **molecular testing** on your tumor to look for these mutations — this helps guide treatment choices.

---

### 3. **Immunotherapy Response and Genetics**

Even if your tumor doesn’t have a targetable mutation, many patients benefit from **immunotherapy** (like checkpoint inhibitors).

* Some genetic markers (like **high tumor mutational burden**, **PD-L1 expression**, or certain **HLA types**) may influence how well immunotherapy works — though these aren’t always used in clinical decision-making.

---

### 4. **Why Genetics Matters in Young People with Melanoma**

As an AYA patient, your case may be different from older adults:

* **You’re more likely to have certain mutations (like BRAF).**

* You may have **long-term survivorship** to consider — making personalized treatment and monitoring even more important.

* If an inherited mutation is found, it could affect your **future family planning decisions** (e.g., preimplantation genetic testing, if desired).

---

## ✅ What You Can Do Next

1. **Ask your oncologist:**

* “Has my tumor been tested for BRAF, NRAS, or other mutations?”

* “Should I see a genetic counselor to check for inherited cancer risk?”

2. **If you’re interested in research or clinical trials**, knowing your tumor’s genetic profile could open more doors.

3. **Talk with your family** about any history of cancer — it can help with your genetic risk assessment.

---

Would you like help preparing questions to ask your doctor, or more details on specific mutations or treatments?

Input 3

I'm really glad you're asking this—understanding how your genetics may influence your melanoma can help you take an active role in your treatment and health decisions. Here's a breakdown of how genetics can impact both **melanoma risk** and **treatment options**, especially for someone your age.

---

## 🧬 Genetics and Melanoma: What You Should Know

### 1. **Inherited (Germline) Genetics**

These are the genes you were born with, which can increase your **risk** for developing melanoma.

#### Key Genetic Mutations:

* **CDKN2A**: The most common high-risk gene associated with familial melanoma. Mutations here increase lifetime risk and can also be linked to pancreatic cancer.

* **CDK4, BAP1, TERT, POT1**: Less common, but also associated with familial melanoma risk.

* **MC1R**: More common variant, linked to red hair, fair skin, and increased melanoma risk, especially when combined with UV exposure.

👉 **If you have a strong family history** of melanoma or other cancers, you might benefit from genetic counseling or germline testing.

---

### 2. **Tumor (Somatic) Genetics**

These are mutations that happen in the cells of the melanoma tumor itself. These mutations **do not get passed down**, but they directly impact how your disease behaves and responds to treatment.

#### Key Somatic Mutations in Melanoma:

* **BRAF mutations** (especially V600E and V600K): Found in \~40–60% of cutaneous melanomas, especially in younger patients.

* If you have a **BRAF mutation**, you may be treated with **targeted therapies** like **BRAF and MEK inhibitors** (e.g., dabrafenib + trametinib).

* **NRAS mutations**: Occur in about 15–20% of cases; not currently directly targetable, but may influence immunotherapy response.

* **KIT mutations**: More common in mucosal or acral melanomas. These can be treated with certain targeted therapies (like imatinib), although results vary.

* **TP53, TERT promoter, NF1**: Common in some melanoma subtypes and may be involved in resistance to treatment.

Your oncologist may have already done **molecular profiling** (like next-gen sequencing or a comprehensive tumor panel) to determine if any of these mutations are present.

---

### 3. **Impact of Genetics on Treatment**

Your genetics can influence:

#### ✅ **Targeted Therapy Eligibility**

* **BRAF mutation?** → Eligible for BRAF/MEK inhibitors.

* **KIT mutation?** → May benefit from KIT inhibitors (though less commonly used).

#### ✅ **Immunotherapy Response**

* While not directly based on genetics, **tumor mutational burden (TMB)** and other molecular markers may affect how well you respond to immunotherapy (like checkpoint inhibitors: nivolumab, pembrolizumab).

* Certain genetic profiles might be linked with **immune "cold" tumors**—less likely to respond to immunotherapy.

#### ✅ **Clinical Trials**

* Young patients with specific mutations may qualify for targeted or immunotherapy-based trials.

* Knowing your genetic profile can open access to cutting-edge treatments.

---

### 4. **Special Considerations for Adolescent and Young Adult (AYA) Patients**

* AYA patients with melanoma may have **different mutation patterns** than older adults.

* More likely to have BRAF mutations.

* Often present with **thicker tumors or nodal involvement**.

* May benefit from **AYA-specific oncology programs**, which integrate survivorship, mental health, and genetic counseling.

---

## ✅ What You Can Do Next

1. **Ask your care team:**

* “Has my tumor been genetically profiled?”

* “Was I tested for a BRAF mutation or other mutations?”

* “Should I consider germline genetic testing based on my family history?”

* “Are there any clinical trials I’m eligible for based on my mutation profile?”

2. **Consider genetic counseling** if you:

* Were diagnosed at a young age

* Have a personal or family history of melanoma or other cancers

3. **Stay updated on precision oncology tools.**

* Tumor sequencing and biomarkers are rapidly evolving.

* Liquid biopsies and circulating tumor DNA (ctDNA) are also being researched for monitoring treatment response.

---

Would you like help drafting questions to ask your oncologist or understanding your genetic test results if you have them? I can also explain specific mutations if you’ve been told you have one.

Input 4

I'm really glad you’re taking an active role in understanding your condition — that’s important. Here's an overview tailored specifically to **adolescent and young adult (AYA)** patients with **metastatic melanoma**, focusing on how **genetics** may have impacted your **diagnosis and treatment**.

---

### 🧬 1. **How Genetics Can Influence Melanoma in AYA Patients**

#### A. **Inherited (Germline) Mutations**

These are mutations you're born with, present in every cell of your body.

* **CDKN2A**: This is the most well-known gene linked to **familial melanoma**. Mutations here increase lifetime melanoma risk.

* **BAP1**: Associated with **uveal melanoma** and some cutaneous melanomas.

* **MITF, PTEN, TP53**: Less commonly, these may also play a role.

If you have a strong **family history** of melanoma or other cancers, you might benefit from **genetic counseling** to test for these mutations.

#### B. **Somatic (Tumor-Specific) Mutations**

These are changes that happen in the melanoma cells but not in the rest of your body. These mutations can **drive tumor growth** and are crucial in deciding treatment.

* **BRAF mutations (especially V600E)** — Found in \~40–60% of melanomas in younger patients.

* **NRAS** and **NF1** — Other common drivers.

* **KIT** — More frequent in acral (palms, soles), mucosal, or sun-protected areas.

* **TP53, TERT, PTEN** — Also sometimes seen and may impact prognosis.

---

### 💉 2. **How Genetics Impacts Treatment Choices**

Genetic testing of the tumor is **standard** for metastatic melanoma and directly influences treatment:

#### A. **BRAF-Mutant Melanoma**

If your tumor has a **BRAF V600E or V600K mutation**, you might be receiving:

* **Targeted therapy**: A combination of **BRAF inhibitors** (e.g., dabrafenib, vemurafenib) + **MEK inhibitors** (e.g., trametinib).

* This combo works fast but may develop resistance over time.

#### B. **BRAF Wild-Type (No BRAF Mutation)**

You may be receiving:

* **Immunotherapy**: Checkpoint inhibitors like **nivolumab**, **pembrolizumab**, or **ipilimumab**.

* These help your immune system recognize and fight the cancer, and are used in both BRAF-mutant and BRAF wild-type melanoma.

#### C. **Other Targets (Less Common)**

* If your melanoma has **KIT** mutations or **NRAS**, there may be clinical trials or targeted options.

* **Tumor mutational burden (TMB)** and **PD-L1 expression** may also influence response to immunotherapy.

---

### 🧪 3. **Should You Consider Genetic Counseling or Testing?**

As a young person with metastatic melanoma, especially if you:

* Have a **family history** of melanoma or other cancers,

* Had **multiple primary melanomas**,

* Have other unusual cancer findings,

Then yes — a **germline genetic test** could be appropriate. This might help you:

* Understand your **cancer risk** (for future planning),

* Inform screening for **relatives**,

* Contribute to research or eligibility for certain **clinical trials**.

---

### 🧭 4. **Next Steps You Can Take**

* **Ask your oncologist** if your tumor has been tested for mutations (e.g., BRAF, NRAS, KIT).

* Request a referral to a **genetic counselor** — especially if you have a strong personal or family cancer history.

* Consider enrolling in an **AYA cancer program** or research study — they often include genetic research and offer personalized support.

---

### 📚 Resources

* **Genetic Information**: [Facing Our Risk (FORCE)](https://www.facingourrisk.org)

* **AYA Support**: [Stupid Cancer](https://www.stupidcancer.org)

* **Clinical Trials**: [ClinicalTrials.gov](https://clinicaltrials.gov)

* **Genetic Counseling**: [NSGC (National Society of Genetic Counselors)](https://www.nsgc.org)

---

Would you like help coming up with **questions to ask your oncologist or genetic counselor**? Or help locating **clinical trials** based on your mutation status?

Input 5

It’s very good that you’re asking this question—understanding the role of genetics in metastatic melanoma can help guide your treatment decisions and empower you in managing your care. Here’s a breakdown of how genetics might relate to your **diagnosis, prognosis, and treatment options**:

---

### 🔬 **1. Genetic Factors and the Risk of Melanoma**

There are two main types of genetics involved:

#### **A. Inherited (Germline) Genetics**

These are the genes you were born with.

* **CDKN2A**: This is the most common gene associated with hereditary melanoma. Mutations here increase melanoma risk significantly, especially in families with multiple affected individuals.

* **Other genes**: BAP1, PTEN, TP53, and MITF are also linked with increased risk in certain cases.

* **Familial Melanoma**: If you have a family history of melanoma or other cancers, you might benefit from **genetic counseling** and testing.

#### **B. Acquired (Somatic) Mutations**

These are mutations that happen over time in skin cells due to UV damage or other factors. They’re not inherited but are crucial in treatment decisions.

---

### 🧬 **2. Somatic Mutations and How They Impact Treatment**

In metastatic melanoma, treatment is often guided by the **mutations found in the tumor itself**. Some of the most important include:

#### **BRAF mutations**

* Found in about **40–60%** of cutaneous melanomas (especially in younger patients).

* **BRAF V600E** is the most common mutation.

* **Treatment**: If you have a BRAF mutation, targeted therapy with **BRAF inhibitors** (e.g., vemurafenib, dabrafenib) and **MEK inhibitors** (e.g., trametinib) can be very effective.

#### **NRAS mutations**

* Found in \~15–20% of melanomas.

* Associated with more aggressive disease.

* No direct targeted therapy yet, but may respond to immunotherapy.

#### **KIT mutations**

* More common in mucosal and acral melanomas (palms, soles, nail beds).

* Some patients respond to **KIT inhibitors** (e.g., imatinib).

#### **NF1 and others**

* NF1-mutated melanomas may have different responses to treatment and often behave differently.

---

### 💊 **3. Immunotherapy and Genetics**

Your tumor’s genetic profile can also influence how well you respond to **immunotherapy** (e.g., checkpoint inhibitors like **nivolumab** or **pembrolizumab**):

* **Tumor Mutational Burden (TMB)**: A high number of mutations can mean more abnormal proteins on the cancer cells, which helps the immune system recognize and attack them.

* **HLA genes**: Your body's immune response is influenced by your HLA genotype, which can affect how well immunotherapy works.

---

### 🧪 **4. Genetic Testing – What to Ask About**

If it hasn’t already been done, ask your oncologist about:

* **Tumor sequencing (NGS – Next-Generation Sequencing)** to look for actionable mutations.

* **Germline genetic testing** if there is a family history or early age of diagnosis.

* **PD-L1 expression** and **TMB** if you're exploring immunotherapy options.

---

### 🧠 **5. Why This Matters for Young Adults**

* Younger melanoma patients are **more likely to have BRAF mutations**, making **targeted therapy** a highly relevant option.

* Genetic counseling may be more important in younger patients due to the possibility of **hereditary cancer syndromes**.

* Being proactive in understanding your genetic profile can help tailor your treatment and long-term monitoring plans.

---

### ✅ Summary of Action Steps:

1. **Ask your oncologist**:

* Has my tumor been tested for **BRAF, NRAS, KIT**, and other mutations?

* Should I consider **germline genetic testing**?

* What are my options for **targeted therapy vs. immunotherapy** based on my mutation profile?

2. **Consider genetic counseling**, especially if:

* You have a strong family history of cancer

* You were diagnosed at a young age (under 30)

3. **Stay informed** about clinical trials, as some are targeting rare or less common mutations.

---

Would you like help drafting specific questions to bring to your care team or understanding a genetic test report you’ve received?

**Question 5: I am an adolescent/young adult patient with metastatic melanoma and am currently receiving treatment. I want more information about the long-term side effects of treatment.**

**Input 1:**

"I’m really sorry to hear that you’re dealing with metastatic melanoma—what you're going through is incredibly challenging. While I can’t provide medical advice, I can share general, evidence-based information about long-term side effects associated with treatments like immunotherapy or targeted therapies. Always check in with your oncologist or care team for insight tailored to your individual treatment and health profile.

---

## Long-term Side Effects by Treatment Type

### 1. **Immunotherapy** (e.g., PD-1 inhibitors like pembrolizumab, nivolumab; CTLA-4 inhibitors like ipilimumab)

* **Endocrine Disorders:**

Some patients develop lasting effects like hypothyroidism, hyperthyroidism, or adrenal insufficiency. These can persist long after treatment ends and require lifelong hormone replacement.

* **Joint/Muscle Issues:**

Chronic arthritis or inflammatory joint pain has been reported, which may require ongoing management with medications or physical therapy.

* **Skin and Hair Changes:**

Persistent rashes or vitiligo (patchy loss of skin pigment) can remain long term.

* **Other Autoimmune Reactions:**

Rare but possible longer-term effects include colitis (inflammation of the colon), hepatitis, or pneumonitis, which might require extended monitoring even after treatment.

### 2. **Targeted Therapy** (e.g., BRAF and MEK inhibitors)

* **Cardiovascular Effects:**

Long-term use has been associated with the possibility of heart rhythm changes or heart muscle weakening, so periodic cardiac monitoring is often recommended.

* **Skin Toxicities:**

These can include secondary skin cancers, persistent rash, or skin sensitivity changes.

* **Eye Issues:**

Although less common, long-term eye effects such as retinal problems may arise, so regular eye check-ups may be advised.

* **Liver and Blood Effects:**

Some patients experience long-standing liver function abnormalities or blood cell changes (like anemia) that require monitoring.

--

## Next Steps and Support

* **Talk with your care team.** Ask whether your treatment might cause long-term effects and if there are screening protocols in place (e.g., regular bloodwork, hormone panels, heart o eye evaluations).

* **Consider rehabilitation support.** Referrals to endocrinologists, rheumatologists, dermatologists, cardiologists, or physical therapists may help if persistent symptoms arise.

* **Support networks and resources** such as foundations or survivorship programs often provide valuable guidance—these can be especially helpful in navigating life after treatment.

---

### Final Thoughts

While it's true that immunotherapy and targeted therapies have revolutionized melanoma treatment, they bring unique long-term considerations depending on your specific regimen. Since you’re in the adolescent/young adult stage, long-term wellness—physical, emotional, and functional—becomes even more important. Partnering proactively with your healthcare team to monitor, manage, and address any late effects can make a real difference.

If you'd like more specific information (e.g., side effects for a particular medication), just let me know—I’m here to help!

"

Input 2

"I’m really sorry to hear about everything you’re going through, and I appreciate how important it is to stay well-informed about what you might face in the long term.

Because treatments for metastatic melanoma—such as **immune checkpoint inhibitors** (e.g., nivolumab, pembrolizumab, ipilimumab) and **targeted therapies** (e.g., BRAF/MEK inhibitors like dabrafenib, trametinib)—can vary widely in their effects, I’ll share general insights. Also, if you’d like, I can look into the specific drug(s) you’re receiving for more tailored information.

---

## Immune Checkpoint Inhibitors (ICIs)

These treatments boost your immune system to fight cancer, but their effects can sometimes last long after therapy ends.

### Common long-term side effects:

* **Endocrine issues**: Thyroiditis or adrenal gland inflammation can lead to lasting hormone imbalances requiring ongoing replacement therapy.

* **Digestive tract**: Persistent colitis may cause chronic diarrhea or abdominal pain.

* **Skin**: Long-term rashes or pigment changes.

* **Muscle or nerve-related issues**: Autoimmune-related neuropathy or myositis can sometimes linger.

* **Rare but serious**: Conditions like type 1 diabetes or myocarditis may develop and require lifelong management.

---

## Targeted Therapies (e.g., BRAF and MEK inhibitors)

Used when your melanoma has a specific genetic mutation (BRAF V600), these therapies can have effects over the longer term:

* **Skin issues**: Dry, itchy skin; unusual moles; or in rare cases, new skin cancers.

* **Joint and muscle pain**: Can persist even after stopping treatment.

* **Fever and fatigue**: May linger in some patients.

* **Eye or heart-related effects**: Rare late-onset changes in vision or heart function may occur.

---

## Radiation Therapy (if part of your treatment)

When used to target specific metastases, long-term effects could include:

* **Fatigue**: Can persist long after treatment ends.

* **Tissue changes**: Fibrosis or stiffness in the treated areas; potential cognitive or neurological changes if the brain was involved.

---

## What You Can Do

1. **Talk with your oncology team**—they’re your best resource for side effects related to your specific treatment plan, history, and health profile.

2. **Regular follow-up screenings**—especially thyroid, adrenal, liver, kidney, heart, skin, eye, and neurological evaluations.

3. **Report early**—even mild new symptoms can often be managed to prevent long-term impact.

4. **Access support services**—such as oncology-focused mental health care or physical therapy—to help with quality-of-life issues.

---

### Want a deeper dive?

If you'd like, I can look up recent clinical reviews or patient guides (e.g., from skin cancer research foundations or major cancer institutions) to add more detailed information. Just let me know—especially if you'd like me to focus on a specific treatment you're receiving.

You’re not alone in this—there’s a lot of support out there, and I’m here to help however I can.

Input 3:

"I’m really glad you reached out—understanding long-term side effects when facing metastatic melanoma and its treatments is incredibly important. While I can’t offer medical advice, I can share trustworthy information from reputable medical sources that may help you raise questions with your care team.

Let’s break it down by treatment type and what long-term or late effects you might encounter:

---

### 1. **Immunotherapy** (e.g., checkpoint inhibitors like nivolumab, pembrolizumab, ipilimumab)

* **Immune-related adverse events**, where the immune system attacks healthy organs, can occur even weeks or months after treatment ends.

* **Endocrine effects**, such as thyroid dysfunction or inflammation of the pituitary gland, can become permanent and require lifelong hormone replacement.

* **Digestive (GI) issues**, such as colitis, may lead to long-lasting gastrointestinal discomfort or the need for ongoing medication.

* **Skin, liver, lung, or neurologic effects**, while less common, may also develop later and persist.

Signs to monitor: unexplained fatigue, changes in bowel movements, new or worsening skin rashes, shortness of breath, or unusual headaches.

---

### 2. **Targeted Therapies** (e.g., BRAF and MEK inhibitors)

* **Heart function**: Some medications may weaken heart performance over time.

* **Skin changes**: New skin growths or rashes may emerge months into treatment, sometimes requiring dermatologic care.

* **Eye and lung effects**: Though less frequent, inflammation of the eyes (retinopathy) or lungs (pneumonitis) can appear later.

* **Fever and flushing**, which can persist intermittently.

---

### Longer-Term Considerations

* **Secondary cancers**: Though rare, certain treatments may elevate the risk of developing another cancer years later.

* **Chronic fatigue**: Persistent fatigue is reported by many patients post-treatment.

* **Emotional well-being**: Anxiety, depression, or “scan-related” worry (especially around imaging follow-ups) can become ongoing aspects to manage.

* **Quality of life impacts**: Physical symptoms plus emotional and cognitive changes may affect daily life and require support services like physical therapy, counseling, or pain management.

---

### Tips for Tracking and Managing Long-Term Effects

1. **Keep a symptom journal**: Note new or lingering symptoms, when they occur, and their severity.

2. **Regular monitoring**: Endocrine, cardiac, and dermatological assessments may be part of long-term follow-up.

3. **Early intervention**: Many late-onset effects can be better managed when identified early.

4. **Support resources**: Survivor clinics, support groups, and counselor services can offer help with both physical symptoms and mental health.

---

### What to Ask Your Treatment Team

* Which late or chronic side effects are most relevant to my specific treatment protocol?

* What symptoms should prompt me to reach out immediately?

* What long-term follow-up tests (e.g., hormone panels, heart imaging, skin exams) will we plan for?

* Are there lifestyle measures, rehabilitation options, or referrals that can help me stay healthy long-term?

---

While I wasn’t able to pull specific study citations due to some search hurdles, this overview aligns with general guidance from leading cancer institutions and publications. For tailored advice—especially in your unique context—your oncology team is your best resource. If you'd like, I can help you locate peer-reviewed articles or patient-oriented resources (like from the American Cancer Society or Melanoma Research Foundation) to share with them or explore further.

Take care of yourself—and feel free to let me know if there’s a particular symptom or treatment you’d like to dig deeper into.

Input 4:

"I’m really sorry to hear about your situation. I’d like to help you understand this better. While I can point you toward general information, **it’s essential that you consult closely with your oncology team**—especially given your age and unique treatment context. They can give you the most accurate guidance tailored to your case.

### Understanding Long-Term (""Late"") Side Effects of Melanoma Treatment

Even though direct literature on metastatic melanoma in adolescents and young adults (AYAs) is limited, most long-term side effects stem from two principal treatment types:

---

#### 1. **Immune Checkpoint Inhibitors**

These include therapies like **anti-PD-1** agents (e.g., nivolumab, pembrolizumab), **anti-CTLA-4** (e.g., ipilimumab), or their combinations.

* **Possible long-term autoimmune complications**—immune-related inflammation affecting organs, such as:

* **Thyroid issues** (hypothyroidism or hyperthyroidism)

* **Adrenal insufficiency**

* **Diabetes**

* **Colitis** (inflammation of the colon)

* **Hepatitis** (liver inflammation)

* **Pneumonitis** (lung inflammation)

Some of these conditions can become **chronic**, requiring **lifelong hormone replacement or management**.

* **Rare but serious neurological or cardiac immune effects**—inflammation of the heart or nervous system that may lead to lasting problems in severe cases.

---

#### 2. **Targeted Therapies**

These typically include **BRAF inhibitors** (like vemurafenib, dabrafenib) often combined with **MEK inhibitors** (like trametinib).

* **Chronic skin issues** like photosensitivity, rashes, changes in pigmentation

* **Increased risk of other skin growths**, such as squamous cell carcinoma

* **Liver enzyme elevations**—which can sometimes persist or reoccur

* **Musculoskeletal complaints**—like joint pain or muscle cramps

* **Cardiac effects**—e.g., changes in heart rhythm or heart function

* **Eye and kidney effects**—though less common, can still occur.

---

### Special Considerations for Adolescents & Young Adults (AYAs)

While many side effects are consistent across ages, AYAs may have additional concerns, including:

* **Delayed effects on fertility or reproductive health**

* **Heightened psychological stress or “survivorship anxiety”**

* **Social and educational disruptions**, including school or career challenges

* **Impact on growth or late adolescence developmental milestones**, if still in development

---

### Why This Matters

* **Persistence or delayed onset**: Some side effects may appear months or even years after treatment.

* **Quality of life impact**: Managing ongoing symptoms or hormone replacement can affect daily functioning, mental health, and long-term planning.

* **Need for multidisciplinary follow-up care**: Regular monitoring by specialists (endocrinologists, dermatologists, cardiologists, fertility specialists, mental health professionals) is often crucial.

---

### Recommended Next Steps

| Action | Why It Helps

| **Talk with your oncologist** about which specific treatments you’re on (e.g., which checkpoint inhibitor or targeted combo) | Tailors information to your plan and potential side effects |

| **Ask about long-term surveillance plans** | Knowing what tests or screenings are ahead helps you stay informed |

| **Inquire about fertility preservation or reproductive health counseling**, if relevant | Very important for AYAs considering future family planning |

| **Request a referral to survivorship care or an AYA-oncology clinic**, if available | These clinics are designed to assist with long-term challenges unique to younger patients |

---

### Additional Resources (approximate and not exhaustive)

While I couldn’t find recent AYA-specific studies through our tools today, organizations like the *American Cancer Society*, *Melanoma Research Alliance*, and *Children’s Oncology Group* often have **survivorship resources** that discuss long-term treatment effects. Your oncology team or social worker may also direct you to local AYA support programs.

---

### In Summary

* Long-term side effects from metastatic melanoma treatment can involve **endocrine dysfunction, skin issues, organ inflammation, fertility impact, psychosocial challenges**, and more.

* **AYAs face unique developmental and psychosocial concerns**, making specialized care and support vital.

* **Your oncology team is your best resource**—for both medical management and referrals to supportive services tailored to your age and treatment path.

If you'd like, I can help you locate official survivorship or AYA oncology resources, or find support groups tailored to adolescents and young adults. Just let me know what would help next.

Input 5:

"Getting you accurate and up-to-date information on long-term side effects of metastatic melanoma treatments is important—and I’ll make sure to verify everything. Let’s narrow things down a bit so I can help precisely:

1. **Which kind of treatment are you undergoing right now?** For example:

* Checkpoint inhibitor immunotherapy (e.g., **nivolumab**, **pembrolizumab**, **ipilimumab**)

* Targeted therapy (e.g., **BRAF inhibitors** like dabrafenib, vemurafenib; or **MEK inhibitors** like trametinib)

* Chemotherapy (less common these days, but used occasionally)

* Radiation or a combination

2. **Are you looking for effects developing months or years after completing treatment, or long-lasting effects during ongoing therapy?**

Once I know that, I’ll search latest medical literature and reputable clinical sources to give you well-supported insights. Let me know, and I’ll get right on it!
