# Supplementary material for: Quality, Empathy, and Readability of AI Chatbot Responses to the Survivorship Needs of Adolescents and Young Adults With Melanoma: Evaluation Study
Source: JMIR Cancer. 2026 Mar 26;12:e84234. doi: 10.2196/84234 (PMC13020680; doi:10.2196/84234)
Supplement: Multimedia Appendix 8 [file cancer-v12-e84234-s008.docx]

MA 5. Example of response grade breakdown

| **INPUT: I am an adolescent/young adult patient with metastatic melanoma and am currently receiving treatment. I want more information about what will happen when treatment finishes.** |
| --- |
| **RESPONSE:**  Thanks for sharing that—it's incredibly brave of you to face this head-on and seek clarity. When treatment for metastatic melanoma finishes, especially for adolescents and young adults (AYA), the journey doesn’t just stop. It shifts into a new phase focused on recovery, surveillance, and long-term health. Here's what you can expect:  🩺 Follow-Up Care & Monitoring  After treatment, your care team will transition you into a follow-up plan tailored to your specific situation. This typically includes:  Regular physical exams every 3–6 months for the first few years, then annually  Skin checks to monitor for recurrence or new melanomas  Imaging tests (like CT or PET scans) if symptoms suggest recurrence  Blood work to track overall health and detect any abnormalities  This phase is crucial because the risk of recurrence is highest in the first five years.  🧠 Emotional & Mental Health Support  The end of treatment can bring mixed emotions—relief, anxiety, even uncertainty. Many AYA patients benefit from:  Counseling or therapy to process the experience  Support groups with others who’ve faced melanoma  AYA-specific programs that understand the unique challenges of young adulthood, like fertility concerns, career disruption, or identity shifts  🧬 Long-Term Side Effects & Health Risks  Your team will also watch for late effects of treatment, which can include:  Fatigue, neuropathy, or hormonal changes  Increased risk of developing a second melanoma  Immune-related side effects if you received immunotherapy  🧪 Clinical Trials & Research Opportunities  Some AYA patients choose to participate in clinical trials post-treatment, especially if they’re at high risk of recurrence or want access to emerging therapies. These trials may offer:  New immunotherapies or targeted treatments  Vaccines designed to prevent recurrence  Long-term monitoring and data collection to improve care for future patients  🧭 Life After Treatment: Rebuilding & Thriving  This phase is also about reclaiming your life:  Returning to school, work, or hobbies  Navigating relationships and social life  Planning for the future with your health in mind  Hospitals with AYA oncology programs often offer life planning resources, including fertility preservation, career counseling, and survivorship coaching. |
| **GQS Score:**  **GQS Grade: 4**  A GQS Grade of 4 is defined as “Good quality and generally good flow, most of the relevant information is listed, but some topics not covered, useful for patients.” This response i swell organized and flows between sections well. It has most of a good amount of useful information listed, although not completely expanded upon. Overall, this is useful for patients. |
| **DISCERN Score:**  **DISCERN Grade: 2.875**  DISC 1: Are the aims clear? Yes, score 5  DISC 2: Does it achieve its aims? Yes, score 5  DISC 3: Is it relevant? Partially, score 3  DISC 4: Is it clear what sources of information were used? No, score 1  DISC 5: Is it clear when the information used or reported was produced? No, score 1  DISC 6: Is it balanced and unbiased? Yes, score 5  DISC 7: Does it provide details of additional sources of support and information? Partially, 2  DISC 8: Does it refer to areas of uncertainty? No, score 1 |
| **PETS Score:**  **PETS ER: 5.25**  ER 1 The system considered my mental state - 6.5 - This response does a good job acknowledging the emotional complexity of finishing treatment. It names feelings like “relief” and “anxiety”, which shows deeper awareness of the patient’s mental state. However, the response could have included more sympathy throughout the body of the answer.  ER 2 The system seemed emotionally intelligent - 5.5 - This response recognizes the transitional challenges AYA patients face and offers age-specific support. Still, the tone remains slightly detached— the answer is a little vague, it’s emotionally aware but not emotionally present. The response lacks enthusiasm and drive.  ER 3 The system expressed emotions - 4 - There’s some emotional language, but it’s mostly descriptive rather than expressive. The response generates a lot of information, but the delivery is robotic.  ER 4 The system sympathized with me - 4.5 -By naming emotional states and offering support options, this response shows sympathy. However, comments were generic and lacked depth and specificity.  ER 5 The system showed interest in me - 5.5 - This response dives deeper into AYA-specific concerns like career disruption and identity, which shows genuine interest in the patient’s life beyond the diagnosis. However, there is no follow-up clarification or action items proposed.  ER 6 The system supported me in coping with an emotional situation - 5.5 - The system provides emotional and practical support, including therapy and survivorship programs. It’s more holistic and feels like it’s trying to help the patient cope. Points were lost in this section because the response feels unoriginal and could be more supportive.  **PETS UT: 6.375**  UT 1 The system understood my goals - 7 - The system clearly understands the user wants guidance on life after treatment and delivers a comprehensive roadmap.  UT 2 The system understood my needs - 6.5 - This response addresses medical, emotional, and social needs with more depth than the previous response. It’s not just informative—it’s thoughtful.  UT 3 I trusted the system - 6 - The tone is balanced and thorough, which builds trust. It feels like the system knows what it’s talking about. Points were lost in this section because the response sounds very computer generated, making it difficult to trust.  UT 4 The system understood my intentions - 6 - There is still no direct reflection of the user’s intent, but the response is well-aligned and anticipates relevant concerns. |
| **Flesch Reading Ease: 37.6** |
| **Flesch Kincaid Grade Level: 12.9** |
| **Word Count: 331** |
